# Interspecific interactions through 2 million years: are competitive outcomes predictable?

**DOI:** 10.1098/rspb.2016.0981

**Published:** 2016-08-31

**Authors:** Lee Hsiang Liow, Emanuela Di Martino, Kjetil Lysne Voje, Seabourne Rust, Paul D. Taylor

**Affiliations:** 1 Centre for Ecological and Evolutionary Synthesis, Department of Biosciences, University of Oslo, Oslo, Norway; 2 Natural History Museum, University of Oslo, Oslo, Norway; 3 Department of Earth Sciences, Natural History Museum, Cromwell Road, SW7 5BD London, UK; 4 6702 State Highway, 12 RD 3, Kaikohe 0473, New Zealand

**Keywords:** ecological interactions, spatial competition, cheilostome bryozoans, Pleistocene

## Abstract

Ecological interactions affect the survival and reproduction of individuals. However, ecological interactions are notoriously difficult to measure in extinct populations, hindering our understanding of how the outcomes of interactions such as competition vary in time and influence long-term evolutionary changes. Here, the outcomes of spatial competition in a temporally continuous community over evolutionary timescales are presented for the first time. Our research domain is encrusting cheilostome bryozoans from the Wanganui Basin of New Zealand over a *ca* 2 Myr time period (Pleistocene to Recent). We find that a subset of species can be identified as consistent winners, and others as consistent losers, in the sense that they win or lose interspecific competitive encounters statistically more often than the null hypothesis of 50%. Most species do not improve or worsen in their competitive abilities through the 2 Myr period, but a minority of species are winners in some intervals and losers in others. We found that conspecifics tend to cluster spatially and interact more often than expected under a null hypothesis: most of these are stand-off interactions where the two colonies involved stopped growing at edges of encounter. Counterintuitively, competitive ability has no bearing on ecological dominance.

## 1. Introduction

Interactions among organisms have implications for the survival and reproduction of individuals and hence, ultimately, the survival and evolution of populations and species. While interactions such as sexual selection, competition, predator–prey relationships, disease and symbiosis are relatively easy to observe among living organisms, they have to be inferred for extinct species and populations, using modern analogues and relevant morphological and ecological information preserved in the fossil record. On the flip side, the evolutionary consequences of interactions observable among organisms are not easily extrapolated from short-term ecological observations. Because of such limitations of observations and extrapolations, the consequences of interactions, in particular interspecific competition, are commonly modelled using phylogenetic hypotheses [1] or inferred from character displacement [2,3]. Both of these approaches, while informative in their own right, have limitations, notably that ecological interactions on geological timescales are inferred rather than observed.

Abiotic factors are often purported to be the most important drivers of macroevolution on a geological timescale [4], even though such palaeontological studies seldom consider biotic factors as possible drivers [5]. Palaeontological studies of diversity dynamics and evolutionary changes are often skewed towards investigation of abiotic factors as drivers, not least because biotic interactions are notoriously difficult to quantify in the fossil record. Despite this difficulty, palaeontologists have attempted to infer changes in herbivory [6], predation [7,8] and parasitism [9]. However, because of the difficulty of identifying interacting taxa to a low taxonomic level, and/or achieving large sample sizes suitable for statistical analyses, we still have little quantitative understanding of how biotic interactions change through time. Here, for the first time to the best of our knowledge, species-level competitive interactions directly observable in the fossil record are used to investigate biotic interactions on macroevolutionary timescales.

Encrusting bryozoans offer a good system in which to study ecological interactions because their competitive overgrowths often fossilize [10]. Encrusting bryozoan larvae settle on substrates such as shells and rocks, metamorphose and begin colony development. When a growing bryozoan colony meets another encrusting organism, often another bryozoan, it may overgrow or be overgrown by that organism. Overgrowth generally kills the overgrown bryozoan zooids, which may be feeding and/or reproductive zooids, hence impacting survival and reproduction of the colony as a whole. Past studies on competitive overgrowth in bryozoans have focused on (i) the fossil record of overgrowth interactions at inter-clade level and (ii) among genera or species within living communities over very short timescales. At the inter-clade level, it has been hypothesized that bryozoans of the order Cyclostomata were poorer competitors than those of the order Cheilostomata [11–13] and that the competitive advantage of cheilostomes has contributed to their higher species diversities. Studies of living communities over a few seasons or years have focused on questions of competitive intransitivity, where competitiveness is a simple hierarchy [14,15], spatial variation in the sense of differential latitudinal outcomes [16,17] and substrate use [18]. Here, we present a novel investigation of species–species overgrowth interactions and ask whether competitive outcomes have changed on a macroevolutionary timescale.

Using samples from one of the most complete shallow-water marine Pleistocene sequences known in the world [19], we answer the following questions.

(1) Are given species consistently winners or losers through time?
(2) Do species become better at winning competitive overgrowths through time?
(3) Do genus-level analyses reflect species-level overgrowth results, or are genera made up of both winner and loser species?
(4) Do species engage in overgrowth competition more frequently with conspecifics?
(5) Are ecological commonness and competitive ability correlated?

## 2. Material and methods

Material for this study was collected in January 2014 from Pleistocene strata cropping out along coastal cliffs and river valleys, northwest of Whanganui city, North Island, New Zealand. The Wanganui Basin is a proto back-arc basin filled by several kilometres of predominantly shelf siliciclastic sediments, comprising sandstones, siltstone, mudstones, locally carbonate-rich shell beds and volcanic ash layers, forming a cyclic depositional sequence record spanning the last *ca* 2 Myr with a well-established, high-resolution chronostratigraphy [19–22]. We collected material only from shellbeds in shallow-shelf deposited transgressive systems tracts (TST) that were reported as yielding bryozoan-encrusted shells [23] to minimize environmental differences among samples (electronic supplementary material, table S1). The sampled TSTs are typically siliciclastic sand-rich deposits up to several metres thick.

Bivalves are by far the most common macroscopic components of the shellbeds we targeted [24,25]. We collected as many bivalve shells as possible that contained cheilostome–cheilostome interactions observable with a hand-lens in the field. The stratigraphic levels of the source horizons and GPS positions were noted. We also studied dredge samples of encrusted bivalves from nearby Cook Strait as modern analogues of our fossil samples [26].

**Figure 1.**
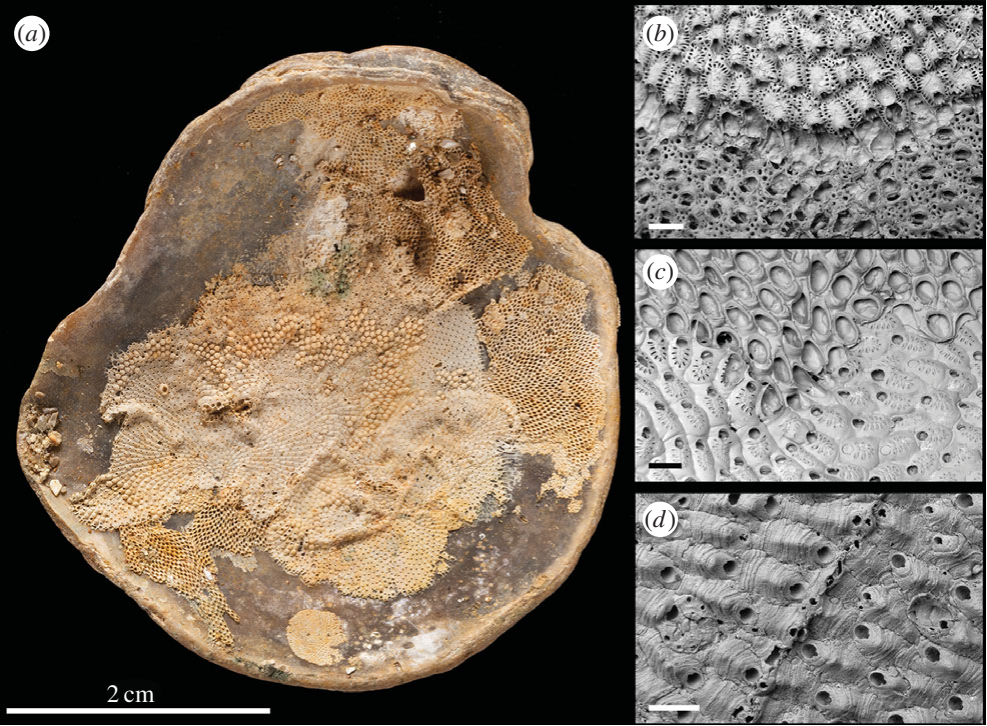
Competitive interactions between encrusting bryozoan colonies. (*a*) An encrusted shell from the Nukumaru Limestone Formation NHMUK PI BZ 7744. (*b*) Win–lose competition between *Escharoides excavata* (top) and *Arachnopusia unicornis* (bottom). (*c*) Reciprocal overgrowth between *Valdemunitella valdemunita* (top) and *Figularia carinata* (bottom). (*d*) Stand-off competition between two colonies of *Antarctothoa tongima*. Scale bars for *b* and *c* = 500 µm, *d* = 200 µm.

Before examining the encrusting bryozoan colonies, the shell substrates were cleaned using one or a combination of the following techniques depending on fragility: tapping to remove sediment, gentle washing under running water, scrubbing with a soft toothbrush and washing in an ultrasonic bath. Each shell, colony and interaction was allocated a unique number in our database of interactions. Bryozoan colonies were identified to species level whenever possible, using a stereomicroscope. The majority of our Pleistocene fossil taxa can still be found living in the Wanganui area today [23]. In a minority of cases, species-level identification was not possible, either because of deficient preservation or limited stereomicroscopic resolution (see Discussion). All cheilostome–cheilostome contest interactions (both interspecific and intraspecific) were recorded and classified as one of the following types: (i) win–lose overgrowths, whenever the growing edge of the winner colony is observed to cover an orifice or orifices of zooids in the losing colony [14,27]; (ii) reciprocal overgrowths, when both competitors mutually overgrow each other; (iii) stand-offs, where two competing colonies abut without overgrowth at the encounter edge (figure 1). We also recorded fouling where one of the colonies settled on the surface of another. Stand-offs and reciprocal overgrowths necessarily happen *syn-vivo*, while observations of win–lose interactions may result from a *syn-vivo* interaction or overgrowth after death. Fouling, on the other hand, often happens *post-mortem* [10]. Because proportions of fouling are low and stand-offs high (see Results and Discussion sections), we assume that our sampled communities are largely contemporaneous. Previous studies comparing ecological and palaeoecological communities have also shown that instances of overgrowth after death contribute noise but not signal to overgrowth interaction data [28].

We examined a total of 751 shells, encrusted by 58 cheilostome taxa identifiable to genus level and 76 to species level, including seven species that are yet to be named and excluding *Hippothoa flagellum*, the only cheilostome in our data that is a runner (i.e. a linear branching encrustation) rather than a sheet. The following analyses are based on five Pleistocene formations plus Recent dredge samples (electronic supplementary material, table S1), comprising 7088 cheilostome–cheilostome contest interactions, of which both colonies of 6924 interacting pairs could be identified to genus level and 4800 could be identified to species level. A summary of our data is given in electronic supplementary material, table S1.

To explore whether a given taxon is a winner or loser at any given time interval, we modelled wins and losses as binomial trials [29]. To test whether win-proportions change for the same taxon through the time slices, we used Fisher's exact test [30] and examined resulting *p*-values using both the more conservative Bonferroni's correction and the less conservative false discovery rate control [31] for multiple comparisons. We randomized our data by (i) sampling and randomly pairing colonies from our original data and then (ii) randomly assigning interactions without replacements to these randomized pairs of colonies, in order to generate null expectations of the distributions of interactions among taxa. We then used the Mantel–Haenszel test [32], an extension of a χ^2^-test, for comparing simulated and original contingency tables of overgrowth interactions. To compare species and genus overgrowth patterns, we calculated average outcomes of interspecific interactions based on all colonies assigned to a given genus represented by more than one species and also genus averages from congeneric species averages. All statistical analyses were conducted in R v. 3.2.0 [33] and code and data are supplied in the electronic supplementary material.

## 3. Results

### (a) Is any given species consistently a winner or loser through time?

**Figure 2.**
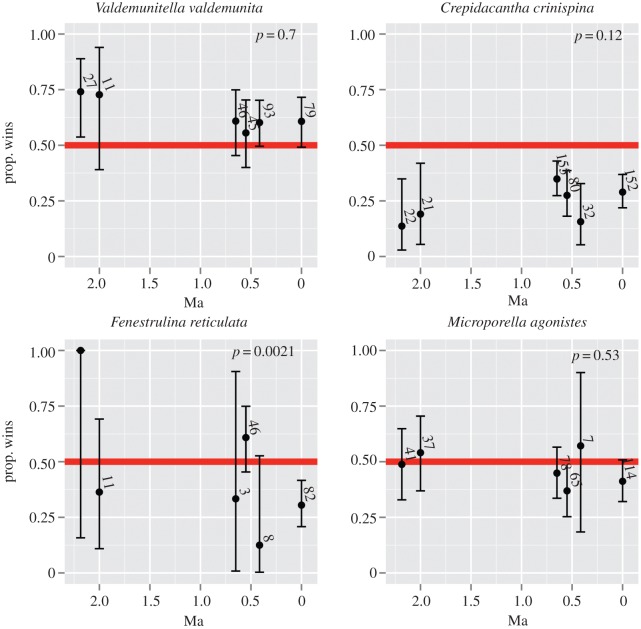
Interspecific win-proportions through time. Each panel plots the binomial probabilities and 95% confidence intervals of the interspecific win-proportions for the named species (other species are plotted in electronic supplementary material, figure S1). Red horizontal lines indicate the null hypothesis of 0.5 win-proportions. *P*-values stem from Fisher's exact test to compare differences among the win-proportions among binomial probabilities in each panel. Slanted numbers are the number of interspecific interactions contributing to plotted points and the associated confidence intervals.

Figure 2 shows examples of binomial probability plots and confidence intervals [29] for selected species in interspecific win–lose overgrowth interactions where both colonies are identified to species level. Some species appear to be consistent winners (e.g. *Valdemunitella valdemunita*), while others are clear consistent losers (e.g. *Crepidacantha crinispina* in which binomial confidence intervals never cross the 0.5 line). In yet other species, a combination of small sample sizes for certain time intervals and likely genuine changes in competitiveness lead to wide fluctuations of observed wins (*Fenestrulina reticulata*), while in others, wins and losses seem equally likely throughout (*Microporella agonistes*)*.* These results, based on thousands of interactions, suggest that there is strong interspecific variation in competitive ability (see electronic supplementary material, figure S1 for other species).

### (b) Do species become better at winning competitive overgrowths through time?

Continuing with colonies identified to species-level engaged in interspecific win–lose interactions, we investigated if each species maintained the same win-proportion through the six time intervals. In the majority of the species, win-proportions are indistinguishable through the six time intervals. Given that each species interacts with multiple other species and that each species dataset (as shown in figure 2; electronic supplementary material, figure S1) is not independent, we used both a conservative Bonferroni's correction and a false discovery rate control at 5% type I error rate. Of species that were observed winning or losing in at least two time intervals, only three species (*Steginoporella magnifica*, *Parasmittina aotea*, *Chaperia granulosa*; electronic supplementary material, figure S1) changed their competitiveness through time by both criteria, leaving little evidence that species-level competitive outcomes change over time.

### (c) Do genus-level analyses reflect species-level overgrowth results or are genera made up of both winner and loser species?

Using colonies identified to genus level, including those colonies for which species identity cannot be confirmed (electronic supplementary material, table S1), we present equivalent results from genus-level win–lose interactions using binomial probabilities and *p*-values from Fisher's exact test as above (electronic supplementary material, figure S2). As in the species-level analysis above, some genera (represented by more than one species in our win–lose interaction data) seem to be clear winners (e.g. *Escharoides*, *Valdemunitella*), while other genera are equivocal (electronic supplementary material, figure S2). We cannot clearly identify any genus that is a loser through the time intervals investigated. *Microporella*, *Fenestrulina* and *Parasmittina* emerge as genera that have temporally varying competitive abilities, based on both Bonferroni's and false positive rate adjustments.

**Figure 3.**
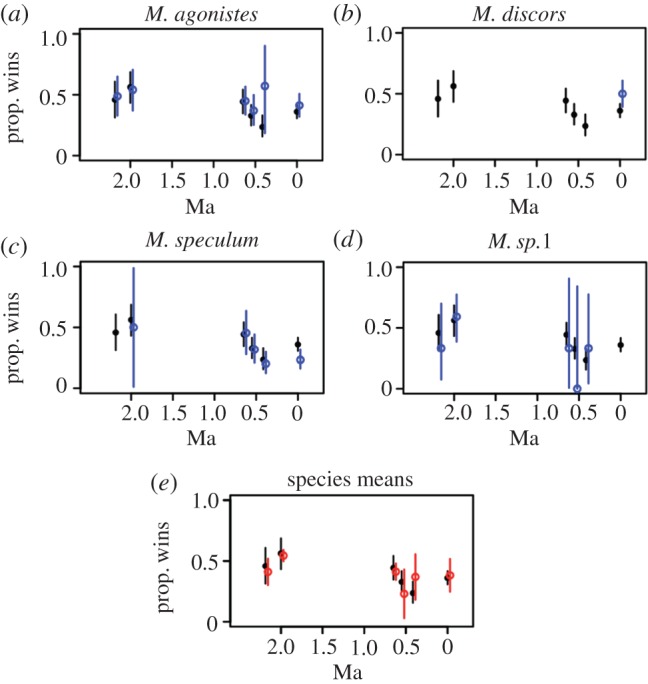
Genus versus species competition dynamics: *Microporella*. As in figure 2, panels plot the binomial probabilities (circles) and 95% confidence intervals (lines) of interspecific win-proportions. Black lines and circles indicate win-proportions for all *Microporella* regardless of species while blue lines and open circles indicate the win-proportions of the named species. In figure 3*e*, the average of species averages and their standard deviations are plotted in red for comparison.

Most of these 15 multi-species genera are represented only by two species in multiple time slices, making it unreasonable to undertake cross species and time comparisons to address the question whether genus dynamics reflect species dynamics. In two of the genera, *Microporella* and *Smittina*, we can see how species dynamics contribute to genus dynamics (figure 3; electronic supplementary material, figure S3). It is difficult to generalize from only two cases, but individual species within these genera do not contribute in the same way to genus patterns. For instance, *Microporella* appears to be a loser closer to the Recent, although this is mainly due to the contributions of *M. speculum*, while *M. agonistes* has always been more even in its competitive abilities through time. The average competitive ability of *Microporella* also depends in part on interpretation: win-proportions tabulated using species means (red in figure 3*e*) are not the same as those tabulated using all *Microporella* interactions, especially in the two youngest intervals (Shakespeare Cliff Sand Basal Shellbed and Lower Castlecliff Shellbed) before the Recent.

### (d) Do species engage in overgrowth competition more frequently with conspecifics?

Within species, most interactions are win–lose (0.677 ± 0.018 s.e., *N* = 72), as when averaged among all species within each time slice (0.685 ± 0.016 s.e., *N* = 6).

Stand-offs (0.212 ± 0.018 s.e., *N* = 72; 0.200 ± 0.020 s.e., *N* = 6) are much fewer and reciprocal interactions (0.015 ± 0.003, *N* = 72; 0.018 ± 0.006 s.e., *N* = 6) almost non-existent. For each time interval sampled, we examined whether species showed differences among interspecific versus intraspecific and stand-off versus win–lose outcomes that are statistically different from a null expectation. To do so, we generated 1000 randomized datasets and compared these with the observed dataset. For those species whose interactions were statistically different from a null distribution of interactions (electronic supplementary material, table S2), it is because both the number of cases of intraspecific win–lose and stand-offs are consistently greater than a null expectation regardless of species or time interval. Interspecific stand-offs are most often fewer than expected while interspecific win–lose can be either more frequent or less so than expected. This implies that intraspecific interactions (both stand-offs and win–lose, some of which may have occurred *post-mortem*) are more common than expected, implying spatial clustering of conspecifics. These results also indicate that intraspecific stand-offs are much more common than interspecific stand-offs.

### (e) Are ecological commonness and competitive ability correlated?

For each time interval, we compared the win-proportion (means plotted in figure 2; electronic supplementary material, figure S1) for each species with the number of observed colonies of the same species. The latter is not an unbiased estimate of true ecological commonness as the number of observed colonies is likely confounded by sampling. Achieving unbiased estimates of true relative abundance is quite involved and we postpone that exercise to a future paper. However, taking the number of unique colonies observed as a rough estimate of relative abundance, we observe that competitiveness as measured by a binomial win–lose proportion is not correlated with observed ecological abundance in any of our sample time intervals (electronic supplementary material, table S3).

## 4. Discussion and conclusion

Our study is, to the best of our knowledge, the first to track species-specific competitive abilities of a community that has been present continuously through geological time. It builds on the observations of competitive interactions among bryozoans through several decades of study [34–40] as well as insights from other systems, especially plant communities [41,42] and other sessile invertebrate communities [43,44]. A direct measurement of competition is more satisfactory than using proxies such as abundance to infer competition (e.g. [45]) as higher abundance does not necessarily result simply from increased competitiveness [46].

Heterogeneity, such as that simply arising from site differences, can lead to differences in competitive outcomes as observed in some bryozoan communities [47]. In our study, we have not attempted to tease apart site-specific differences in competitive outcomes, for the simple reason that our data, despite being rich, are not rich enough, relative to the high species richness of the fauna. Our inferences for each time interval are hence averaged across sites and time, since each sampled time interval encompasses thousands of years (electronic supplementary material, table S1). However, time-averaging in fossil data is not necessarily a disadvantage given the questions we are asking. In fact, time-averaging might help to filter out short-term variations that do not contribute substantially to long-term dynamics [48]. Whereas ecological data collected over numerous years have shown that interaction strengths can change depending on the physical environment and the presence of grazers (e.g. for crustose coralline algae [43], but see [44] for a counterexample where interactions were not context dependent), our time-averaged samples indicate that general patterns can be discerned. There were very few species in our study system that changed their winning proportions in any significant manner through 2 Myr of their evolution, suggesting stability in competitive abilities on the timescales of hundreds of thousand generations. Despite heterogeneity in our system stemming from numerous factors, including time, a changing climate, substrate availability and community composition, we were able to quantify temporal dynamics in win-proportions and identify encrusting bryozoan species that are clear winners and others that are clear losers.

We chose a study palaeontological system in which we were able to identify most of the colonies to species level. In many palaeontological studies, including those asking questions about taxon richness and spatial distribution, the genus is often used as a proxy for the species. In some cases, this can be justified [49,50], but in others it is less clear on both empirical and conceptual grounds [51]. This study is the first to examine whether the competitive abilities of species within a given genus reflect average genus-level temporal dynamics on geological timescales. Given that there were only two genera in which we could observe species dynamics over multiple time intervals, we cautiously and tentatively conclude that species idiosyncratically contribute to genus patterns when it comes to competitive abilities, rendering the genus proxy an inappropriate one for individual species-specific questions on competitiveness.

There is rather strong clustering of interaction outcomes between interspecific and intraspecific interactions. There are more intraspecific stand-off interactions than expected in the species and time intervals for which data were sufficient to make such a comparison. This observation gives us confidence that our samples capture a majority of live–live (*syn-vivo*) interactions (see [10]), because stand-off interactions cannot occur when one party is dead. There are also fewer interspecific stand-off interactions than expected by chance, indicating some predictability in interaction outcomes, even though our data are currently not rich enough to statistically examine specific species–species interactions in detail. For species that deviate from a null expectation for win–lose and stand-off interactions, most also interact more than expected. This may imply temporal segregation, ecological clustering and mechanisms for attracting or repelling realized interactions.

Ecological abundance does not seem to be related to competitiveness in any straightforward way in our system, corroborating findings in some living assemblages of bryozoans. For example, Centurion & Gappa [40] reported a negative correlation between competitive ability (defined as win/lose ratios) and the number of observed colonies. This negative relationship resonates with theoretical observations that poor competitors can be more abundant [46] and vice versa. In our system, for instance, *Escharoides excavata* is a good competitor and very common in the earliest formation in our dataset, yet it ‘disappeared’ from the Wanganui Basin for almost 2 Myr before ‘reappearing’ in our modern samples from Cook Strait. *Crepidacantha crinispina* is a consistent loser, yet it is commonly present throughout the 2 Myr. As already mentioned, we do not purport to have reliably estimated unbiased relative abundance but emphasize that proper statistical estimation has to be developed to infer ecological abundance, so we leave this discussion as tentative.

Other factors important for survival, which have not been measured in this study, must also be operating, although how much each of these contribute to the abundance of a species at any one time remains an open question. These factors include fecundity, larval recruitment, colony growth rate [18,52], age structure [53], colony size [54], growth form [37] and ecological successions and seasonal resource use, nature of the substrate [40], other biotic interactions types (predation, disease, symbiosis) and other competing taxa (cyclostome bryozoans, sponges, worms, foramifera, etc.). Other caveats to our conclusions, some of which we have already discussed, must be kept in mind. First, even though we have the largest dataset ever amassed for studying spatial competition in bryozoans, each species-specific interaction is still rare within each time interval. More specifically, our inability to reject null hypotheses (figures 2; electronic supplementary material S1 and S2) may in part be attributed to small sample sizes. In addition, we are not able to justify the use of classic intransitivity metrics [15] to study changes in competitive networks/hierarchies through time, although we have very strong suspicions that our species-rich bryozoan communities are intransitive though time [42]. Second, while we made our best effort to control for environmental differences among the time intervals we examined by selecting similar depositional environments, environmental variation remains [23]. Third, while the stand-off and reciprocal interactions in our fossil samples give us confidence that some of the win–lose interactions must have happened *syn-vivo*, our data are still coloured by an unknown proportion of live–dead interactions. Although we have clear winners and losers, indicating that more than random settling order is at work in our system, it is still possible that winners are consistently latecomers in the ecological succession on the substrate. Fourth, even though we have structured our arguments around spatial competition, we cannot rule out the hypotheses that competition for food [35,55], or oxygen [56], or non-contact competition [57] are also important.

Our study of an evolutionarily continuous community of spatially competing encrusting organisms through more than 2 Myr of geological time has allowed us to show that species retain their competitive abilities: some bryozoan species appear to be consistent winners, others consistent losers. What traits characterize a good competitor? A future approach will be to study the distribution of traits in winners versus losers in order to understand, mechanistically, which might facilitate competition, and ultimately, species coexistence in this relatively species-rich system [58]. Many other important ecological and evolutionary questions can be addressed using both the living and fossilized bryozoan fauna from the Wanganui Basin.

## Supplementary Material

Liow et al. ESM Tables S1 S2 S3 Figures S1 S2 S3

## Supplementary Material

Wanganui_Cheilostome_interactions.Rdata

## Supplementary Material

Wanganui_Cheilostome_interactions.txt
