# Supplementary material for: Interspecific interactions through 2 million years: are competitive outcomes predictable?
Source: Proc Biol Sci. 2016 Aug 31;283(1837):20160981. doi: 10.1098/rspb.2016.0981 (PMC5013793; doi:10.1098/rspb.2016.0981)
Supplement: Liow et al. ESM Tables S1 S2 S3 Figures S1 S2 S3 [file rspb20160981supp1.pdf]

**Table S1: Summary of data on cheilostome-cheilostome interactions.**

The number of inter- and intraspecific win-lose (WL), standoff (SO), reciprocal (R) interactions and fouling (F) instances are shown for each time slice. Isotope stage numbers and approximate ages from Naish et al. (1998). Cook Strait samples are modern while Nukumaru Limestone samples are geologically the oldest.

| | Cook Strait | Shakespeare Cliff Sand Basal Shellbed | Lower Castlecliff Shellbed | Upper Kai-iwi Shellbed | Nukumaru Brown Sand | Nukumaru Limestone |
|---|---|---|---|---|---|---|
| Genera observed | 46 | 34 | 40 | 39 | 38 | 37 |
| Species observed | 55 | 38 | 43 | 44 | 41 | 38 |
| Number of colonies observed | 2239 | 702 | 477 | 892 | 632 | 512 |
| Shannon-Weaver index | 3.37 | 2.70 | 3.20 | 3.11 | 3.13 | 3.05 |
| WL, SO, R,F interactions scored | 2309 | 864 | 866 | 1423 | 879 | 747 |
| WL | 1491 | 497 | 547 | 945 | 545 | 490 |
| WL (%) | 64.57 | 57.52 | 63.16 | 66.41 | 62.00 | 65.60 |
| SO | 598 | 295 | 225 | 242 | 258 | 184 |
| SO(%) | 25.90 | 34.14 | 25.98 | 17.01 | 29.35 | 24.63 |
| R | 50 | 11 | 25 | 33 | 4 | 13 |
| R(%) | 2.17 | 1.27 | 2.89 | 2.32 | 0.46 | 1.74 |
| F | 170 | 61 | 69 | 203 | 72 | 60 |
| F(%) | 7.36 | 7.06 | 7.97 | 14.27 | 8.19 | 8.03 |
| Both partners identified to genus | 2286 | 851 | 846 | 1393 | 826 | 722 |
| Both partners identified to species | 1530 | 675 | 638 | 855 | 615 | 487 |
| Isotope Stage Number (Start) | NA | 12 | 14 | 16 | 77 | 89 |
| Isotope Stage (End) | NA | 12 | 14 | 16 | 73 | 79 |
| Start Age (MYA) | 0 | 0.43 | 0.58 | 0.68 | 2.03 | 2.29 |
| End Age (MYA) | 0 | 0.4 | 0.52 | 0.62 | 1.97 | 2.08 |

**Table S2: More intra-specific interactions than expected.**

Columns show the number of observed interspecific win-lose interactions (Obs. w/L), the average number of simulated (N=1000) interspecific win-lose interactions  (Sim.W/L) followed by the number of observed interspecific standoff interactions (Ob.So) and the average number of simulated interspecific standoff interactions. The next four columns show the respective numbers of intraspecific interactions. The last column shows the average p-values when comparing observed and simulated data from the Mantel-Haenszel test. Only species with simulated and observed data that are significantly different at a p<0.05 level (*) and those that are still significantly different after a Bonferoni correction (***) are presented.

| | Interspecific | | | | Intraspecific | | | | |
|---|---|---|---|---|---|---|---|---|---|
| **Nukumaru Limestone** | Obs.W/L | Sim.W/L | Ob.So | Sim. So | Obs.W/L | Sim.W/L | Ob.So | Sim. So | p-value (MH-test) |
| *Antarctothoa tongima**** | 56 | 35.07 | 5 | 15.71 | 9 | 1.83 | 31 | 0.78 | 1.69E-09 |
| *Escharella spinosissima** | 31 | 11.65 | 2 | 5.14 | 20 | 0.23 | 11 | 0.10 | 1.40E-02 |
| *Escharoides excavata** | 38 | 28.80 | 1 | 13.11 | 7 | 1.28 | 4 | 0.60 | 1.49E-02 |
| *Microporella agonistes** | 41 | 36.11 | 1 | 1.09 | 12 | 0.01 | 6 | 0.01 | 6.96E-03 |
| *Parasmittina aotea** | 13 | 33.15 | 0 | 6.19 | 4 | 0.28 | 4 | 0.12 | 1.17E-02 |
| *Steginoporella magnifica*** | 34 | 29.70 | 0 | 5.57 | 2 | 0.26 | 4 | 0.12 | 1.64E-04 |
| **Nukumaru Brown Sand** | | | | | | | | | |
| *Antarctothoa tongima**** | 77 | 46.057 | 7 | 18.79 | 13 | 2.41 | 14 | 1.01 | 9.37E-05 |
| *Fenestrulina* sp. 1* | 24 | 33.443 | 1 | 13.74 | 4 | 1.42 | 4 | 0.56 | 7.61E-03 |
| *Microporella agonistes** | 37 | 26.503 | 4 | 10.67 | 18 | 0.96 | 10 | 0.41 | 2.68E-02 |
| *Microporella* sp. 1* | 27 | 8.665 | 0 | 3.57 | 6 | 0.08 | 3 | 0.02 | 1.18E-02 |
| *Opaeophora lepida** | 9 | 22.484 | 0 | 9.38 | 1 | 0.67 | 2 | 0.26 | 4.55E-02 |
| *Steginoporella magnifica**** | 57 | 43.716 | 9 | 17.87 | 3 | 2.20 | 18 | 0.91 | 2.78E-07 |
| **Upper Kai-iwi Shellbed** | | | | | | | | | |
| *Antarctothoa tongima** | 58 | 34.355 | 6 | 9.85 | 4 | 0.97 | 4 | 0.29 | 2.23E-02 |
| *Celleporina* cf. *tubulata** | 64 | 30.494 | 0 | 8.83 | 13 | 0.76 | 4 | 0.22 | 9.07E-03 |
| *Crepidacantha crinispina**** | 155 | 55.7 | 6 | 15.94 | 36 | 2.72 | 11 | 0.74 | 4.64E-03 |
| *Microporella agonistes** | 78 | 40.718 | 1 | 11.43 | 23 | 1.39 | 6 | 0.37 | 9.53E-03 |
| **Lower Castlecliff Shellbed** | | | | | | | | | |
| *Aimulosia marsupium** | 34 | 21.724 | 2 | 8.75 | 7 | 0.51 | 4 | 0.22 | 2.79E-02 |
| *Calloporina angustipora** | 41 | 35.67 | 0 | 14.73 | 11 | 1.47 | 3 | 0.62 | 3.28E-02 |
| **Shakespeare Cliff Sand Basal Shellbed** | | | | | | | | | |
| *Aimulosia marsupium* | 40 | 12.578 | 0 | 8.55 | 5 | 0.22 | 2 | 0.16 | 1.94E-02 |
| *Antarctothoa tongima* | 39 | 69.327 | 9 | 46.31 | 2 | 9.60 | 157 | 6.45 | 7.08E-13 |
| *Calloporina angustipora* | 30 | 62.964 | 0 | 42.26 | 6 | 8.03 | 2 | 5.48 | 3.98E-02 |
| *Chaperia granulosa* | 28 | 22.932 | 0 | 15.38 | 2 | 0.76 | 6 | 0.49 | 1.44E-05 |
| *Crepidacantha crinispina* | 32 | 33.444 | 0 | 22.24 | 9 | 1.87 | 5 | 1.24 | 1.46E-03 |
| *Escharoides angela* | 69 | 43.207 | 0 | 28.88 | 9 | 3.78 | 3 | 2.47 | 2.28E-02 |
| *Microporella speculum* | 89 | 52.43 | 2 | 35.36 | 19 | 5.51 | 7 | 3.79 | 4.55E-03 |
| **Cook Strait** | | | | | | | | | |
| *Aimulosia marsupium** | 30 | 66.236 | 0 | 27.10 | 5 | 1.79 | 2 | 0.74 | 3.15E-02 |
| *Calloporina angustipora**** | 102 | 36.33 | 1 | 14.82 | 30 | 0.66 | 10 | 0.28 | 1.41E-04 |
| *Crepidacantha crinispina** | 152 | 83.321 | 4 | 33.96 | 95 | 2.70 | 18 | 1.15 | 2.56E-03 |
| *Escharella spinosissima** | 39 | 49.853 | 0 | 20.61 | 28 | 1.10 | 4 | 0.48 | 3.70E-02 |
| *Escharoides excavata** | 137 | 56.44 | 10 | 23.20 | 12 | 1.35 | 6 | 0.57 | 1.37E-02 |
| *Fenestrulina reticulata** | 82 | 55.76 | 0 | 22.71 | 40 | 1.25 | 4 | 0.55 | 4.56E-02 |
| *Figularia carinata**** | 66 | 77.669 | 3 | 31.81 | 9 | 2.42 | 10 | 0.94 | 1.99E-05 |
| *Smittina purpurea** | 5 | 67.352 | 2 | 27.77 | 6 | 1.92 | 23 | 0.77 | 1.80E-02 |

**Table S3: Commoness versus competitiveness**

Correlation between species interspecific win-proportions and observed abundance.

| | Spearman's rho | p |
|---|---|---|
| Nukumaru Limestone | 0.059 | 0.734 |
| Nukumaru Brown Sand | 0.576 | 0.739 |
| Upper Kai-iwi Shellbed | 0.085 | 0.624 |
| Lower Castlecliff Shellbed | 0.179 | 0.269 |
| Shakespeare Cliff Sand Basal Shellbed | 0.127 | 0.433 |
| Cook Strait | -0.347 | 0.832 |

**Fig. S1: Inter-specific win-proportions through time.**

As in main text Fig. 2, each panel plots the binomial probabilities and 95% confidence intervals of the interspecific win-proportions through Pleistocene - Recent time intervals. Red horizontal lines indicate the null hypothesis of 0.5 win-proportions. P-values stem from Fisher's Exact Test to test differences among the win-proportions among binomial probabilities in each panel. Slanted numbers are the number of interspecific interactions contributing to plotted points and the associated confidence intervals. In addition, 24 cheilostome species were not observed winning or losing an interspecific competition in our data, hence only 52 species are shown, four of these already presented in main text Fig. 2.

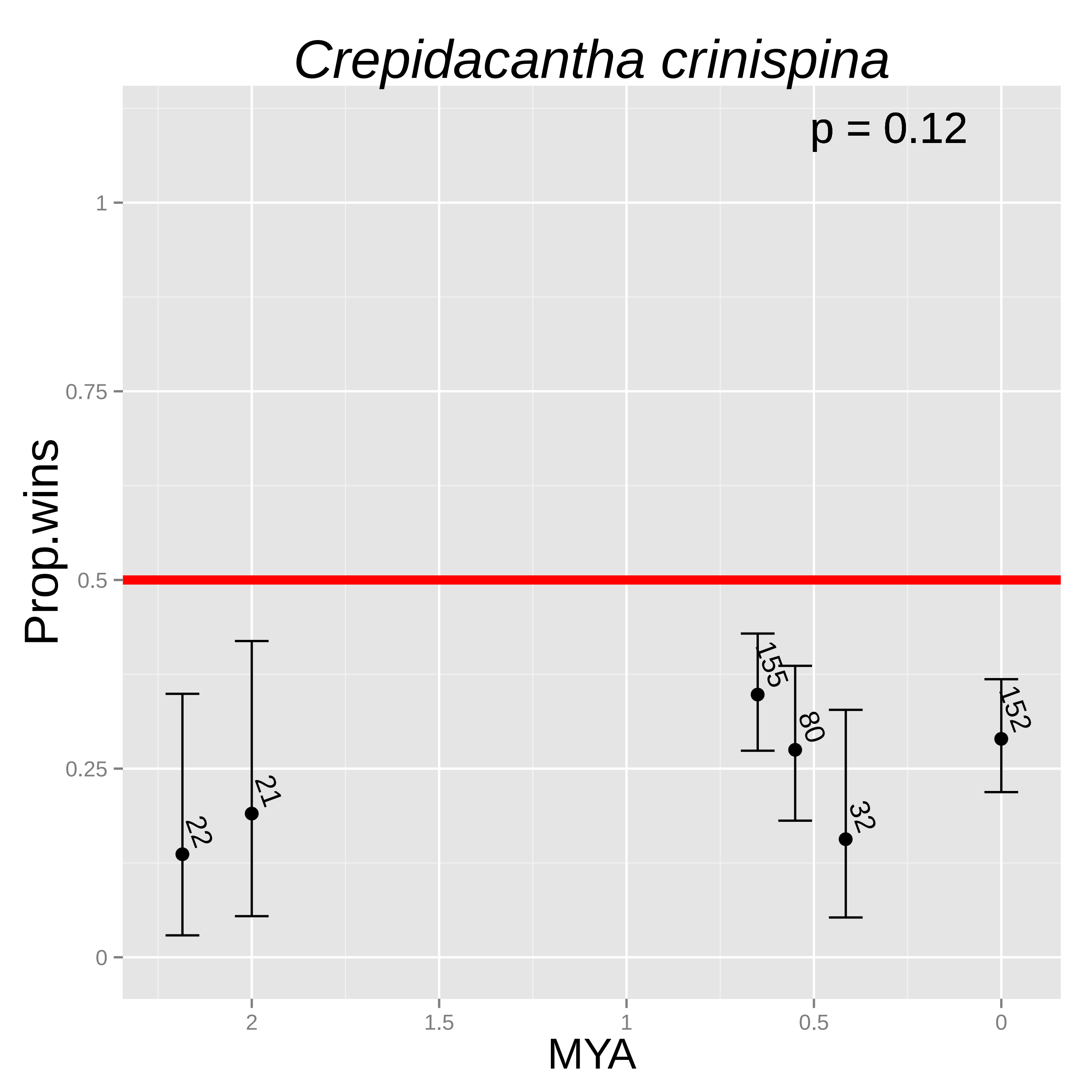
Crepidacantha crinispina
p = 0.12
Prop.wins
MYA
1
0.75
0.5
0.25
0
2
1.5
1
0.5
0
22
21
155
80
32
152

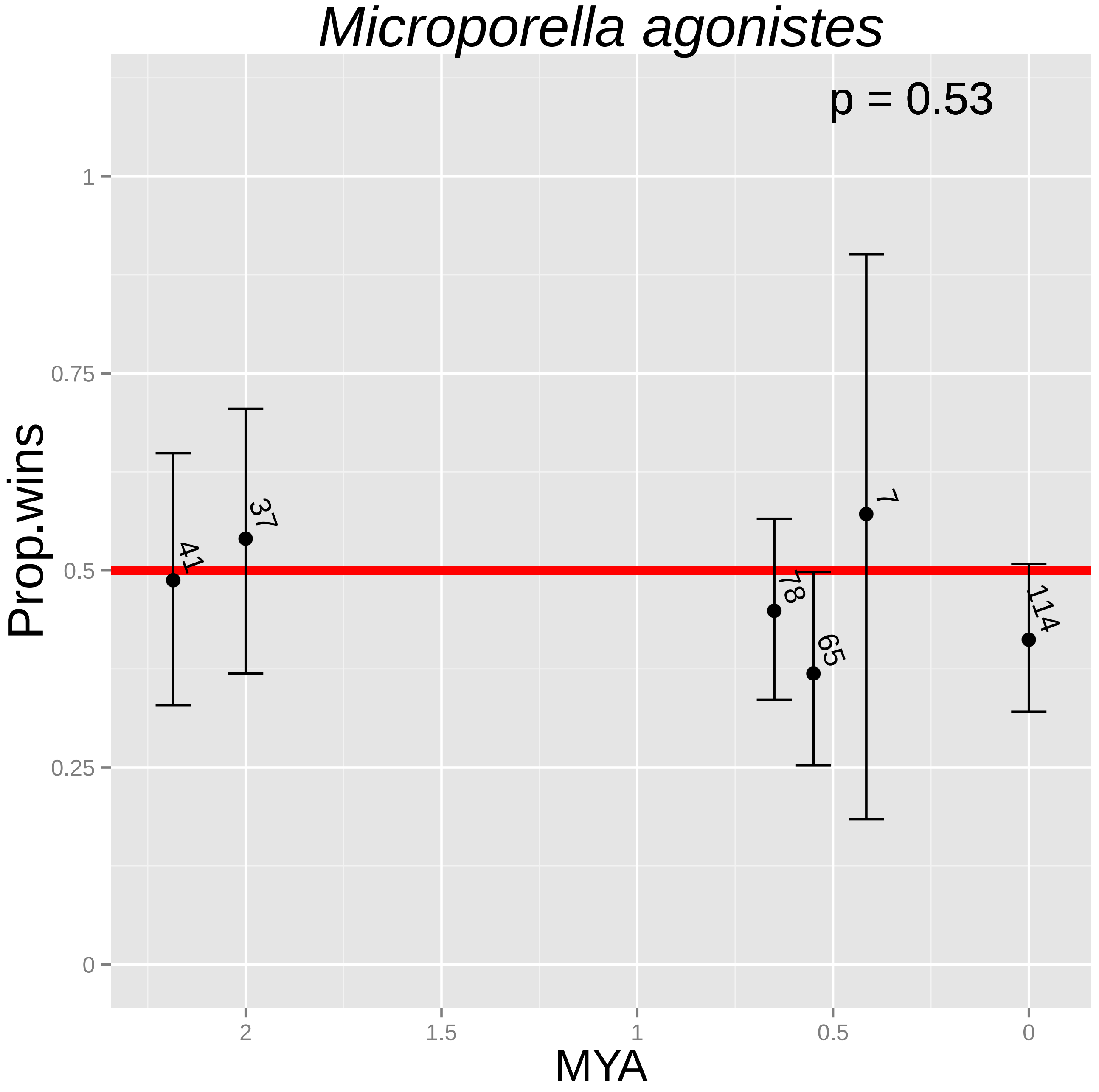
Microporella agonistes
p = 0.53
Prop.wins
MYA
1
0.75
0.5
0.25
0
2
1.5
1
0.5
0
41
37
78
65
7
114

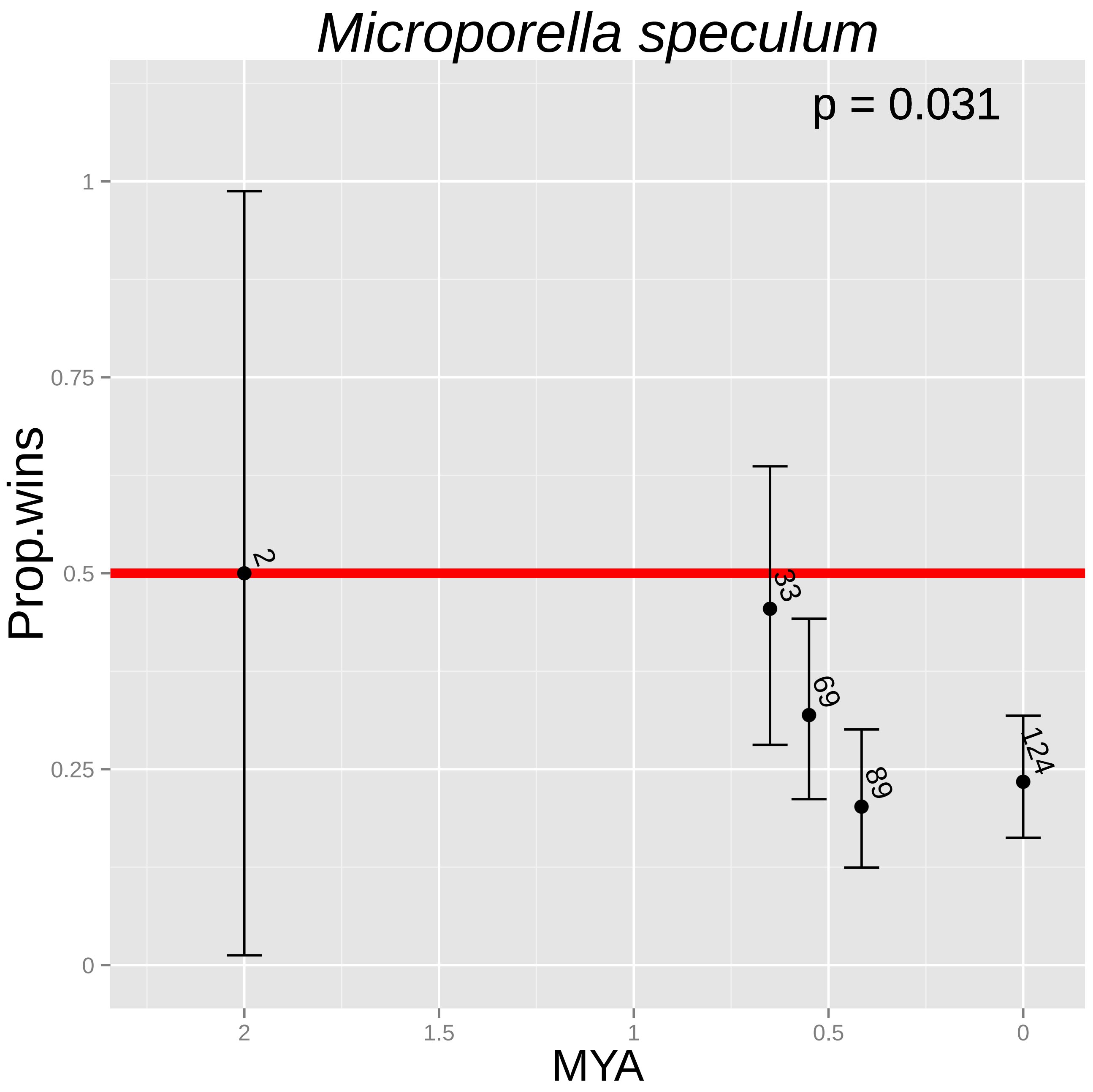
Microporella speculum
p = 0.031
Prop.wins
MYA
1
0.75
0.5
0.25
0
2
1.5
1
0.5
0
2
33
69
89
124

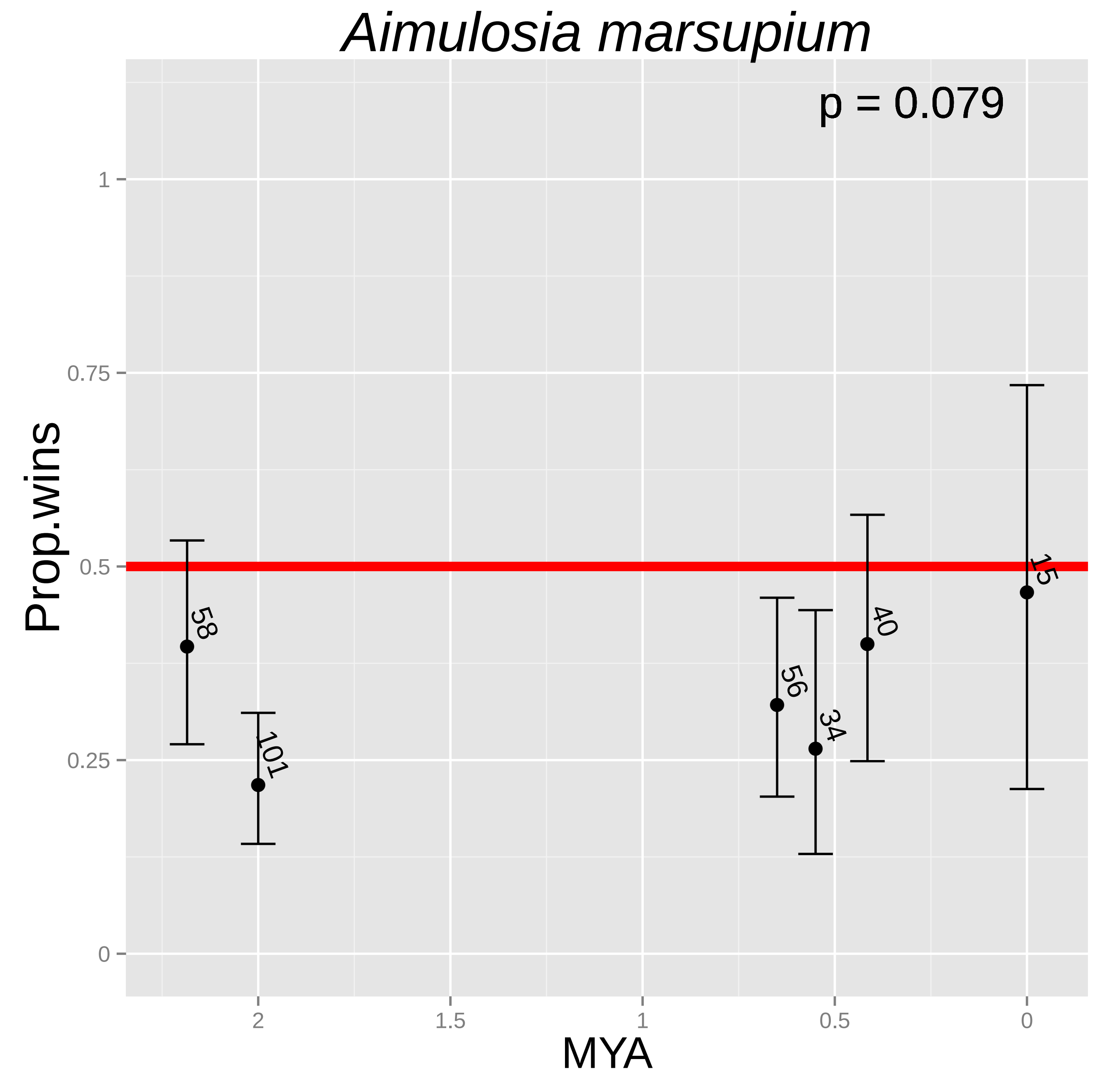
Aimulosia marsupium
p = 0.079
Prop.wins
MYA
1
0.75
0.5
0.25
0
2
1.5
1
0.5
0
58
101
56
34
40
15

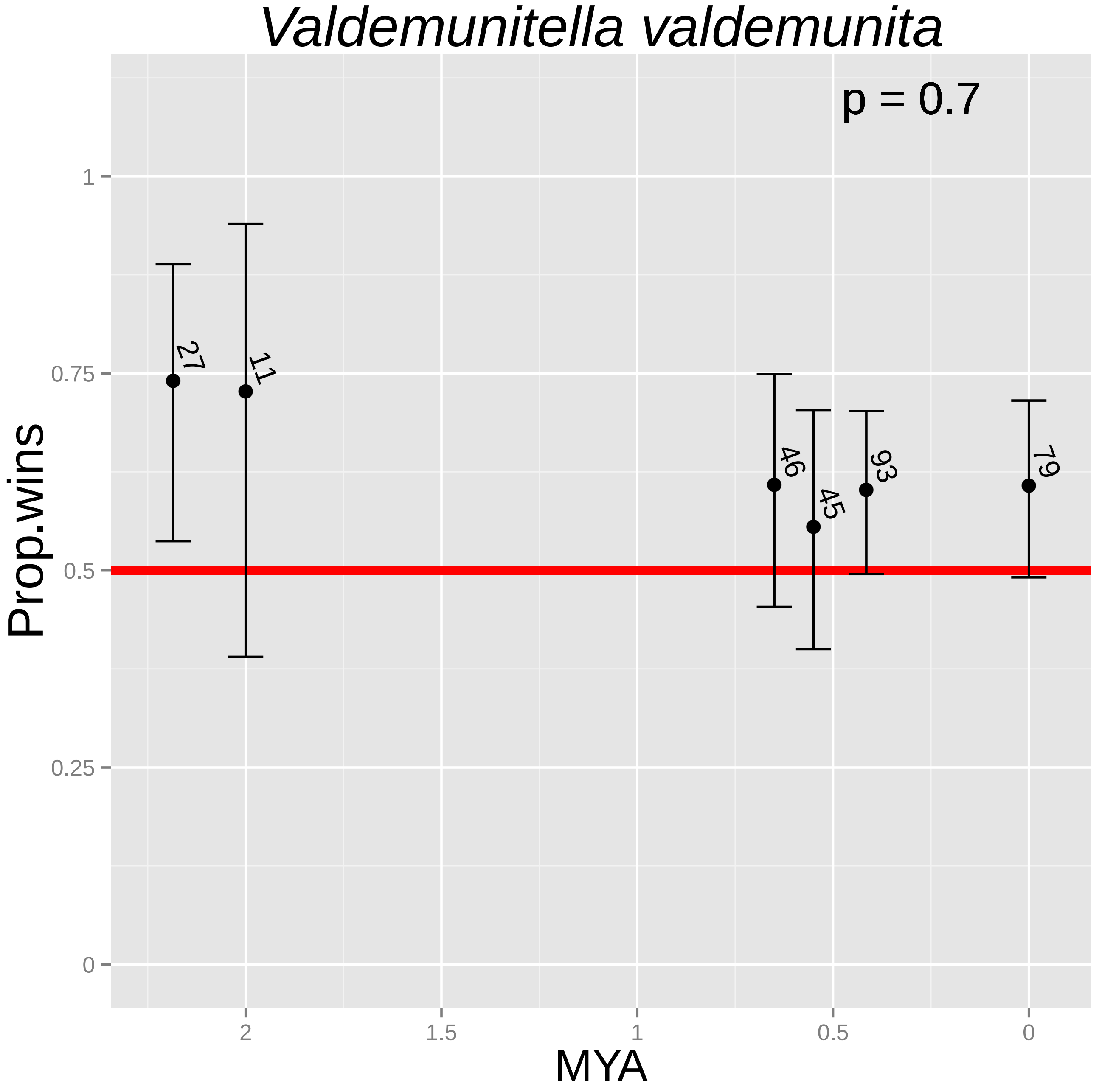
Valdemunitella valdemunita
p = 0.7
Prop.wins
MYA
1
0.75
0.5
0.25
0
2
1.5
1
0.5
0
27
11
46
45
93
79

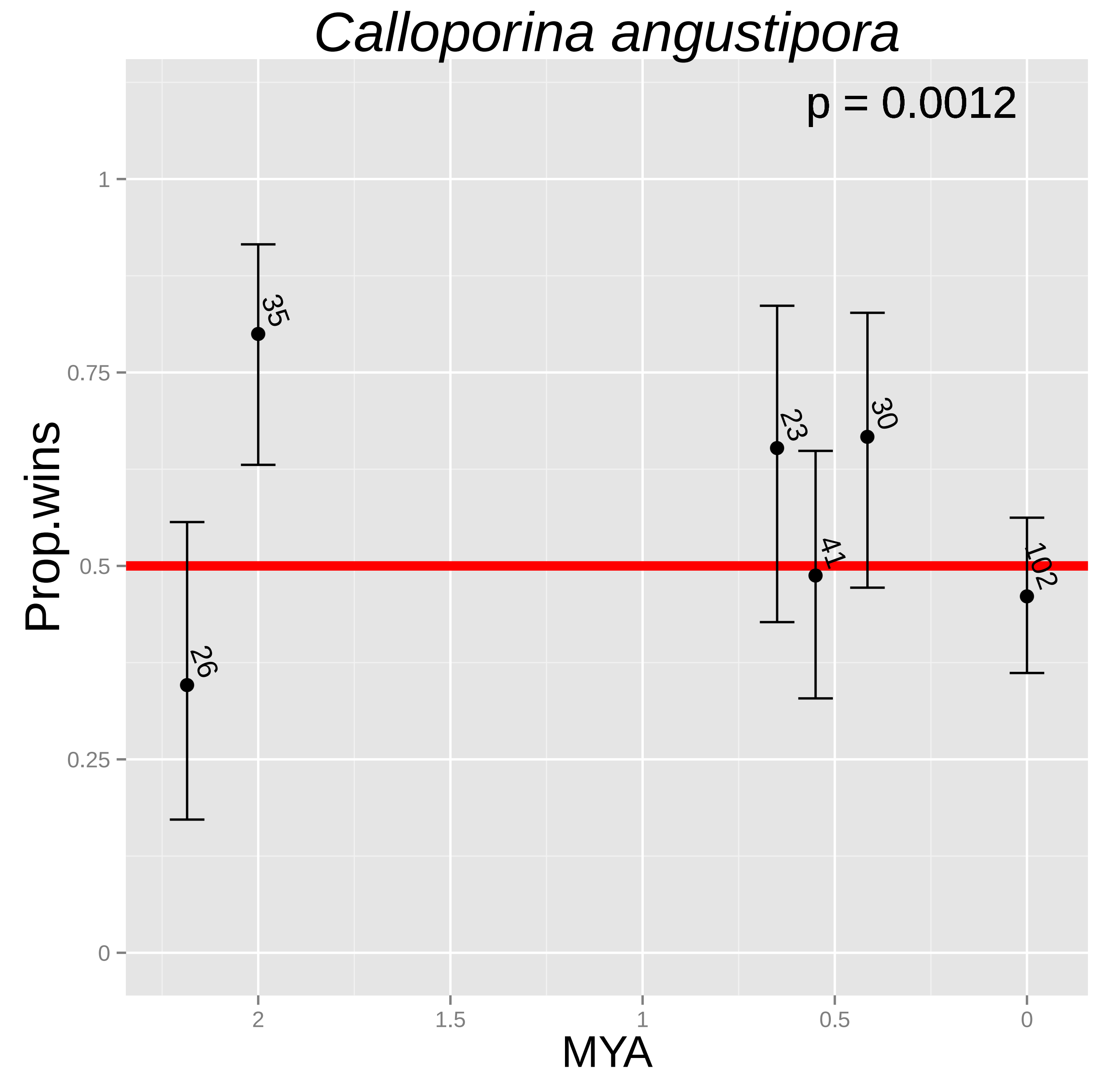

Calloporina angustipora
p = 0.0012
Prop.wins
MYA
1
0.75
0.5
0.25
0
2
1.5
1
0.5
0
35
26
23
41
30
102

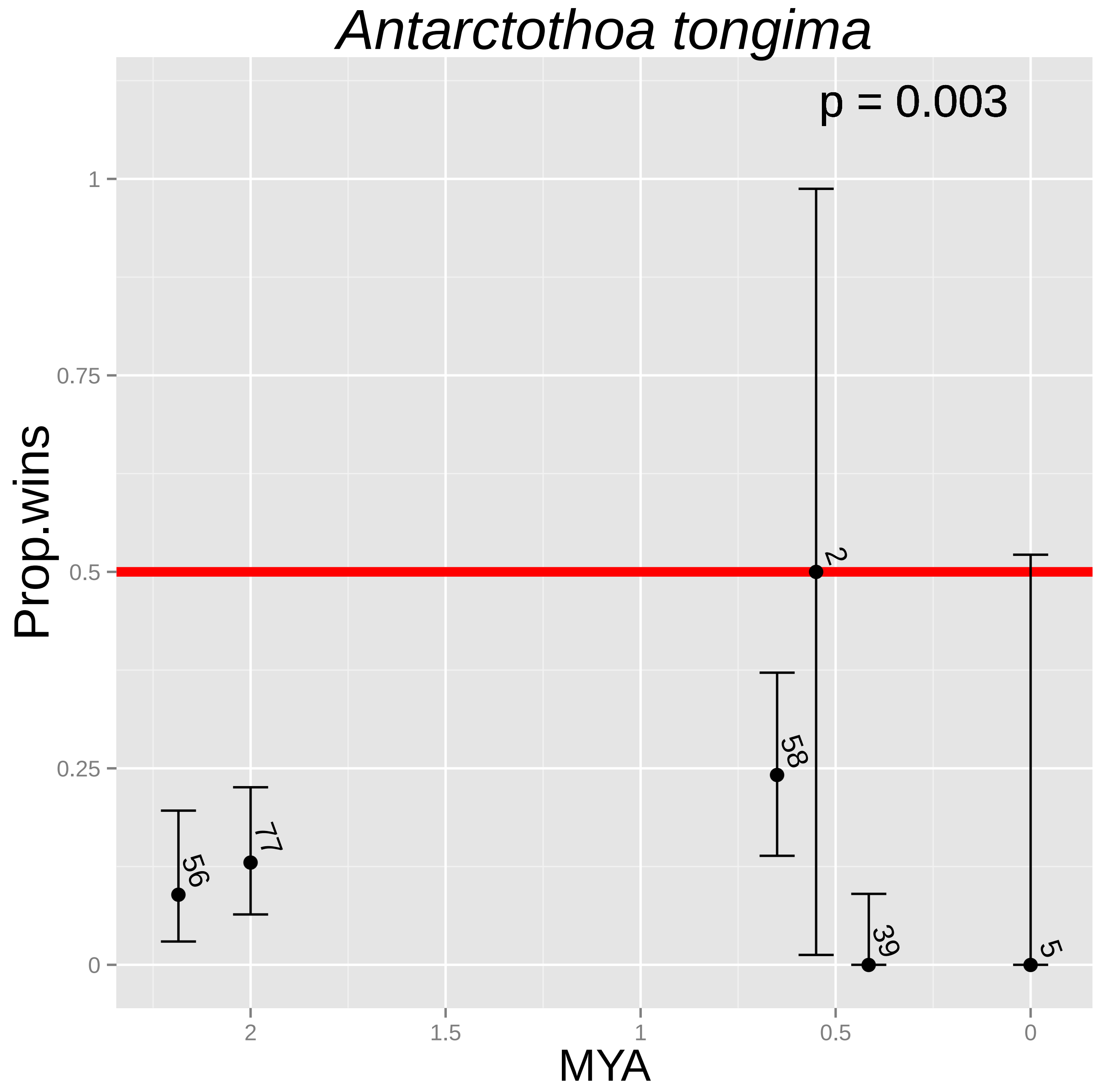
Antarctothoa tongima
p = 0.003
Prop.wins
MYA
1
0.75
0.5
0.25
0
2
1.5
1
0.5
0
56
77
58
2
39
5

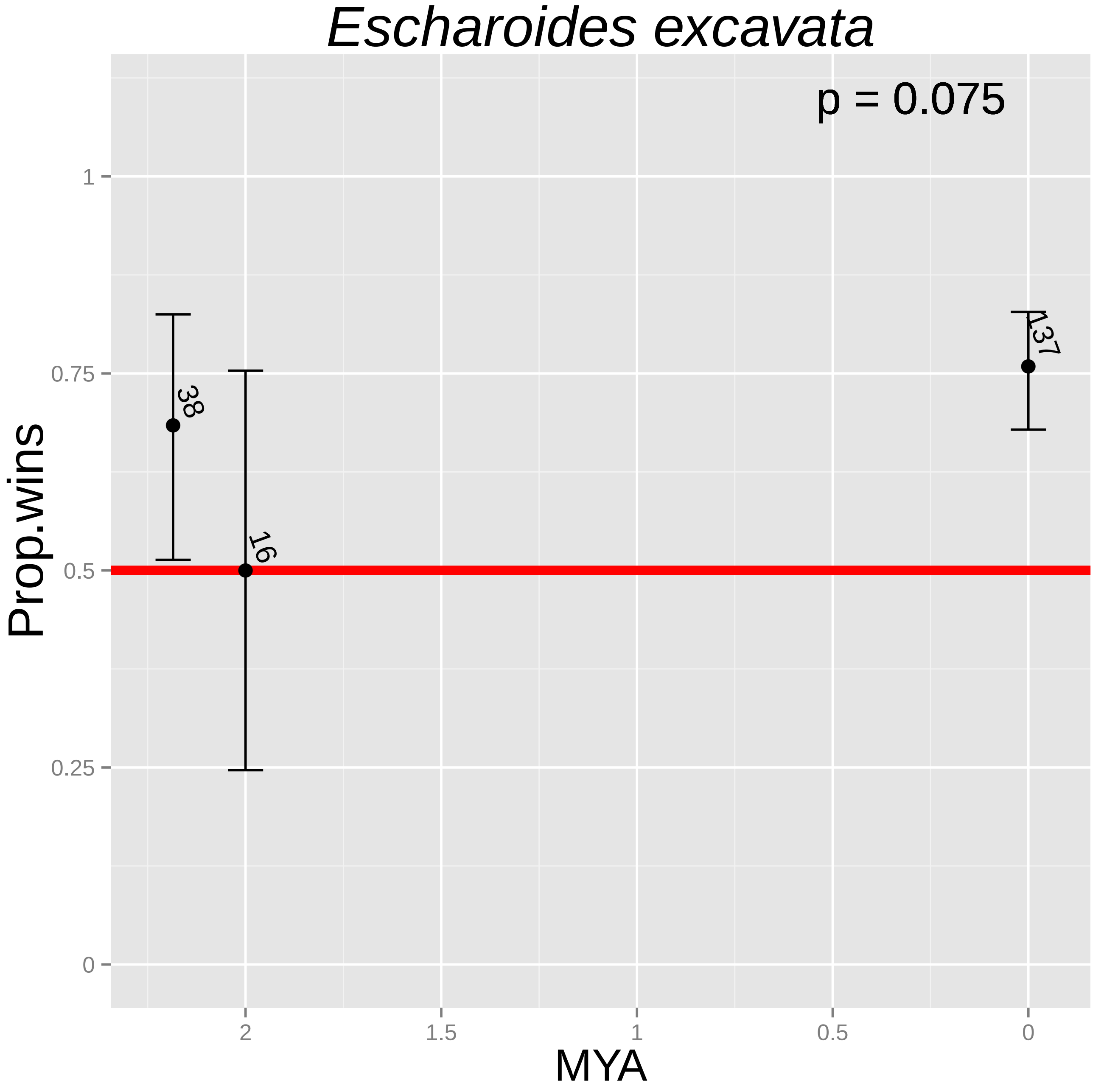

Escharoides excavata
p = 0.075
Prop.wins
MYA
1
0.75
0.5
0.25
0
2
1.5
1
0.5
0
38
16
137

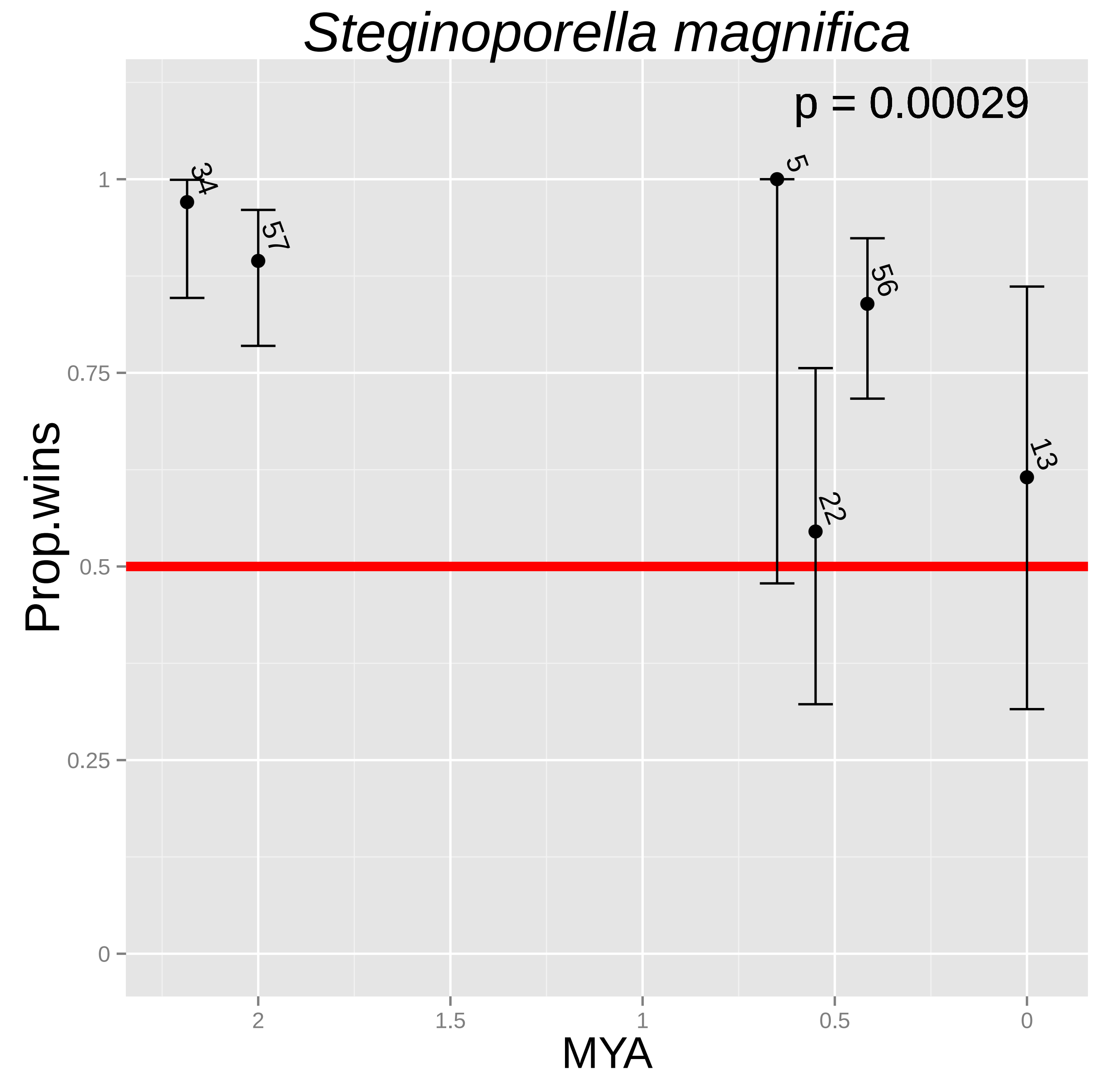

Steginoporella magnifica
p = 0.00029
Prop.wins
MYA
1
0.75
0.5
0.25
0
2
1.5
1
0.5
0
34
57
5
22
56
13

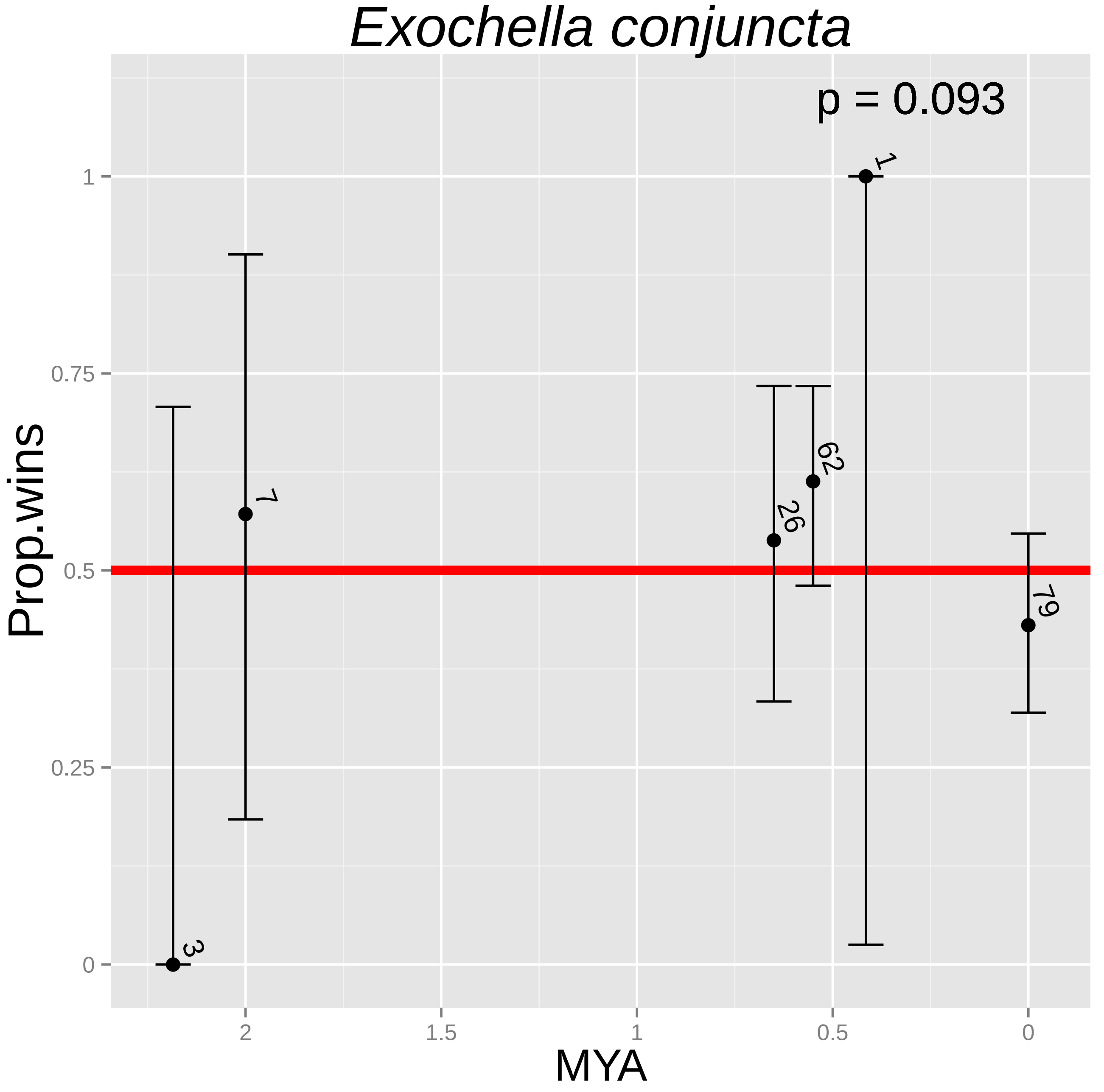
Exochella conjuncta
p = 0.093
Prop.wins
1
0.75
0.5
0.25
0
2
1.5
1
0.5
0
MYA
3
7
26
62
1
79

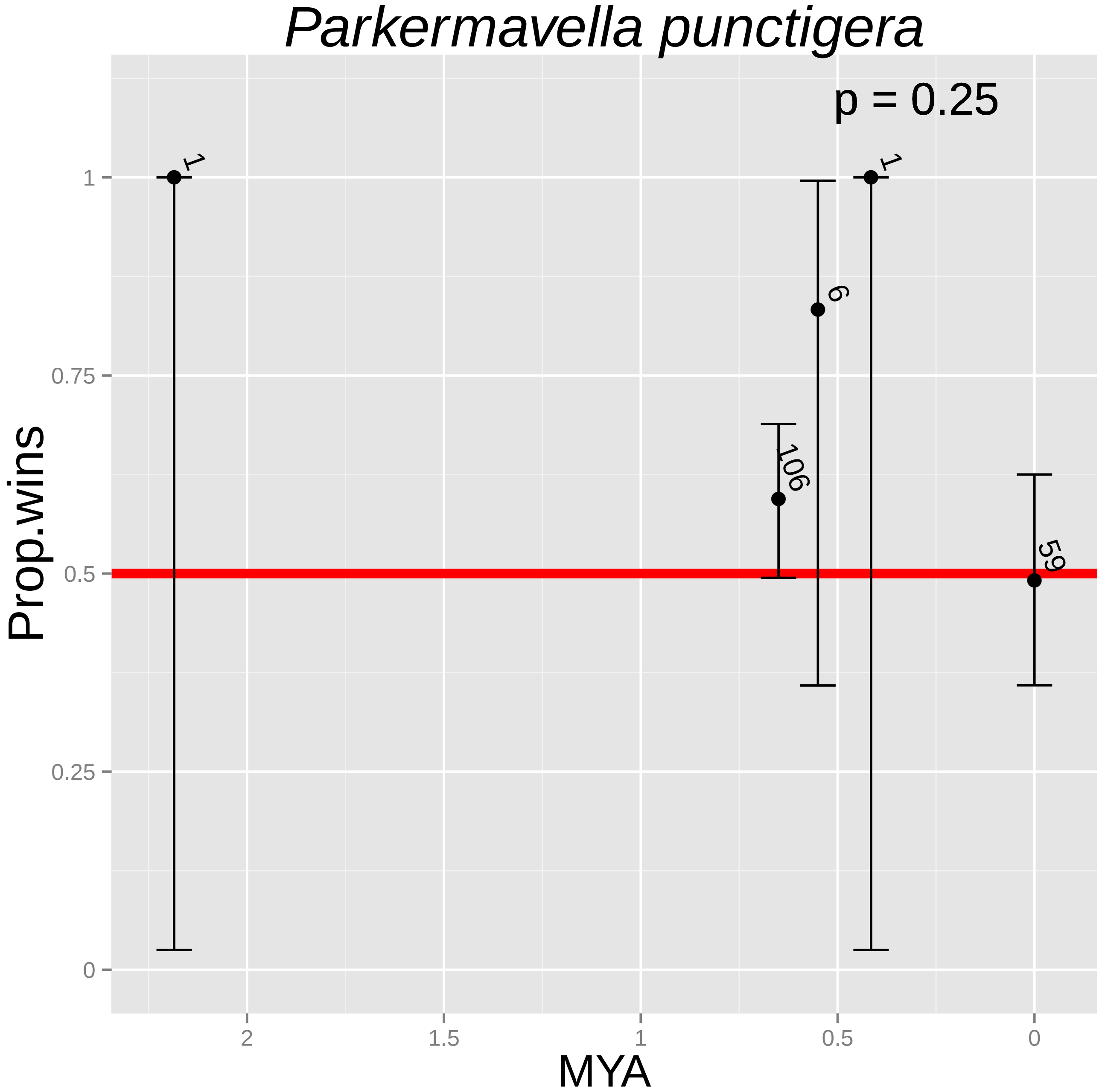

Parkermavella punctigera
p = 0.25
Prop.wins
MYA
1
0.75
0.5
0.25
0
2
1.5
1
0.5
0
1
9
106
1
59

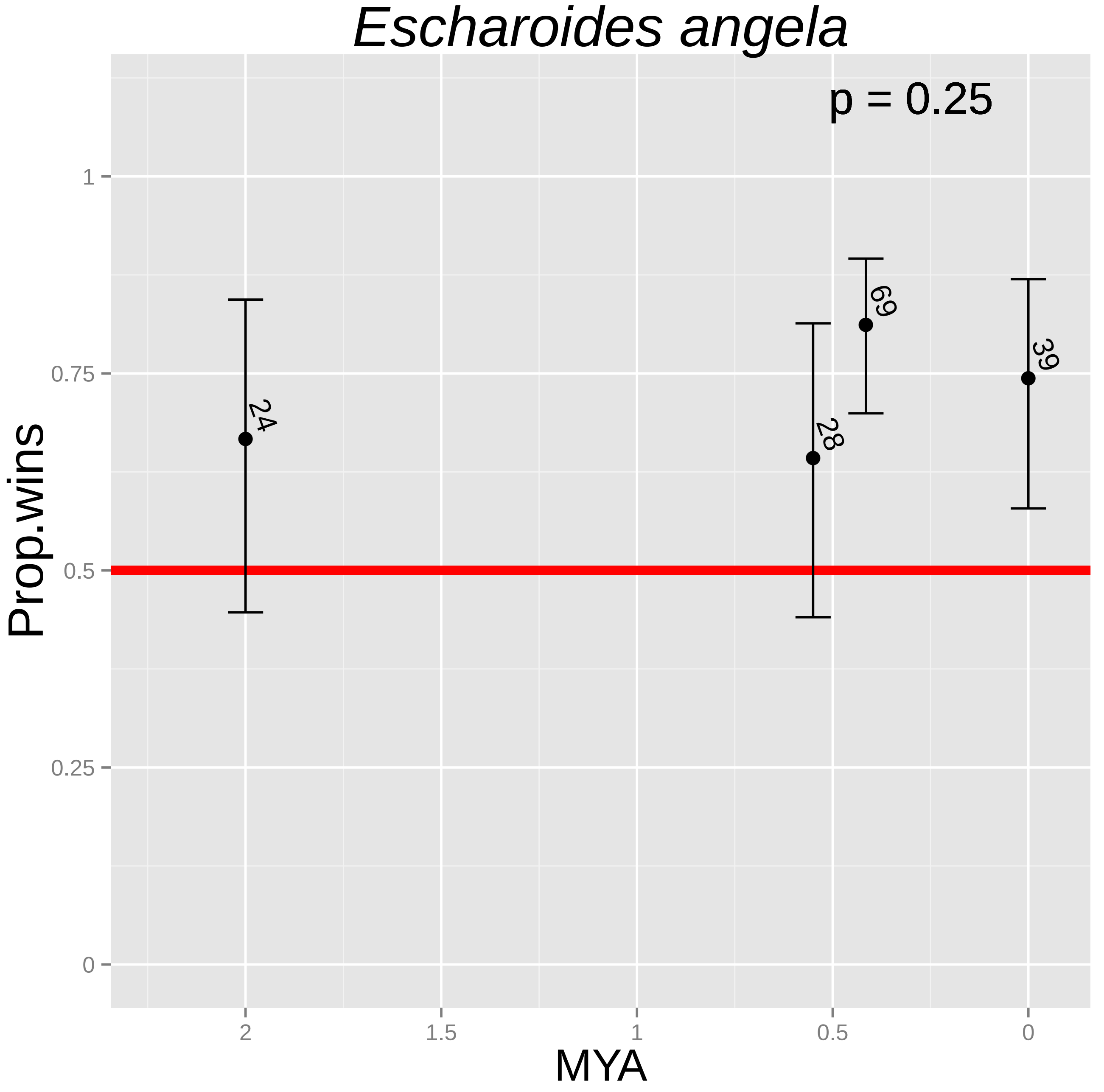

Escharoides angela
p = 0.25
Prop.wins
MYA
1
0.75
0.5
0.25
0
2
1.5
1
0.5
0
24
28
69
39

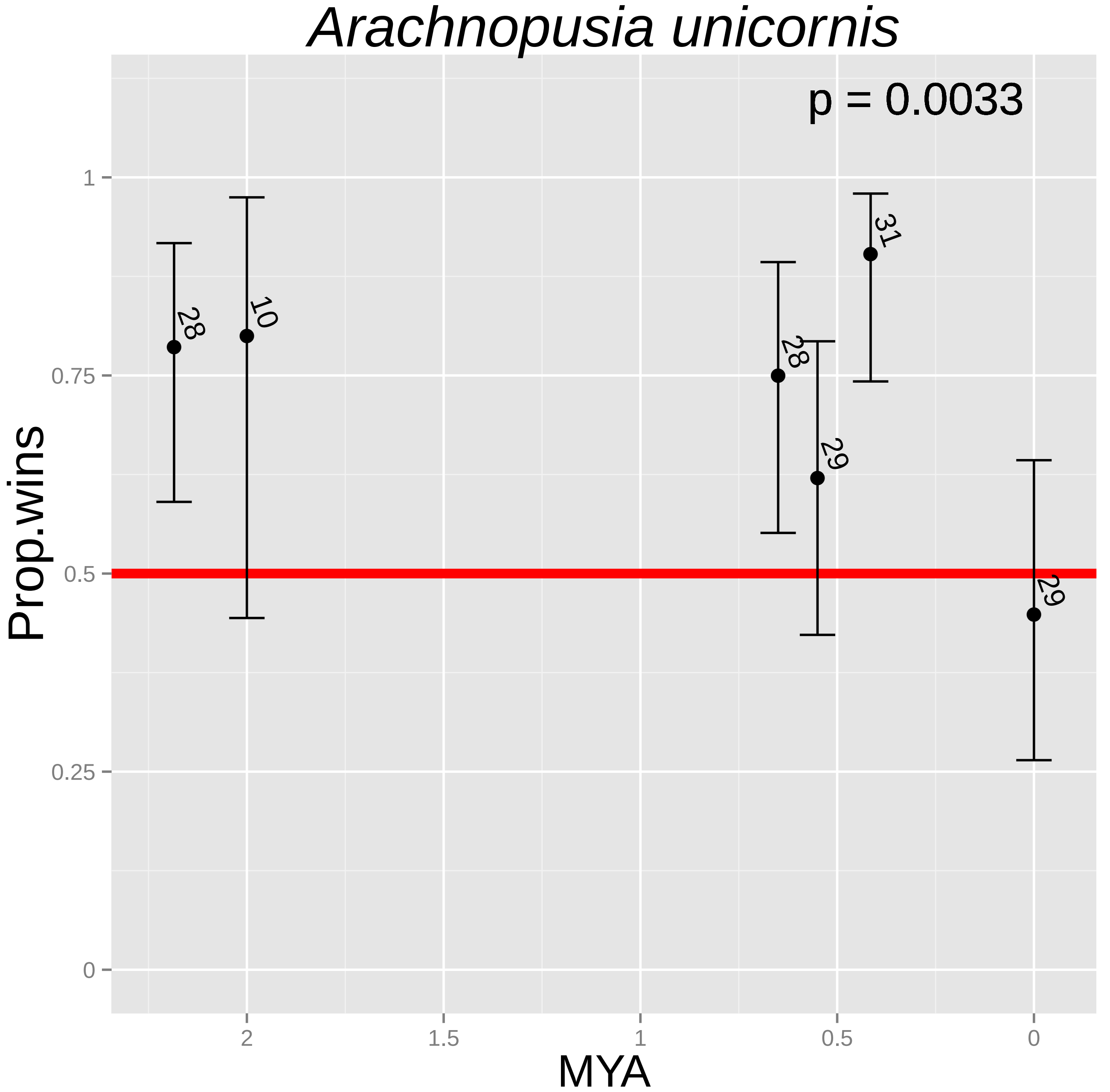

Arachnopusia unicornis
p = 0.0033
Prop.wins
MYA
1
0.75
0.5
0.25
0
2
1.5
1
0.5
0
28
10
28
29
31
29

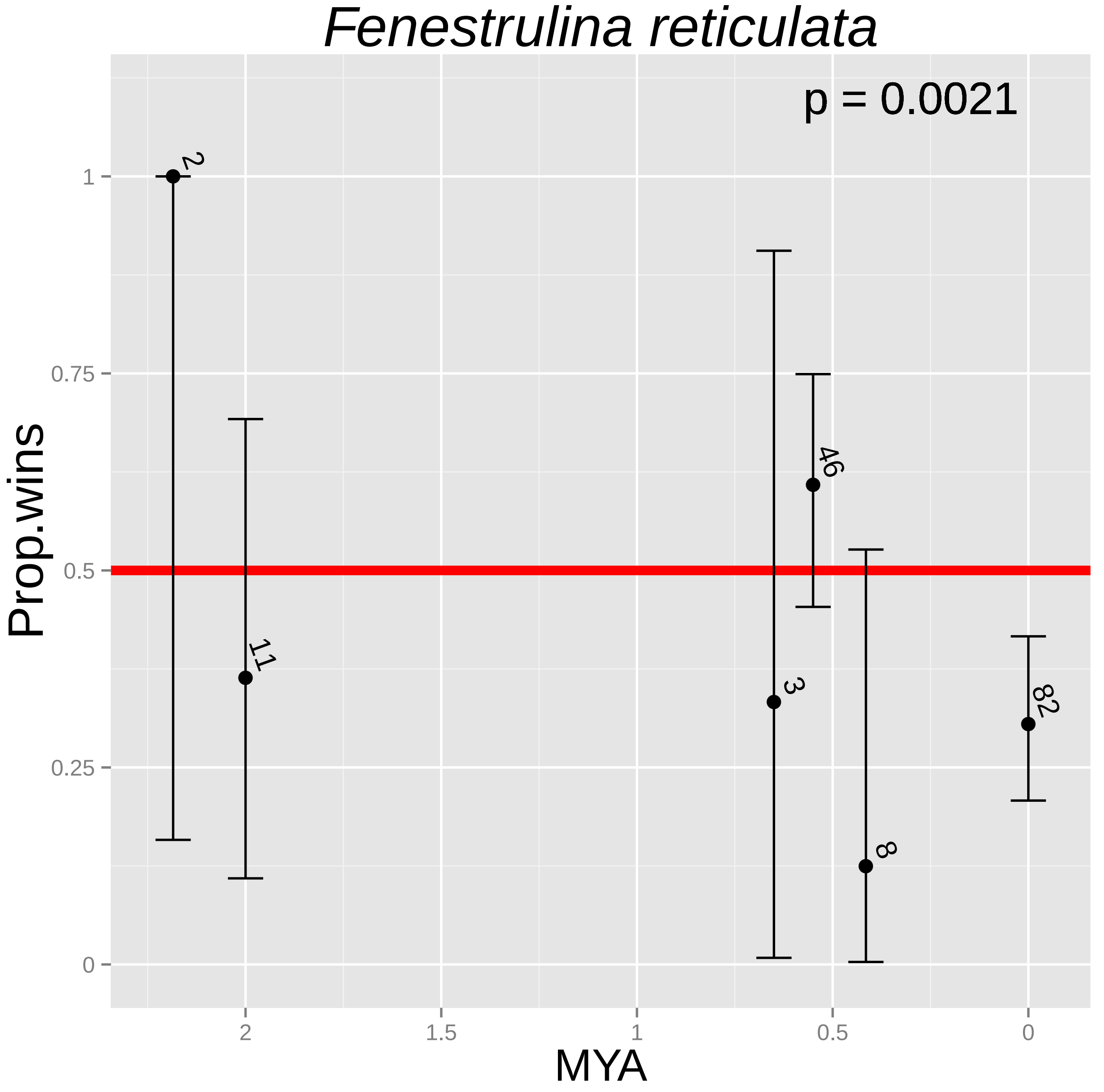

Fenestrulina reticulata
p = 0.0021
Prop.wins
MYA
1
0.75
0.5
0.25
0
2
1.5
1
0.5
0
2
11
46
3
8
82

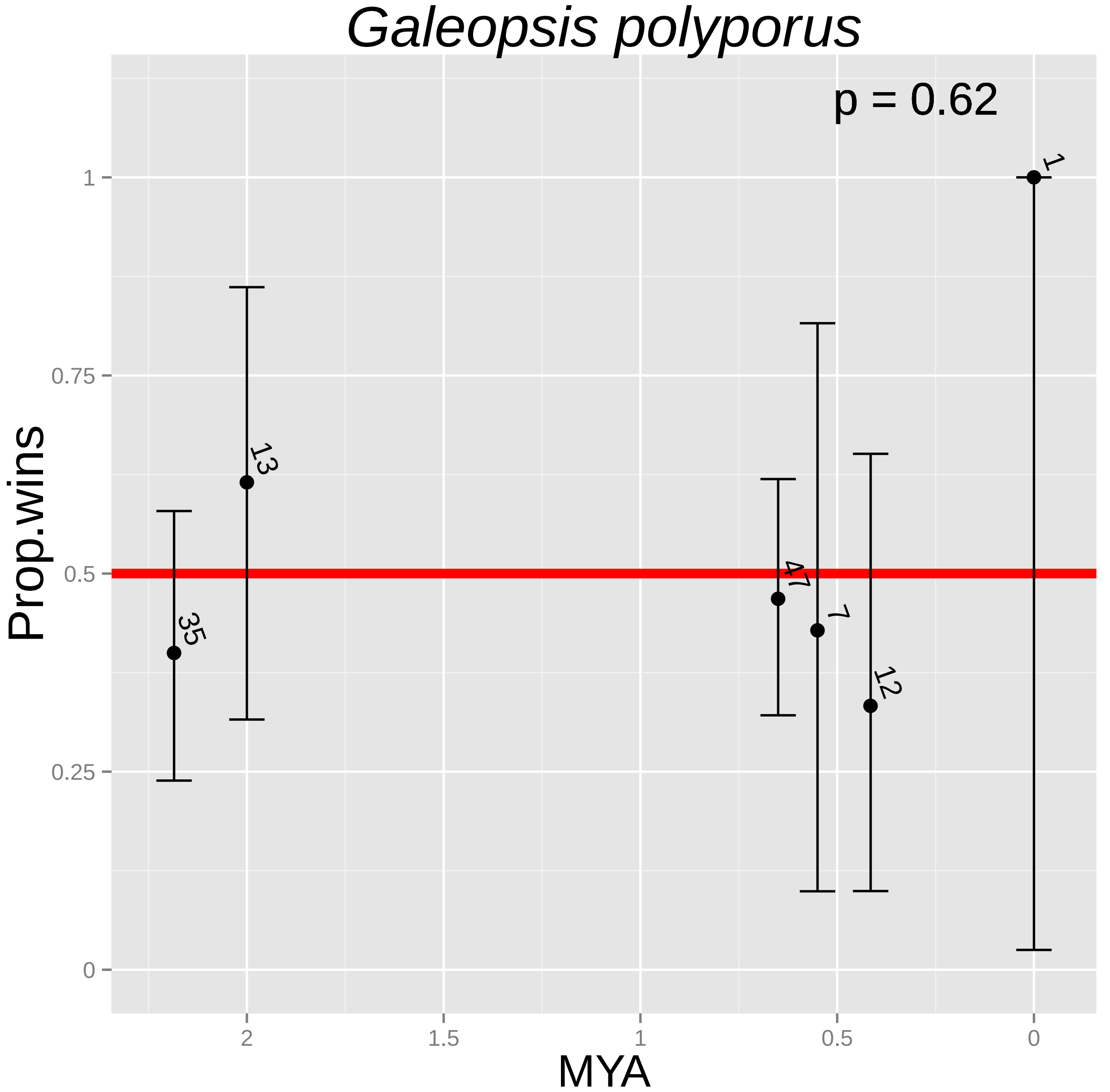
Galeopsis polyporus
p = 0.62
Prop.wins
MYA
1
0.75
0.5
0.25
0
2
1.5
1
0.5
0
35
13
47
7
12
1

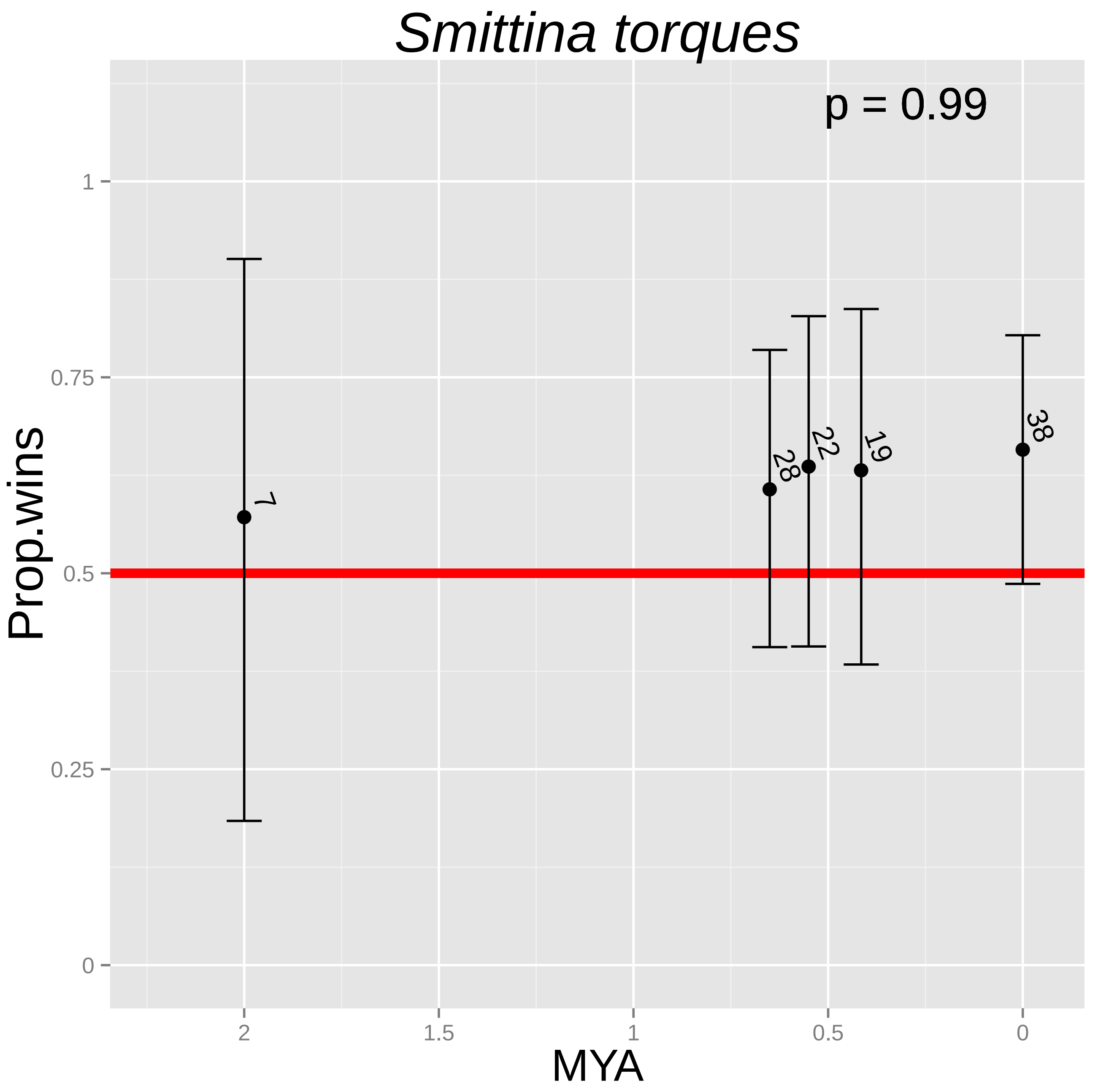

Smittina torques
p = 0.99
Prop.wins
MYA
1
0.75
0.5
0.25
0
2
1.5
1
0.5
0
7
28
22
19
38

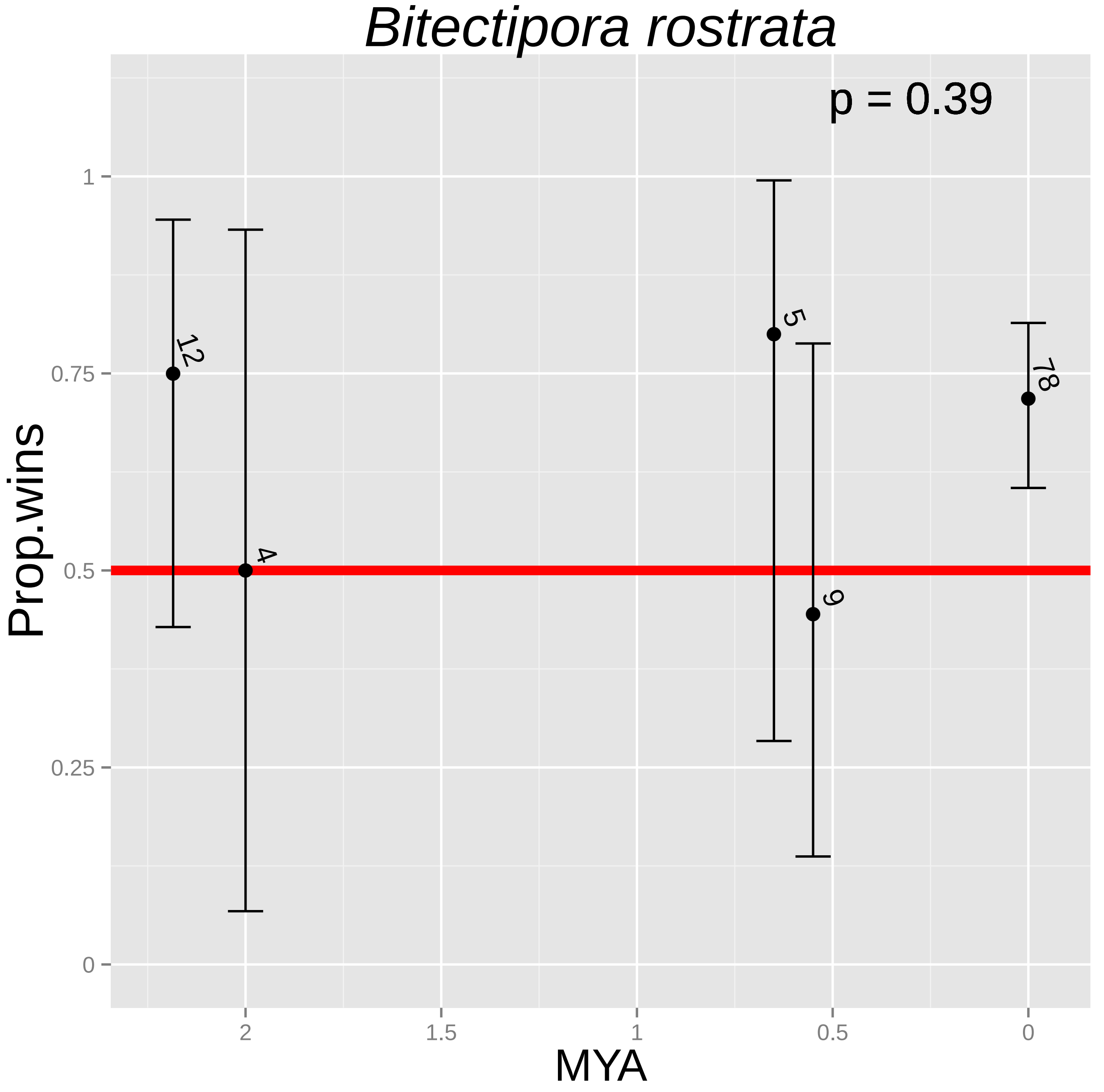

Bitectipora rostrata
p = 0.39
Prop.wins
MYA
0
0.25
0.5
0.75
1
2
1.5
1
0.5
0
12
4
5
9
78

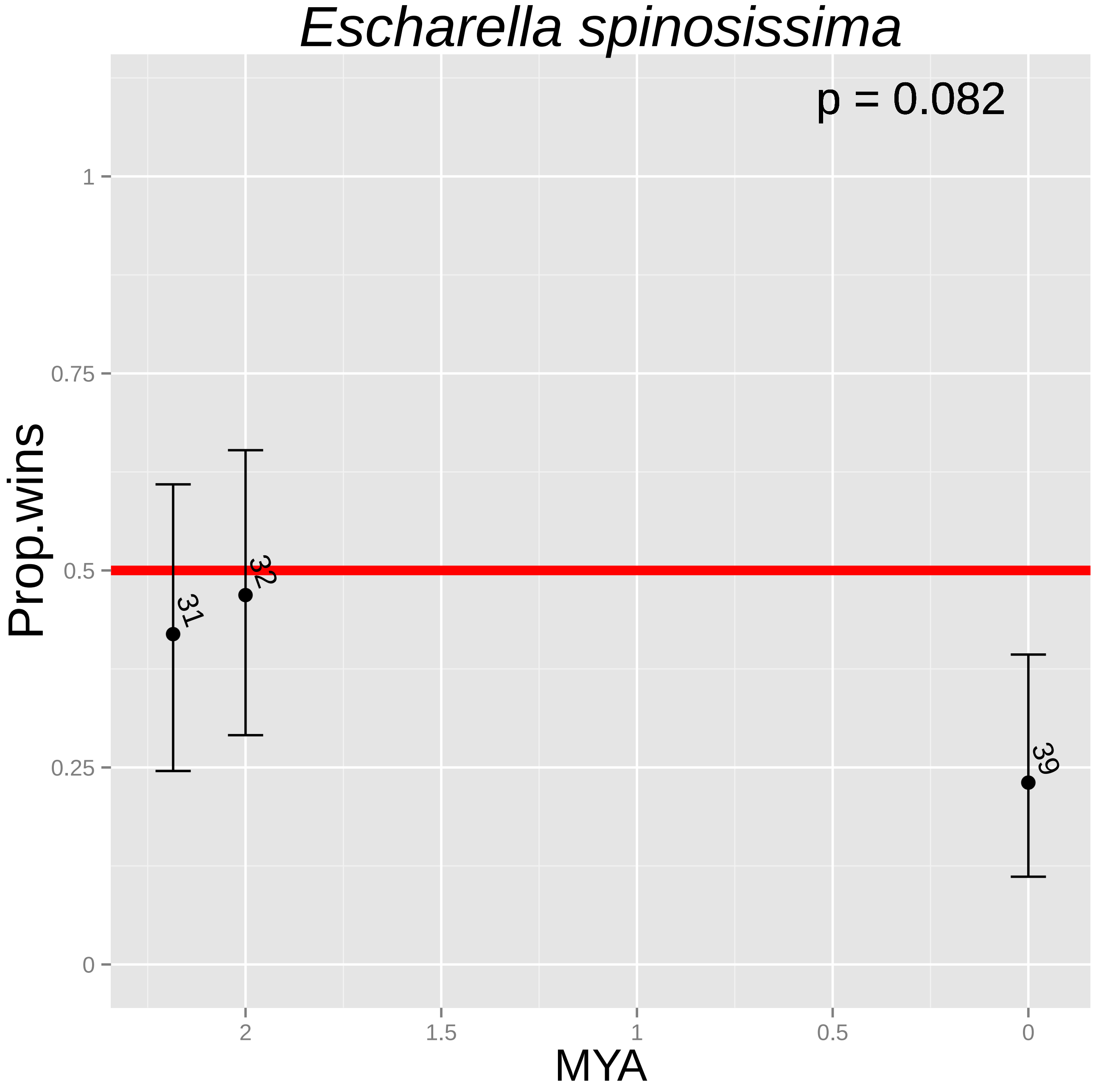

Escharella spinosissima
p = 0.082
Prop.wins
MYA
1
0.75
0.5
0.25
0
2
1.5
1
0.5
0
31
32
39

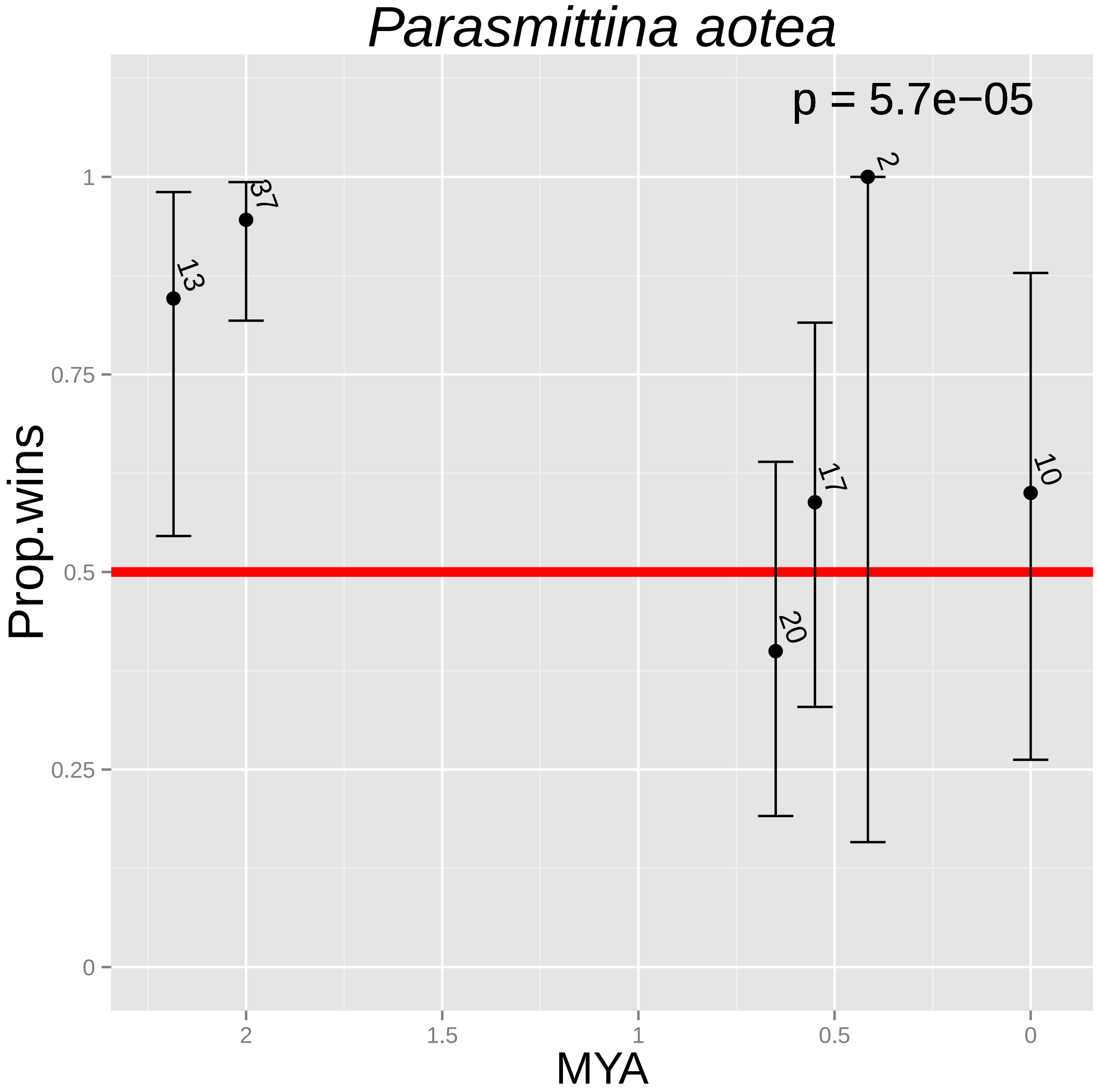
Parasmittina aotea
p = 5.7e−05
Prop.wins
MYA
1
0.75
0.5
0.25
0
2
1.5
1
0.5
0
13
37
2
17
10
20

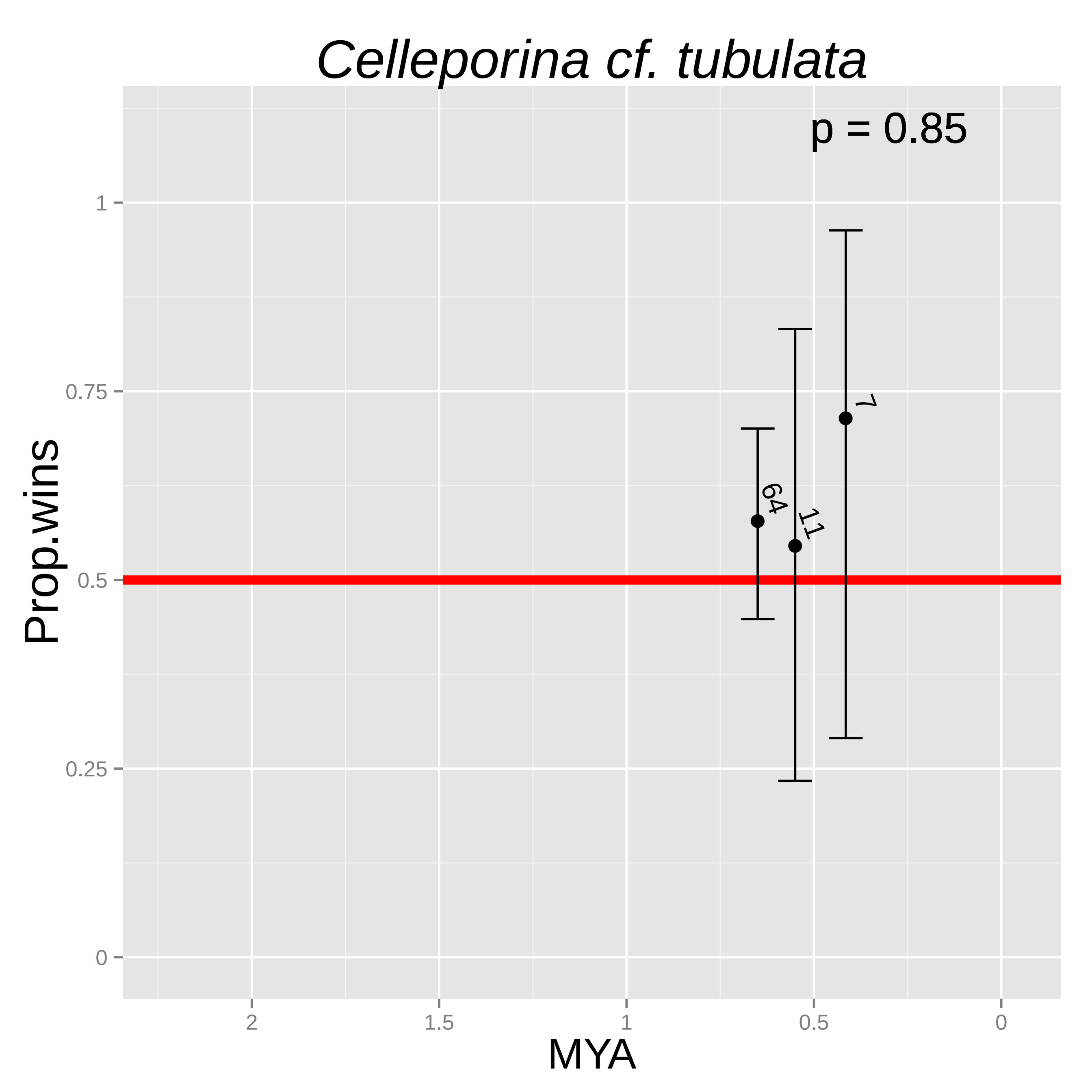
Celleporina cf. tubulata
p = 0.85
Prop.wins
1
0.75
0.5
0.25
0
64
11
7
2
1.5
1
0.5
0
MYA

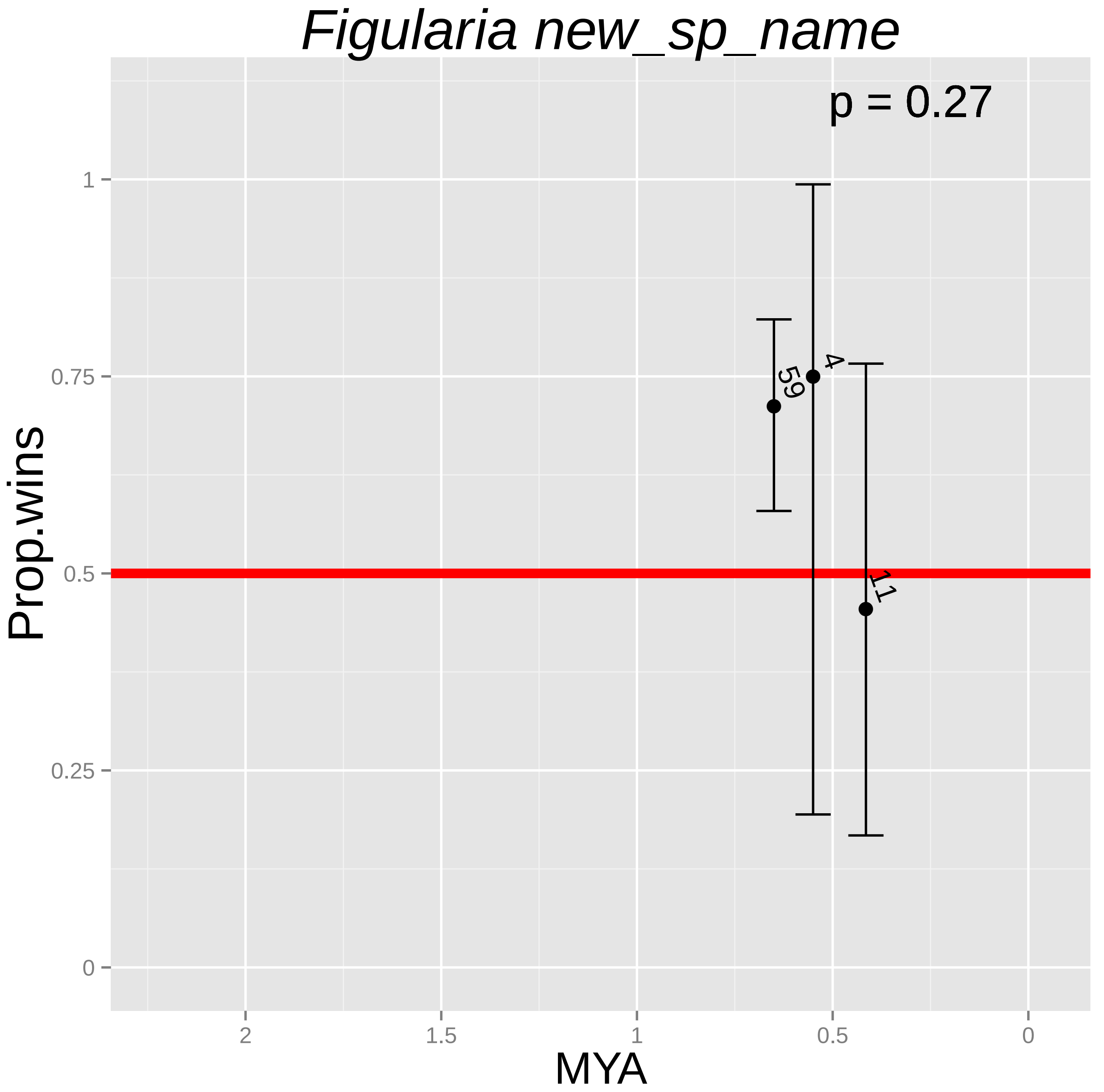

Figularia new_sp_name
p = 0.27
Prop.wins
MYA
1
0.75
0.5
0.25
0
2
1.5
1
0.5
0
4
59
11

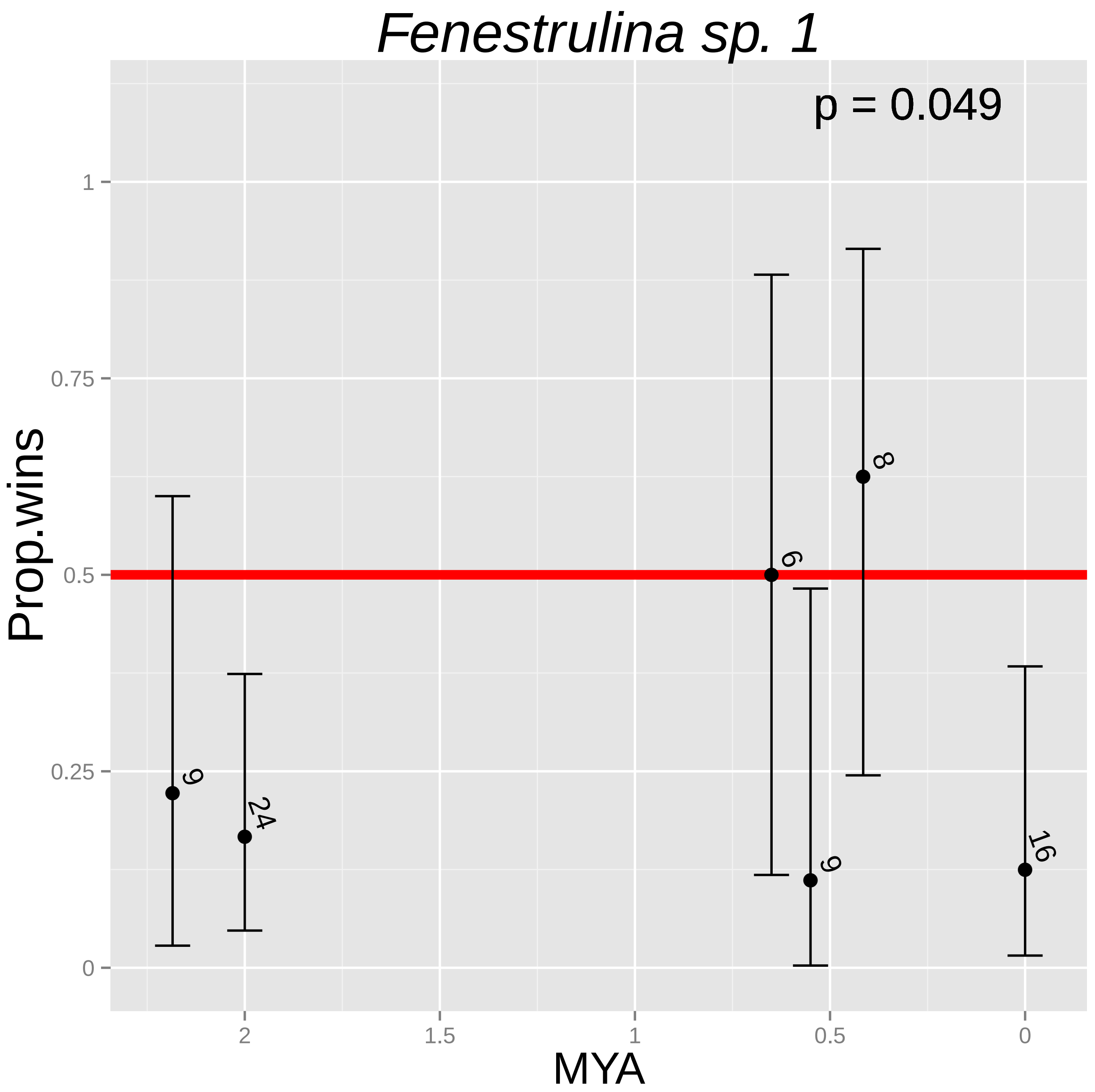

Fenestrulina sp. 1
p = 0.049
Prop.wins
1
0.75
0.5
0.25
0
2
1.5
1
0.5
0
MYA
9
24
9
8
9
16

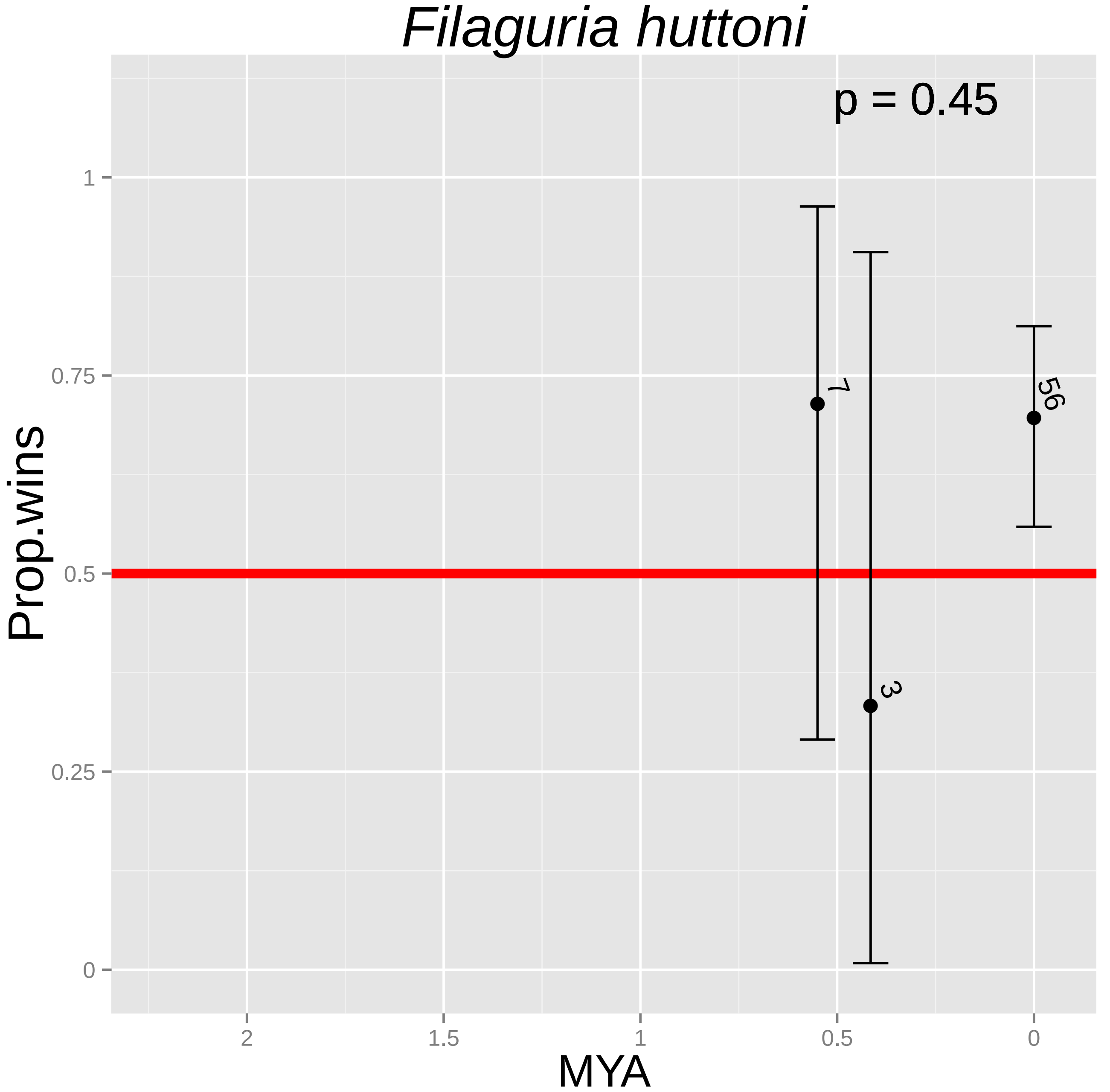
Filaguria huttoni
p = 0.45
Prop.wins
MYA
1
0.75
0.5
0.25
0
2
1.5
1
0.5
0
7
3
56

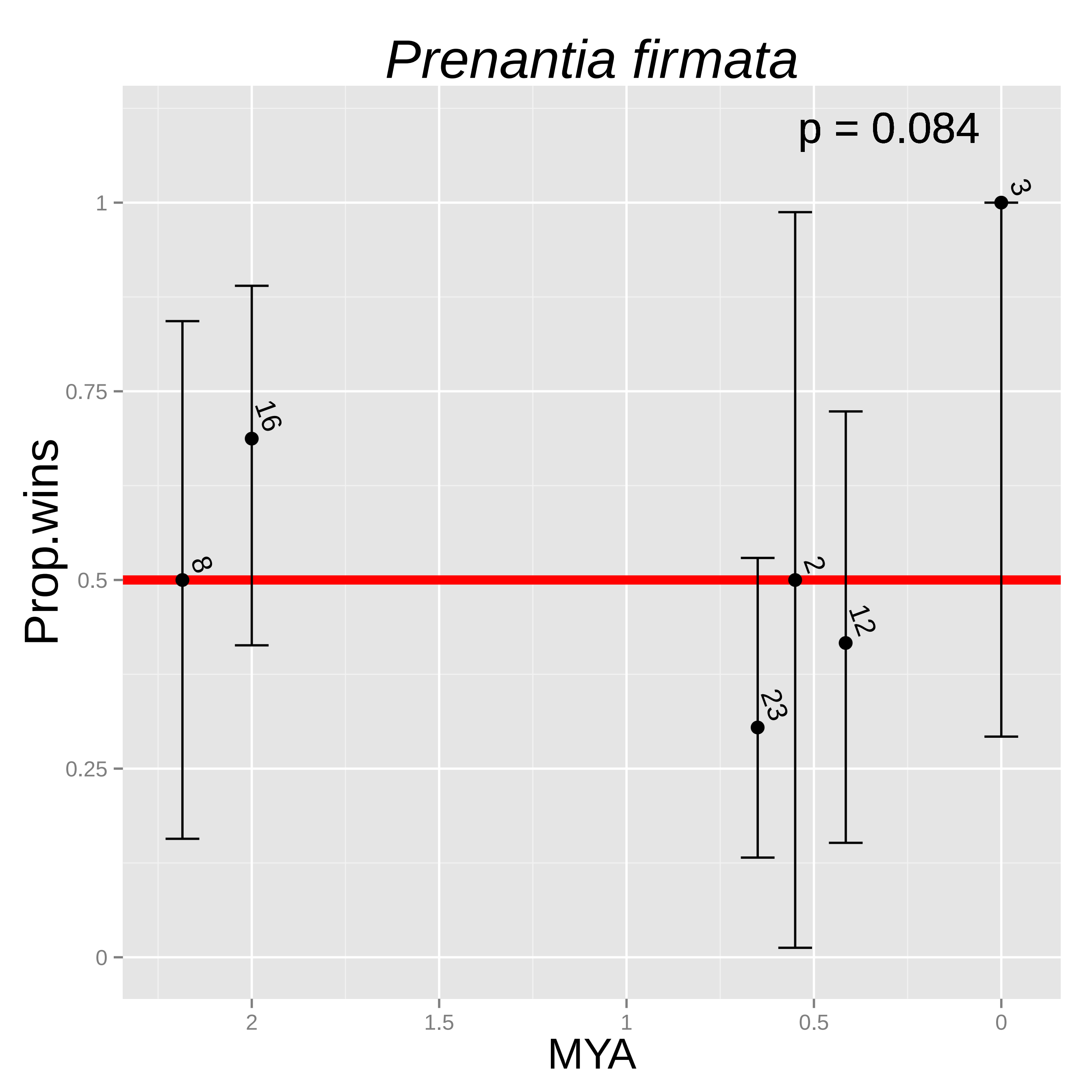

Prenantia firmata
p = 0.084
Prop.wins
1
0.75
0.5
0.25
0
3
16
8
2
12
23
2
1.5
1
0.5
0
MYA

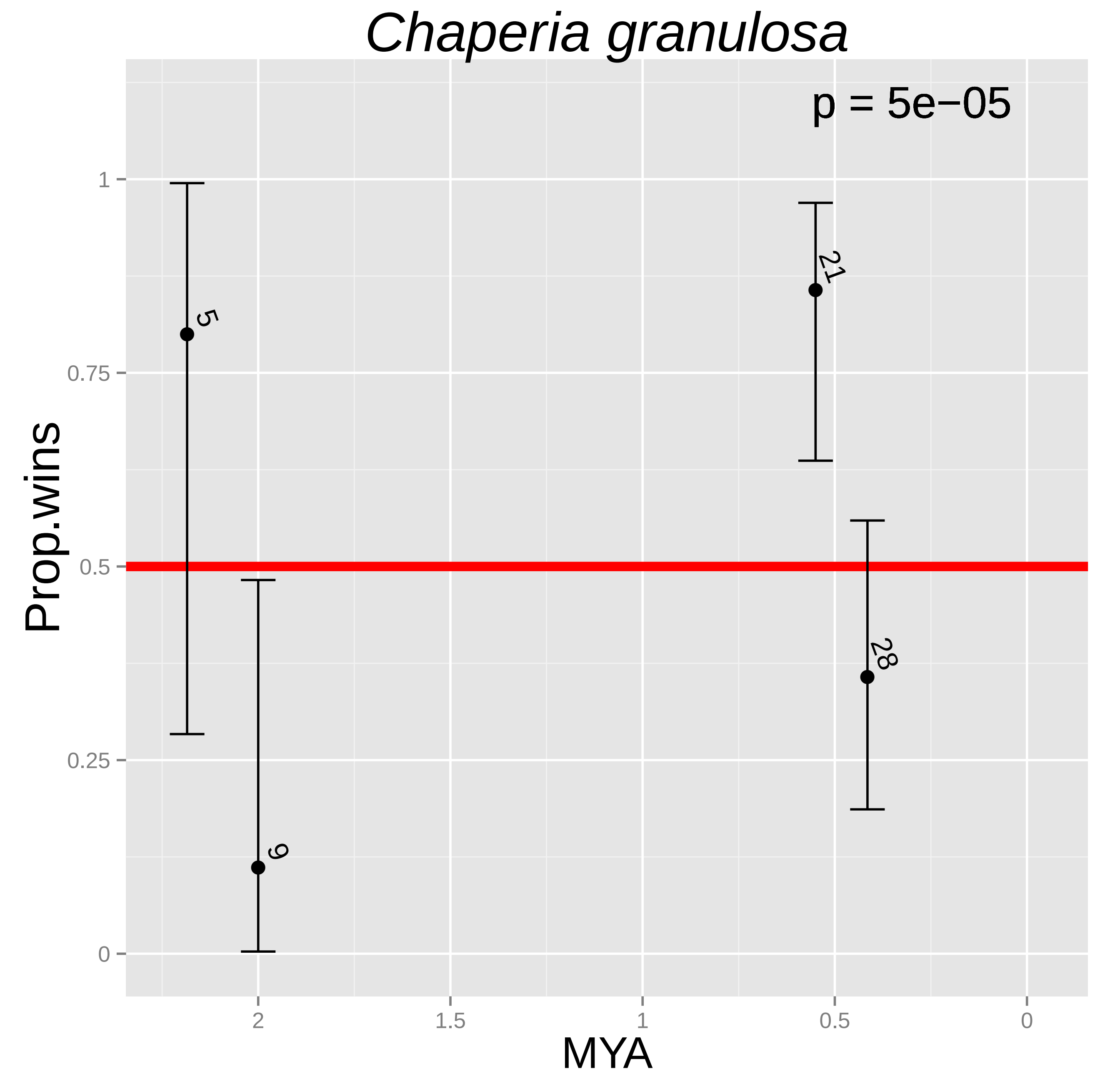

Chaperia granulosa
p = 5e−05
Prop.wins
1
0.75
0.5
0.25
0
5
9
21
28
2
1.5
1
0.5
0
MYA

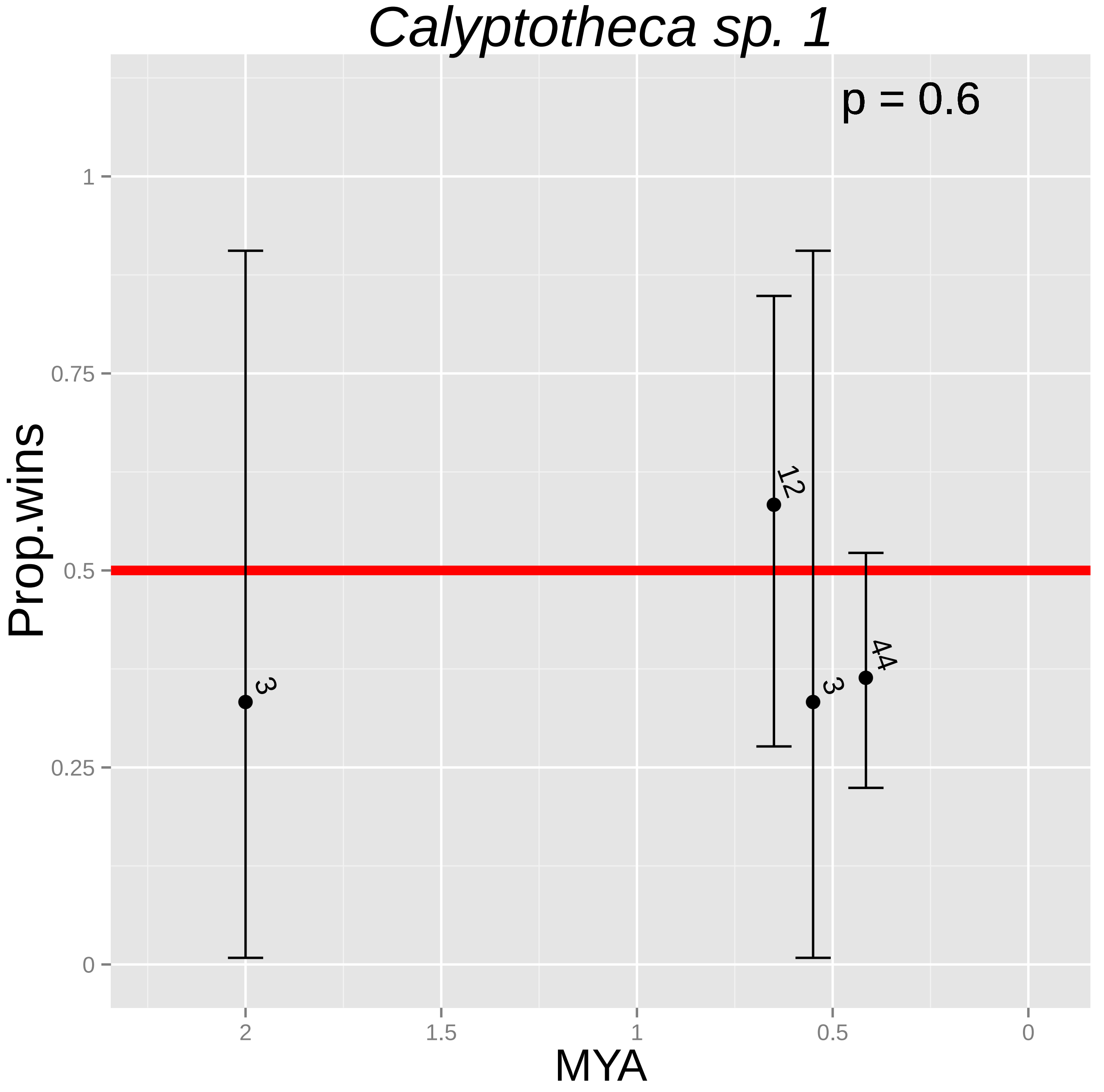

Calyptotheca sp. 1
p = 0.6
Prop.wins
1
0.75
0.5
0.25
0
12
3
44
3
2
1.5
1
0.5
0
MYA

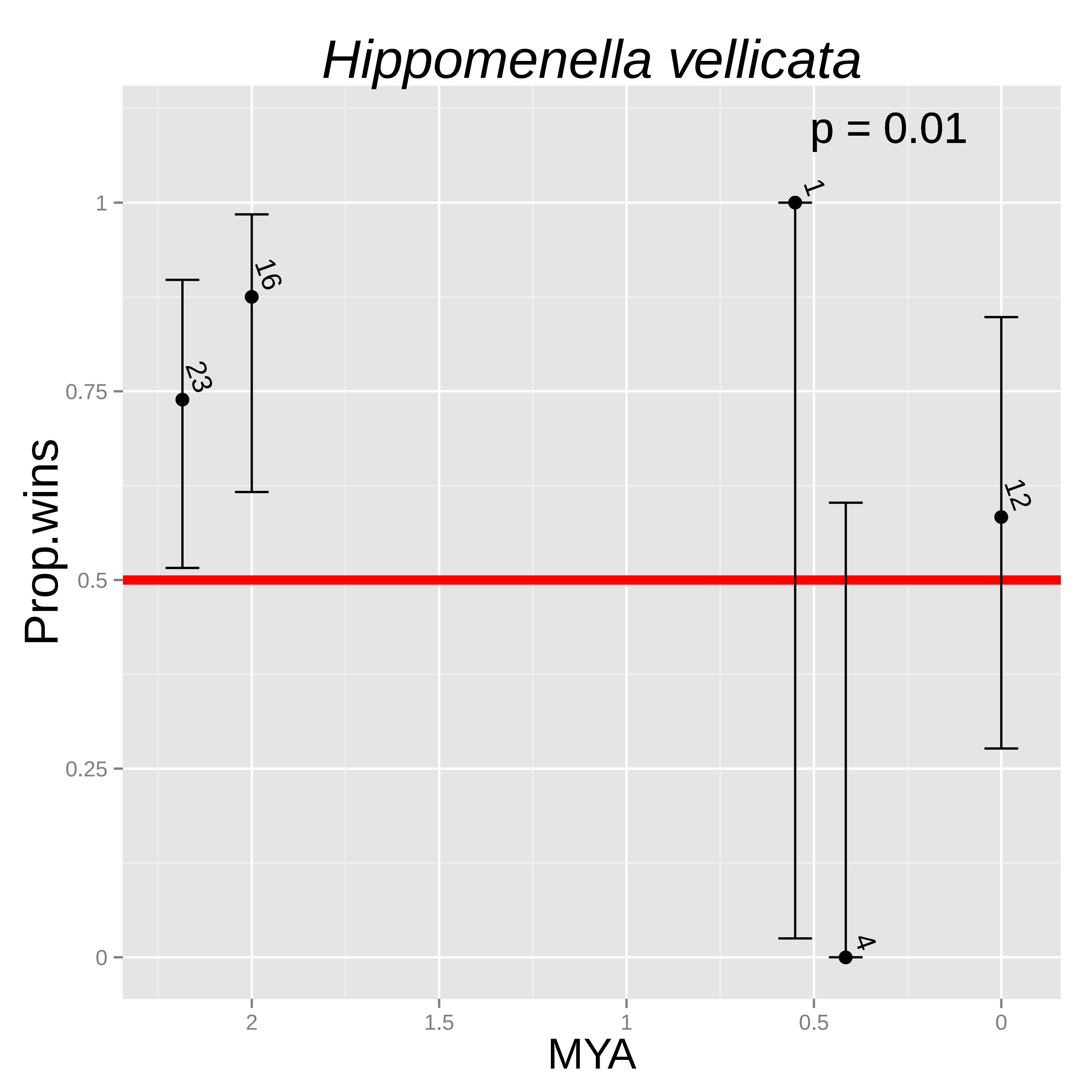

Hippomenella vellicata
p = 0.01
Prop.wins
MYA
1
0.75
0.5
0.25
0
2
1.5
1
0.5
0
23
16
1
4
12

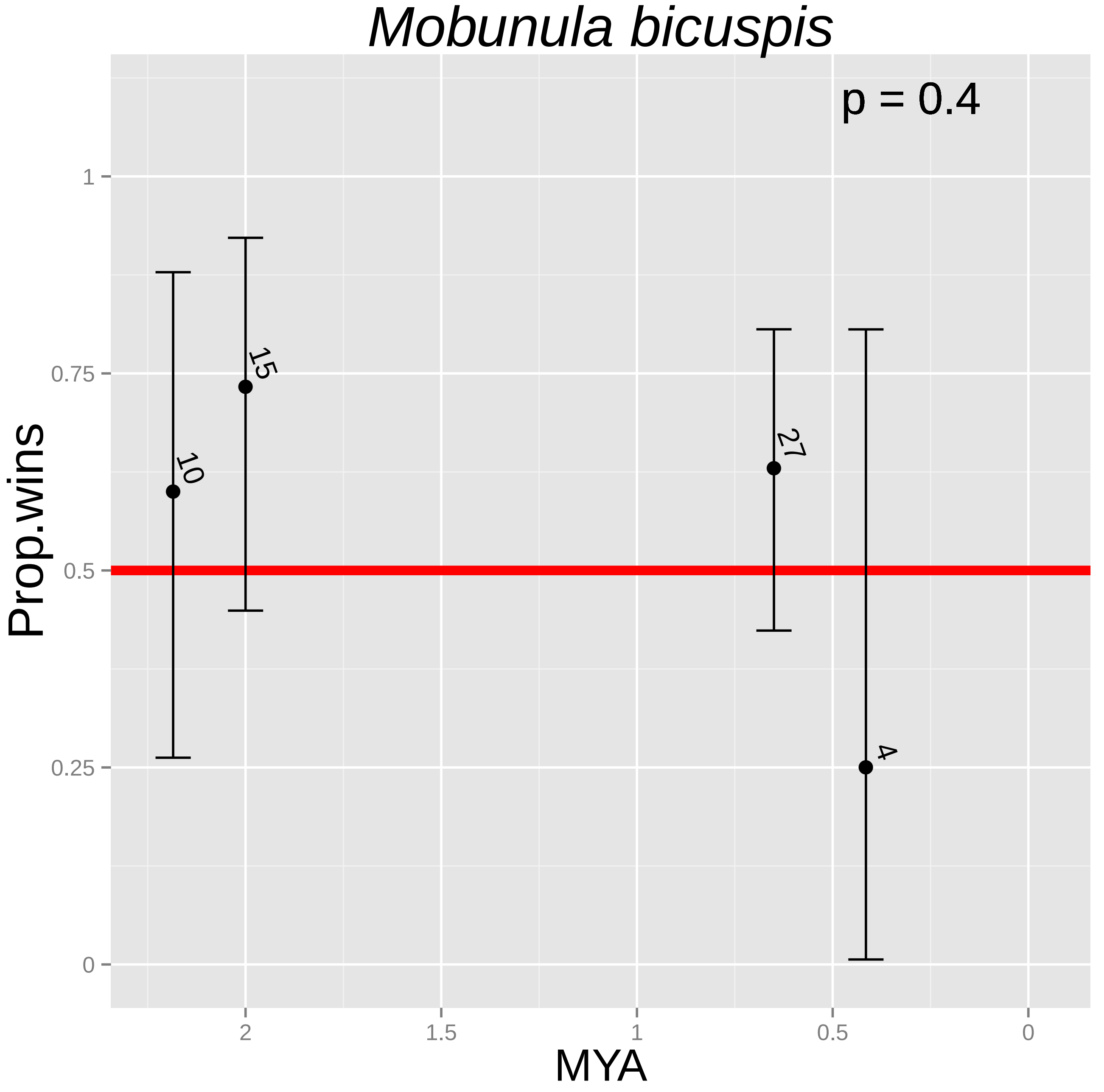

Mobunula bicuspis
p = 0.4
Prop.wins
1
0.75
0.5
0.25
0
10
15
27
4
2
1.5
1
0.5
0
MYA

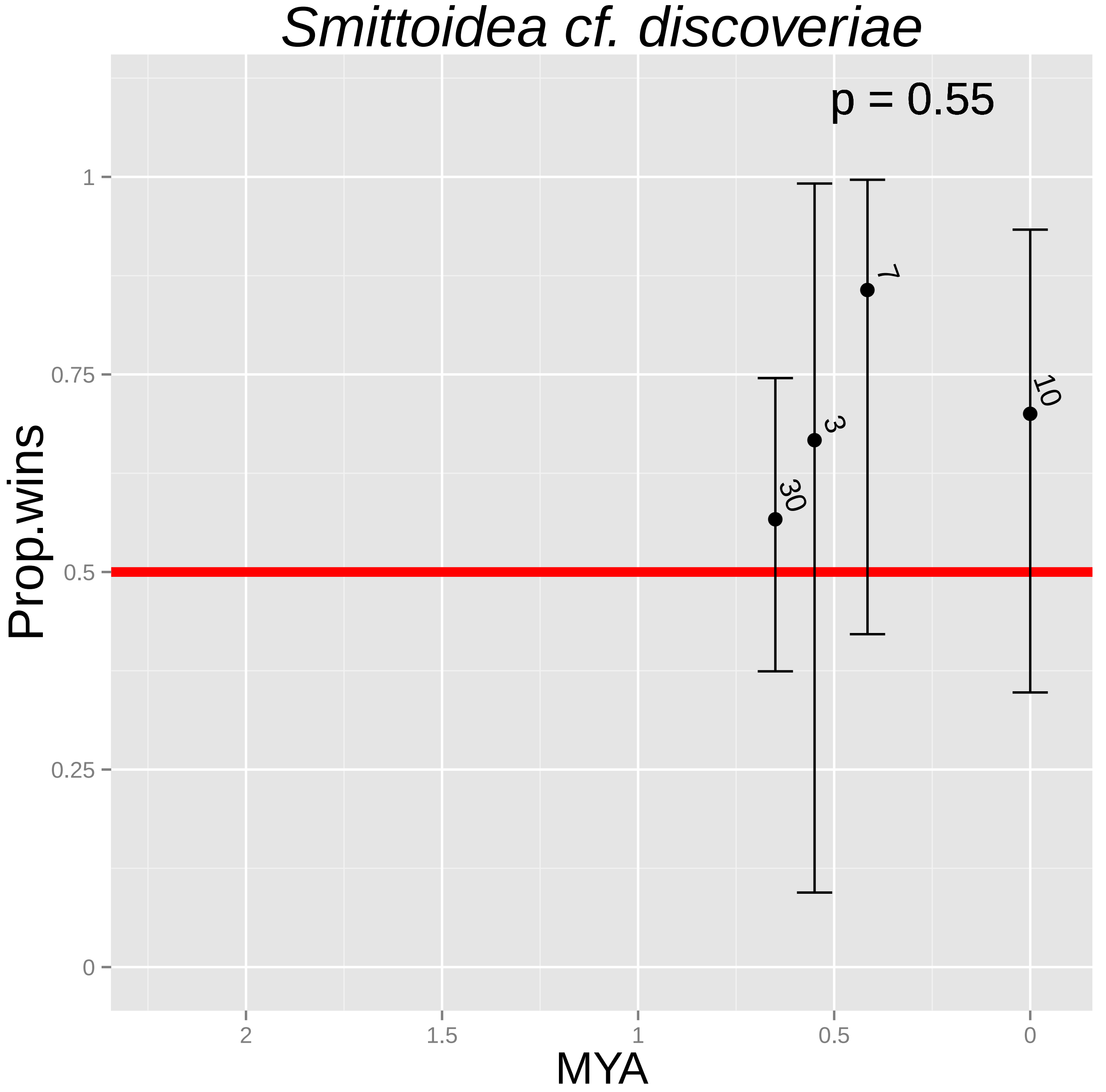
Smittoidea cf. discoveriae
p = 0.55
Prop.wins
MYA
1
0.75
0.5
0.25
0
2
1.5
1
0.5
0
30
3
7
10

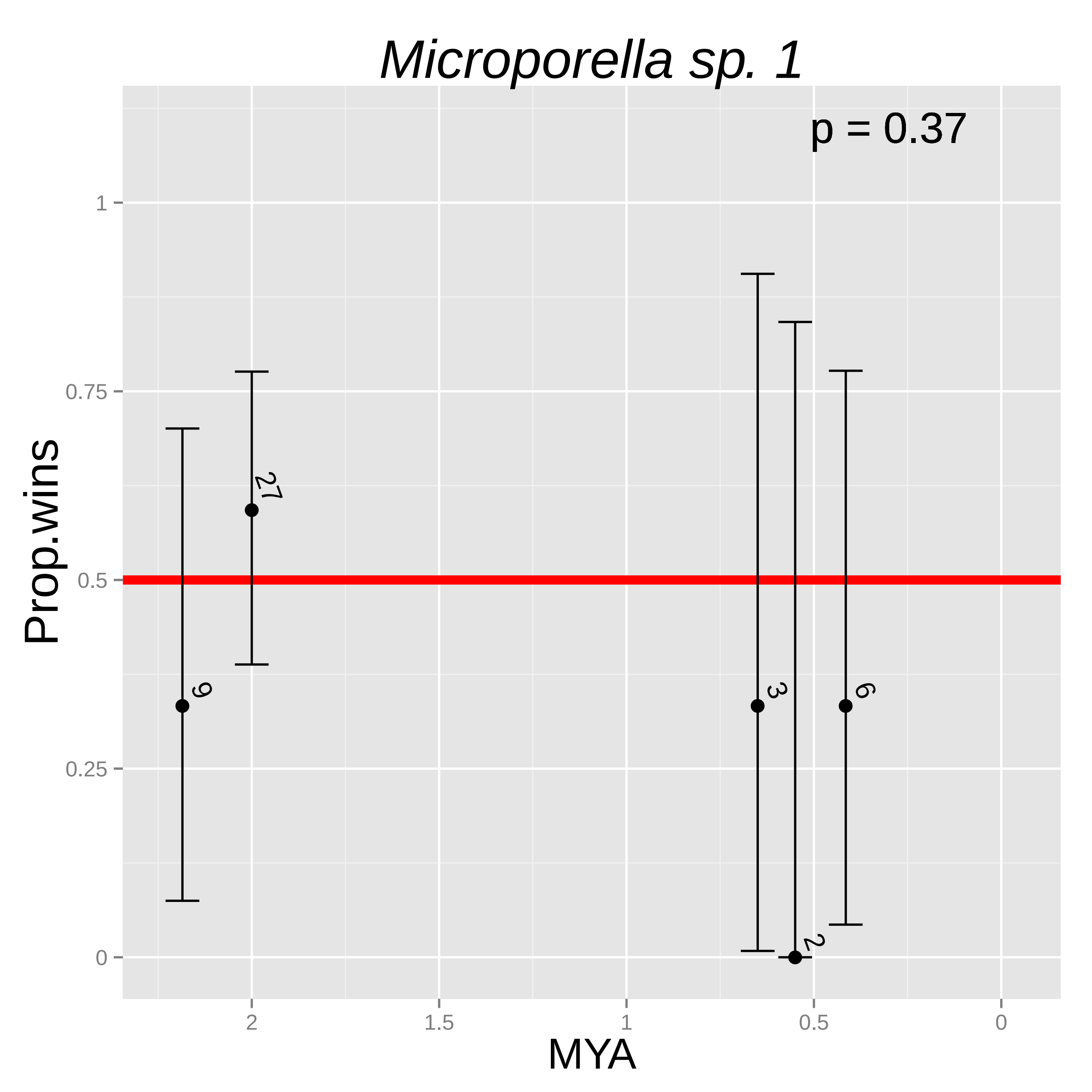

Microporella sp. 1
p = 0.37
Prop.wins
MYA
1
0.75
0.5
0.25
0
2
1.5
1
0.5
0
27
9
3
2
6

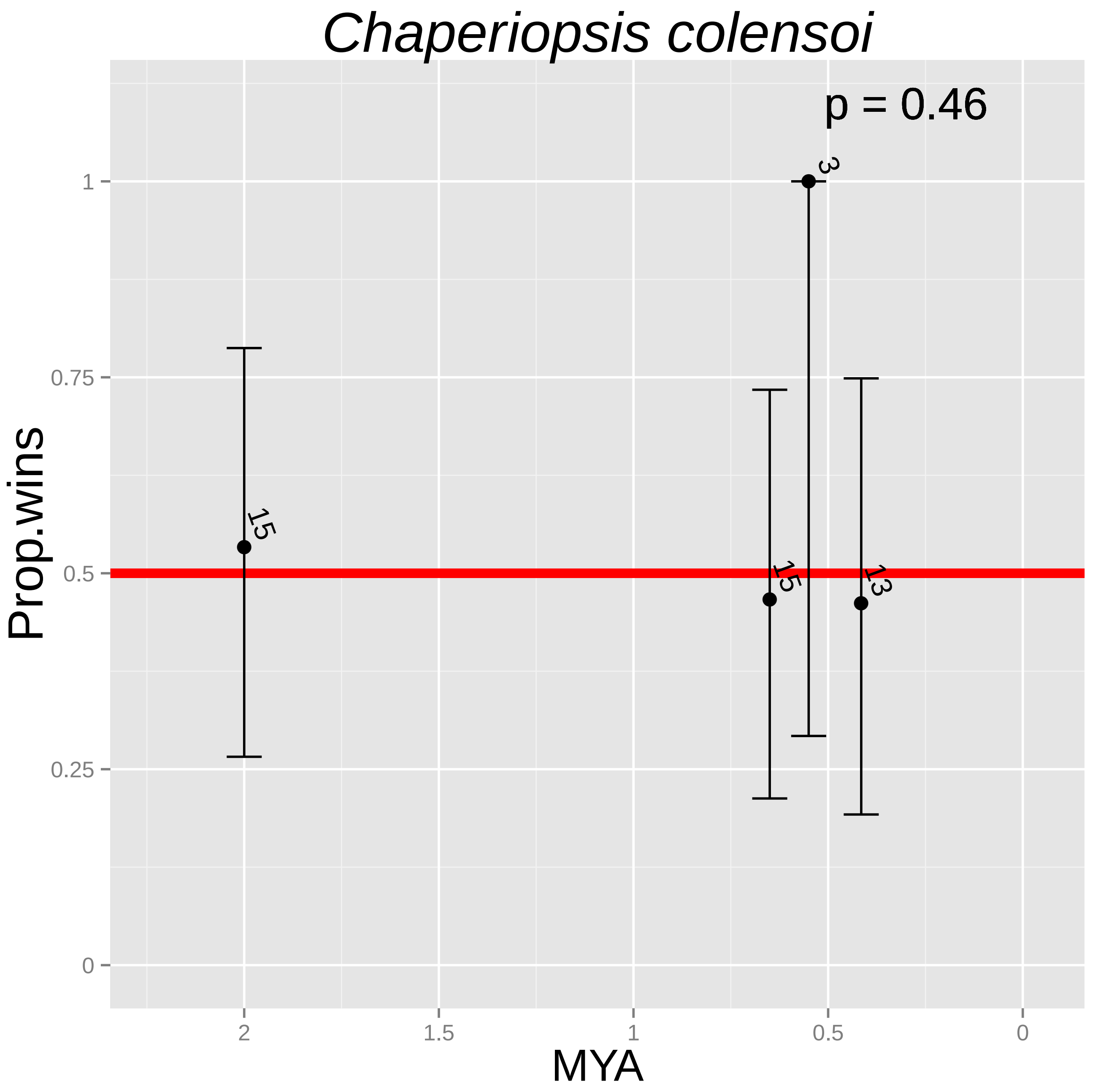

Chaperiopsis colensoi
p = 0.46
Prop.wins
MYA
1
0.75
0.5
0.25
0
2
1.5
1
0.5
0
15
15
3
13

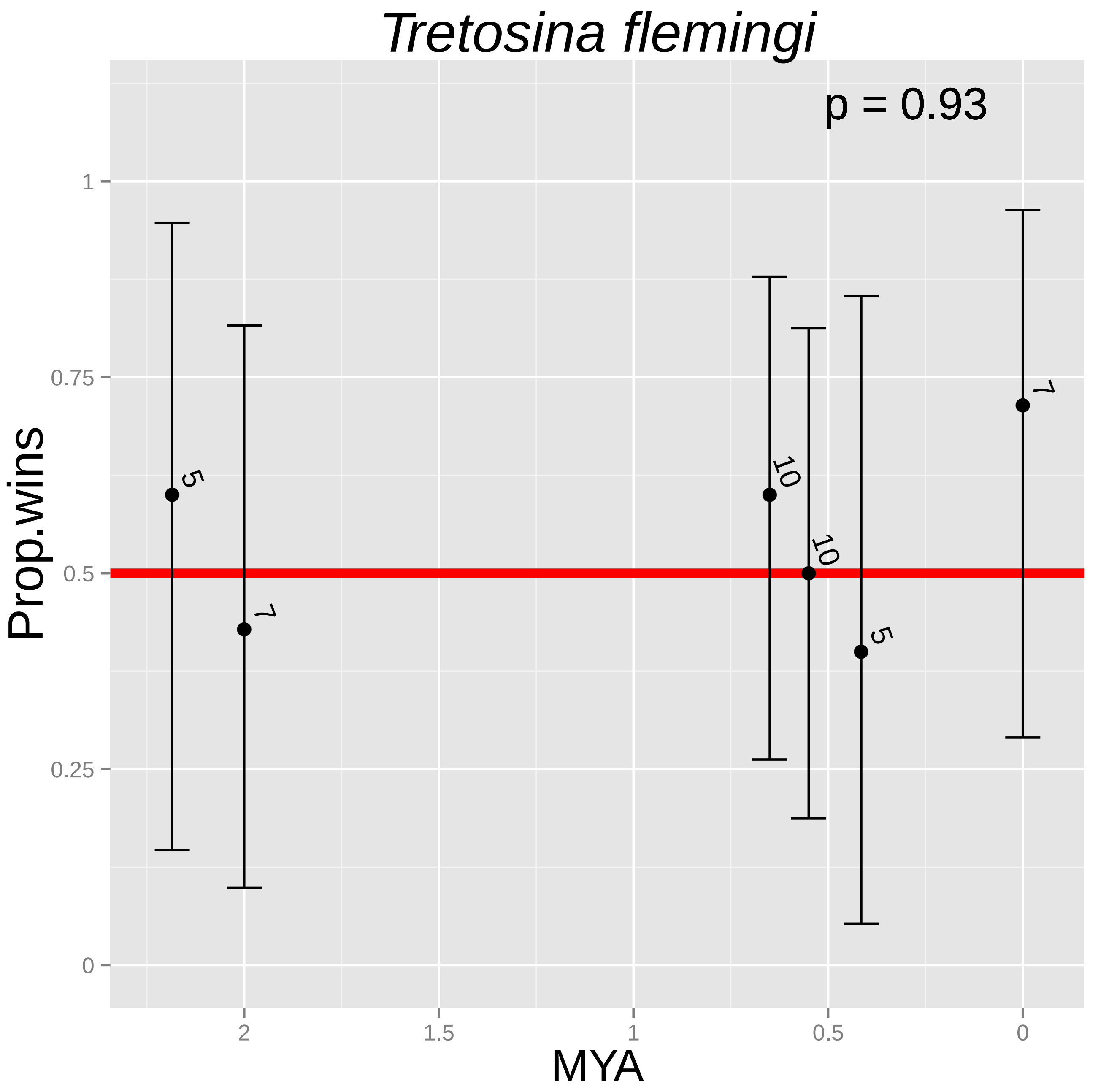
Tretosina flemingi
p = 0.93
Prop.wins
MYA
1
0.75
0.5
0.25
0
2
1.5
1
0.5
0
5
7
10
10
5
7

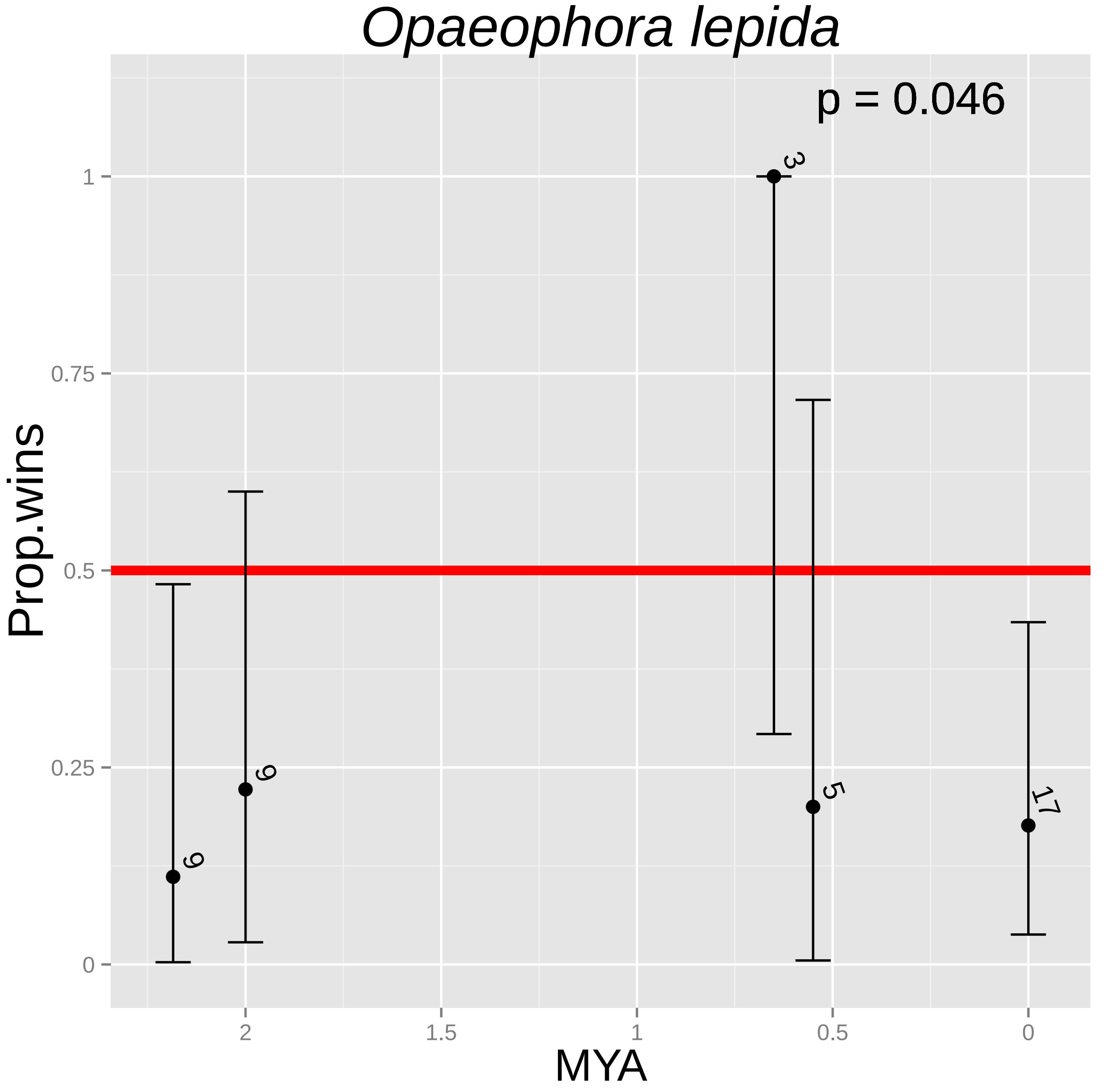

Opaeophora lepida
p = 0.046
Prop.wins
MYA
1
0.75
0.5
0.25
0
2
1.5
1
0.5
0
3
9
9
5
17

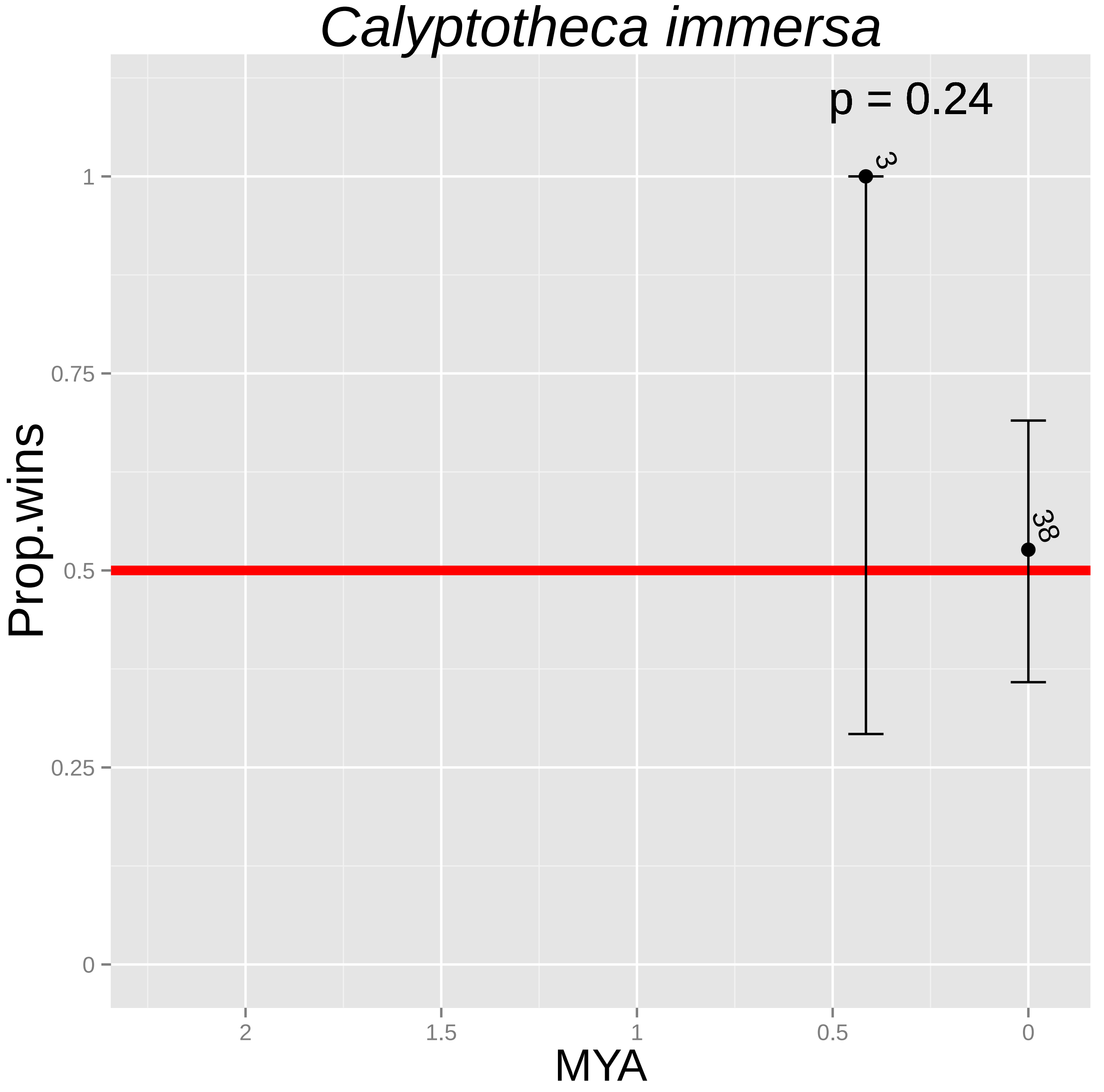

Calyptotheca immersa
p = 0.24
3
38
Prop.wins
1
0.75
0.5
0.25
0
2
1.5
1
0.5
0
MYA

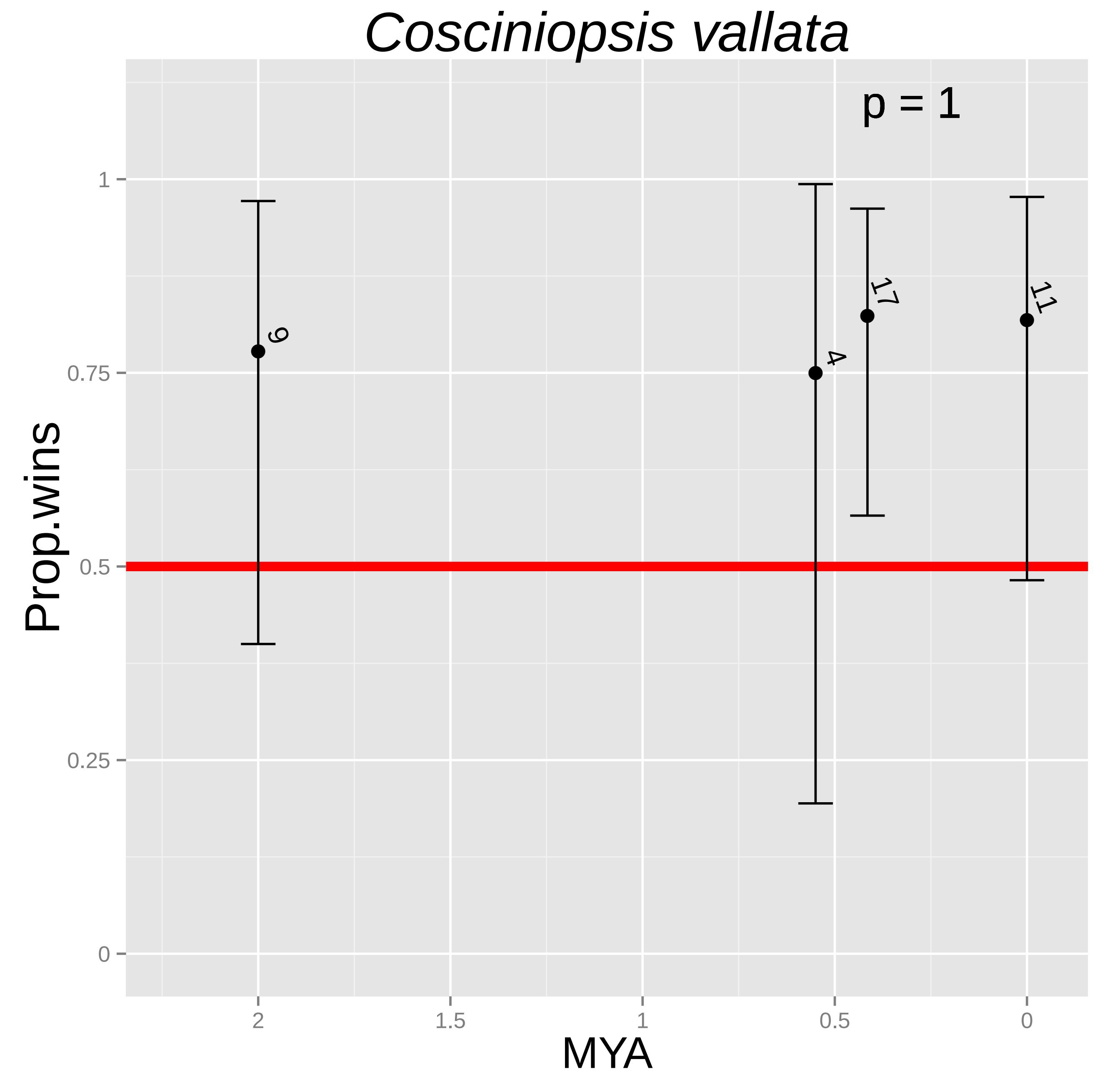

Cosciniopsis vallata
p = 1
Prop.wins
MYA
0
0.25
0.5
0.75
1
2
1.5
1
0.5
0
9
4
17
11

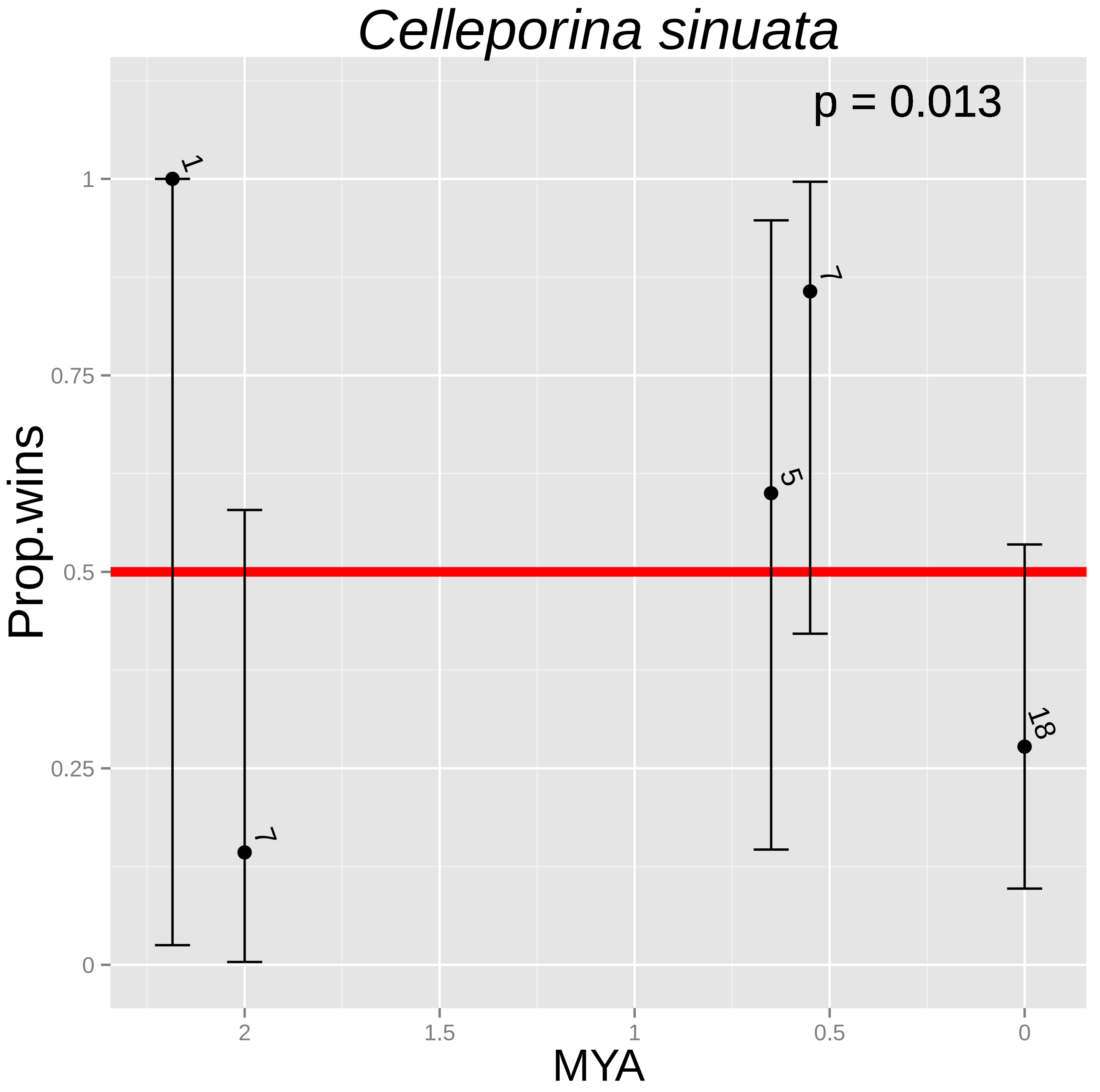
Celleporina sinuata
p = 0.013
Prop.wins
MYA
1
0.75
0.5
0.25
0
2
1.5
1
0.5
0
1
7
5
7
18

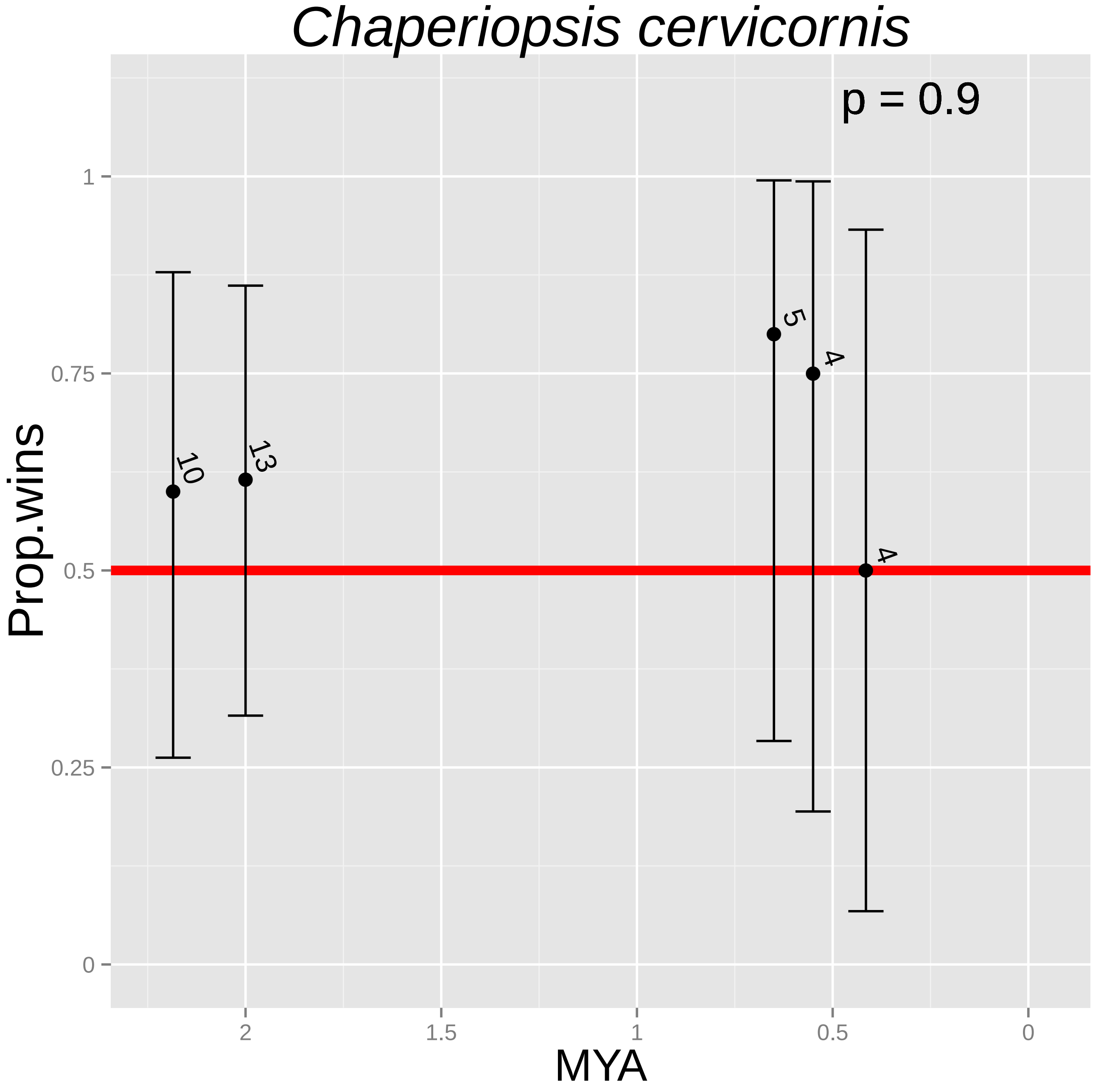

Chaperiopsis cervicornis
p = 0.9
Prop.wins
MYA
1
0.75
0.5
0.25
0
2
1.5
1
0.5
0
10
13
5
4
4

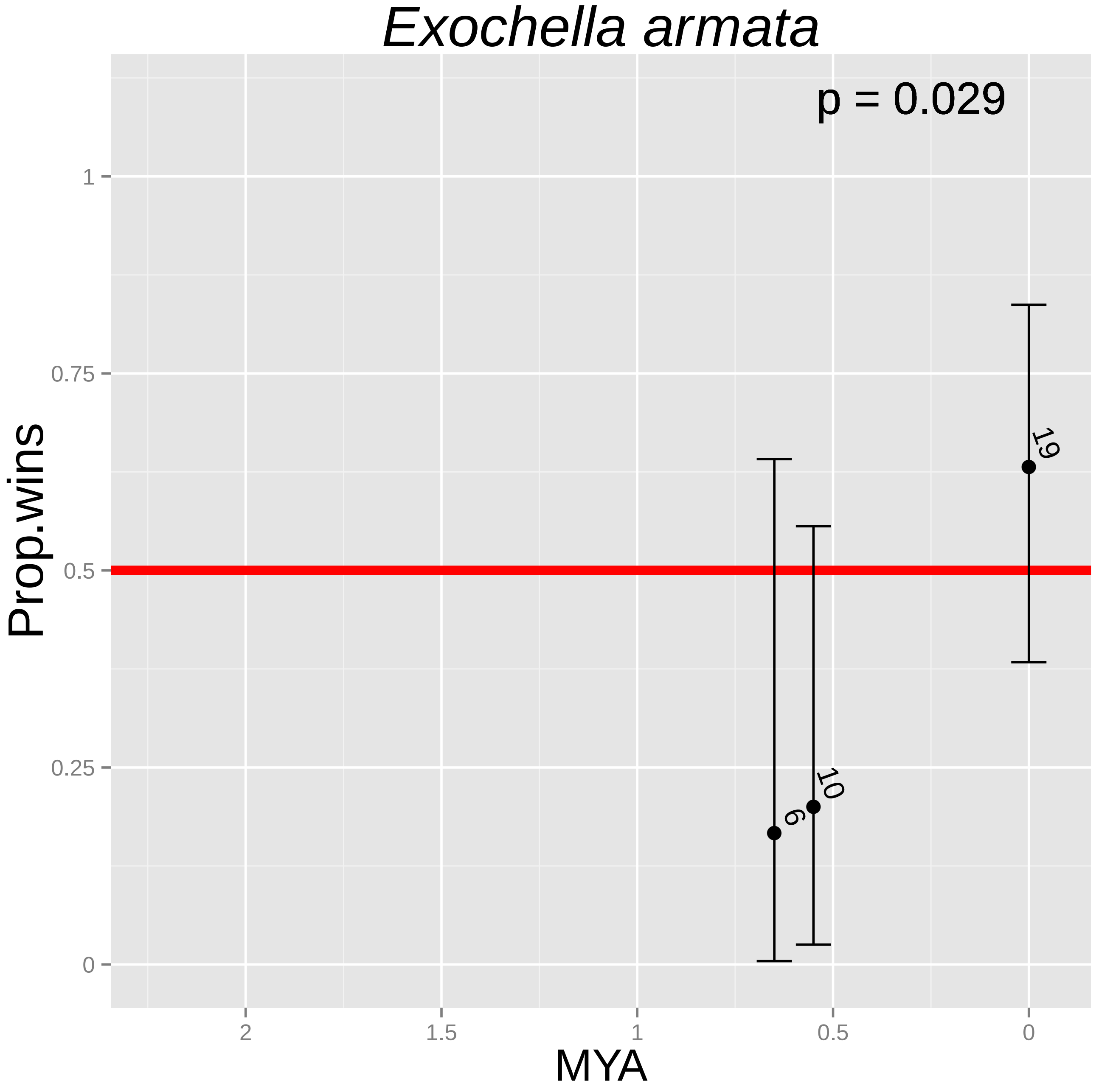

Exochella armata
p = 0.029
Prop.wins
1
0.75
0.5
0.25
0
19
10
6
2
1.5
1
0.5
0
MYA

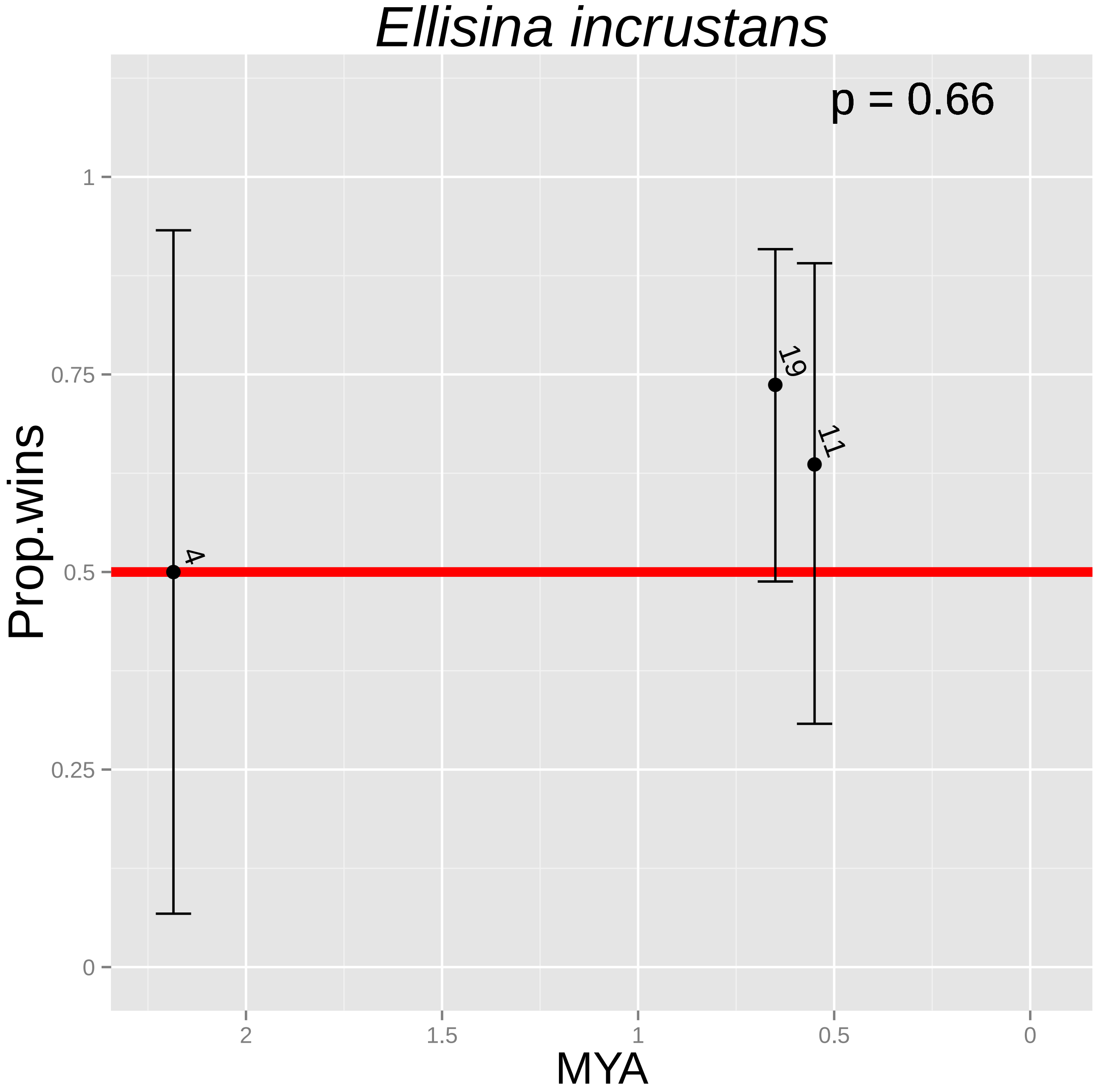

Ellisina incrustans
p = 0.66
Prop.wins
1
0.75
0.5
0.25
0
4
19
11
2
1.5
1
0.5
0
MYA

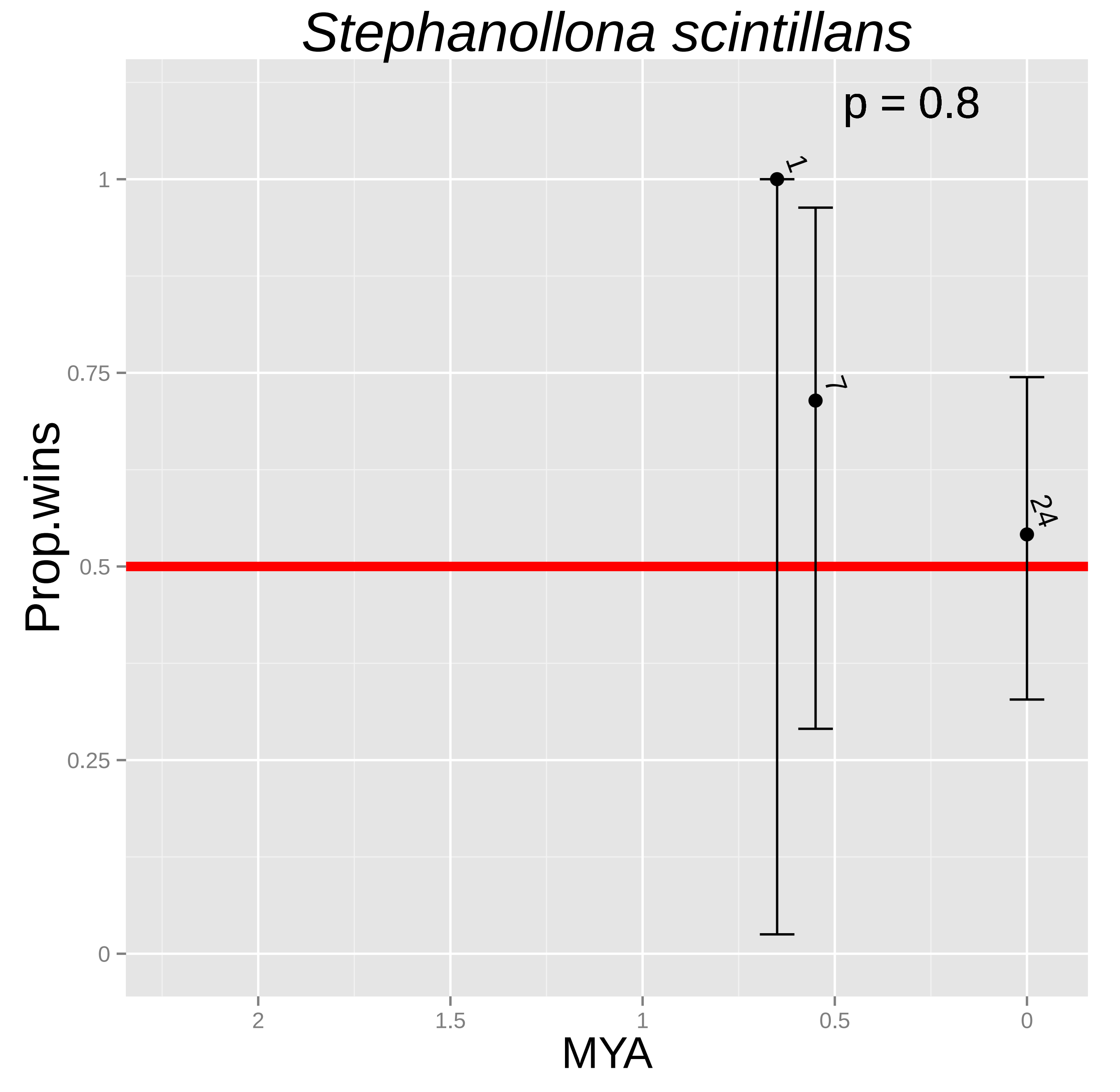

Stephanollona scintillans
p = 0.8
Prop.wins
MYA
1
0.75
0.5
0.25
0
2
1.5
1
0.5
0
1
7
24

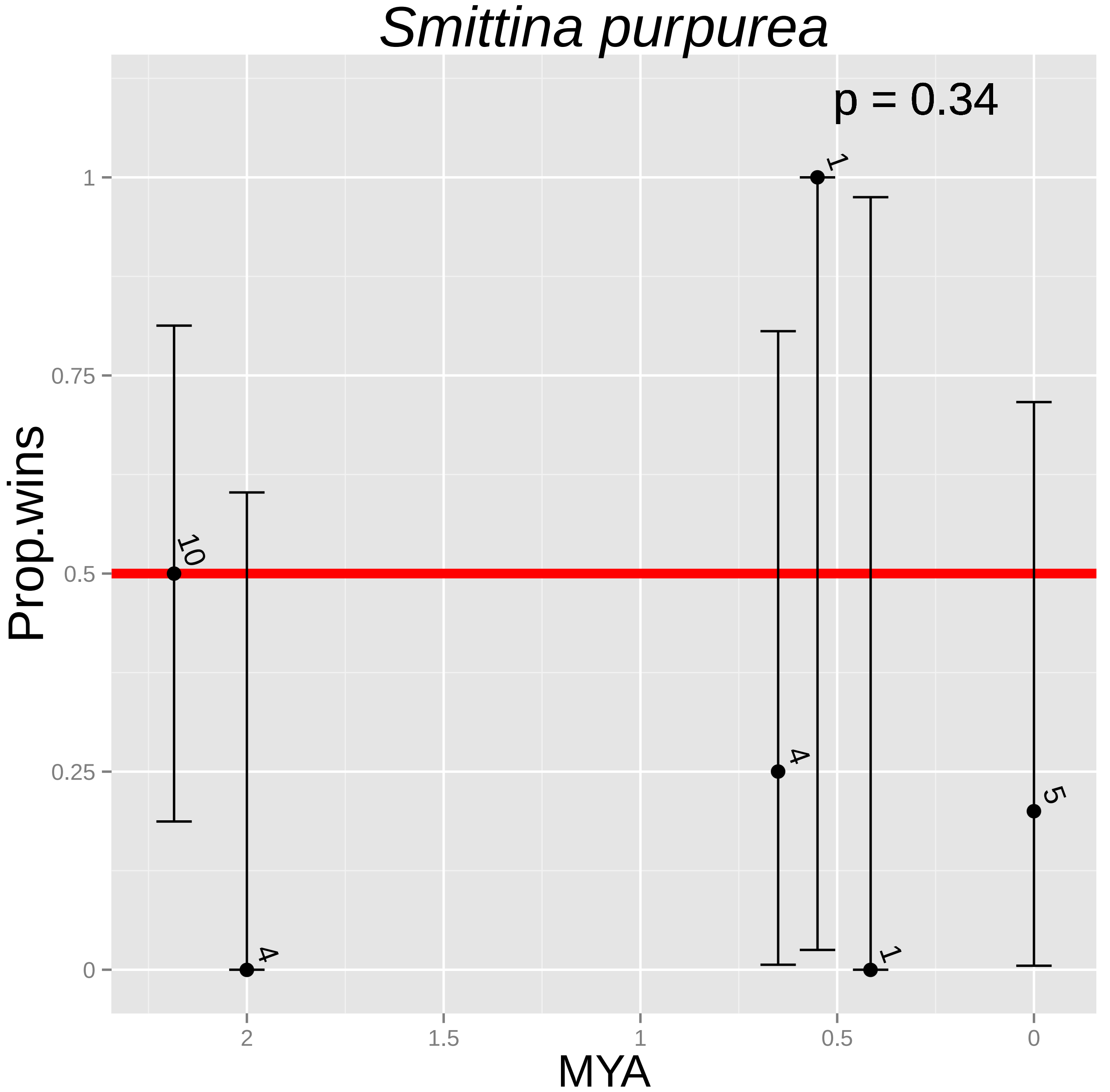

Smittina purpurea
p = 0.34
Prop.wins
MYA
0
0.25
0.5
0.75
1
2
1.5
1
0.5
0
10
4
4
1
1
5

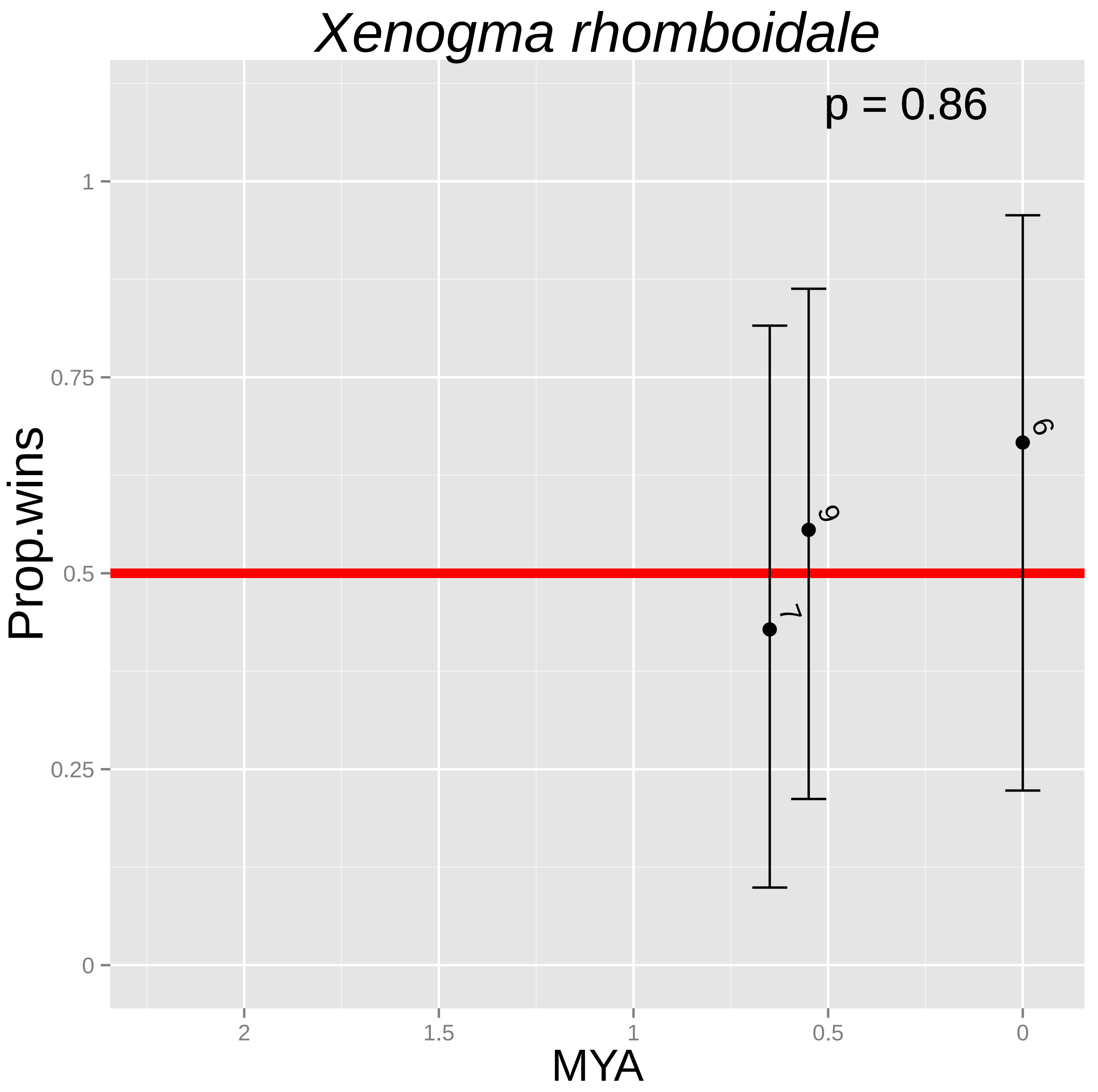

Xenogma rhomboidale
p = 0.86
Prop.wins
MYA
1
0.75
0.5
0.25
0
2
1.5
1
0.5
0

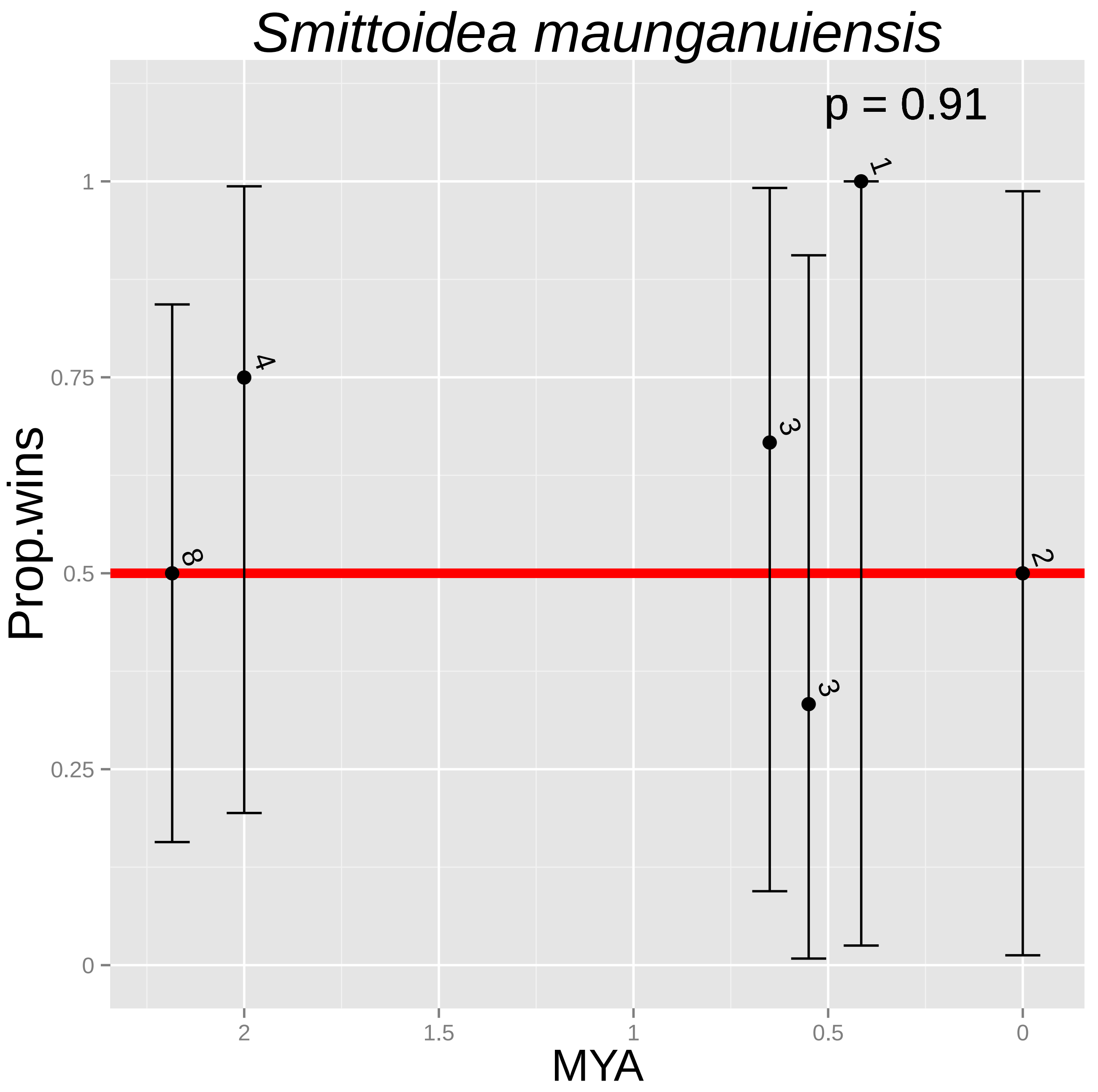

Smittoidea maunganuiensis
p = 0.91
Prop.wins
MYA
1
0.75
0.5
0.25
0
2
1.5
1
0.5
0
8
4
3
3
1
2

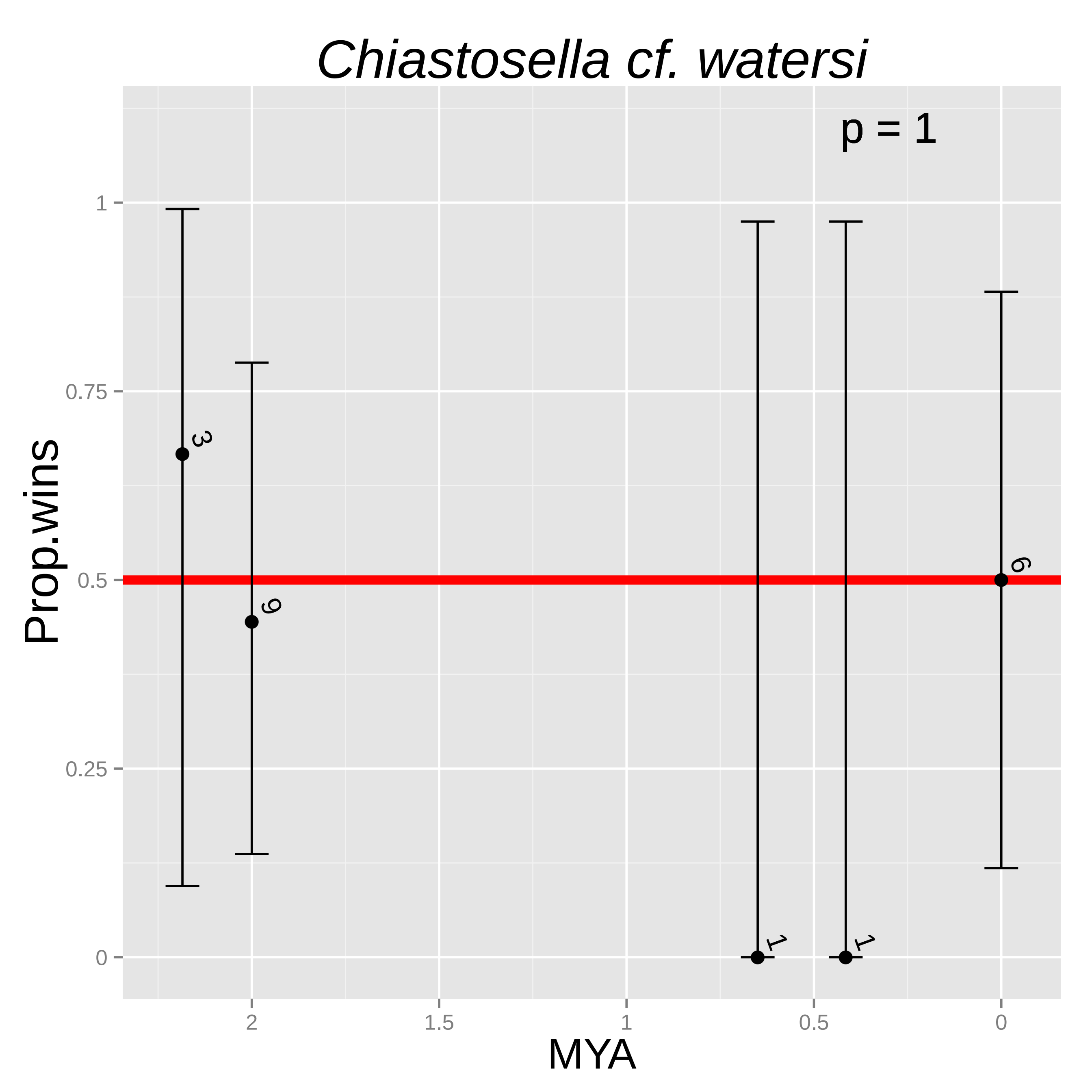

Chiastosella cf. watersi
p = 1
Prop.wins
MYA
1
0.75
0.5
0.25
0
2
1.5
1
0.5
0
3
9
1
1
6

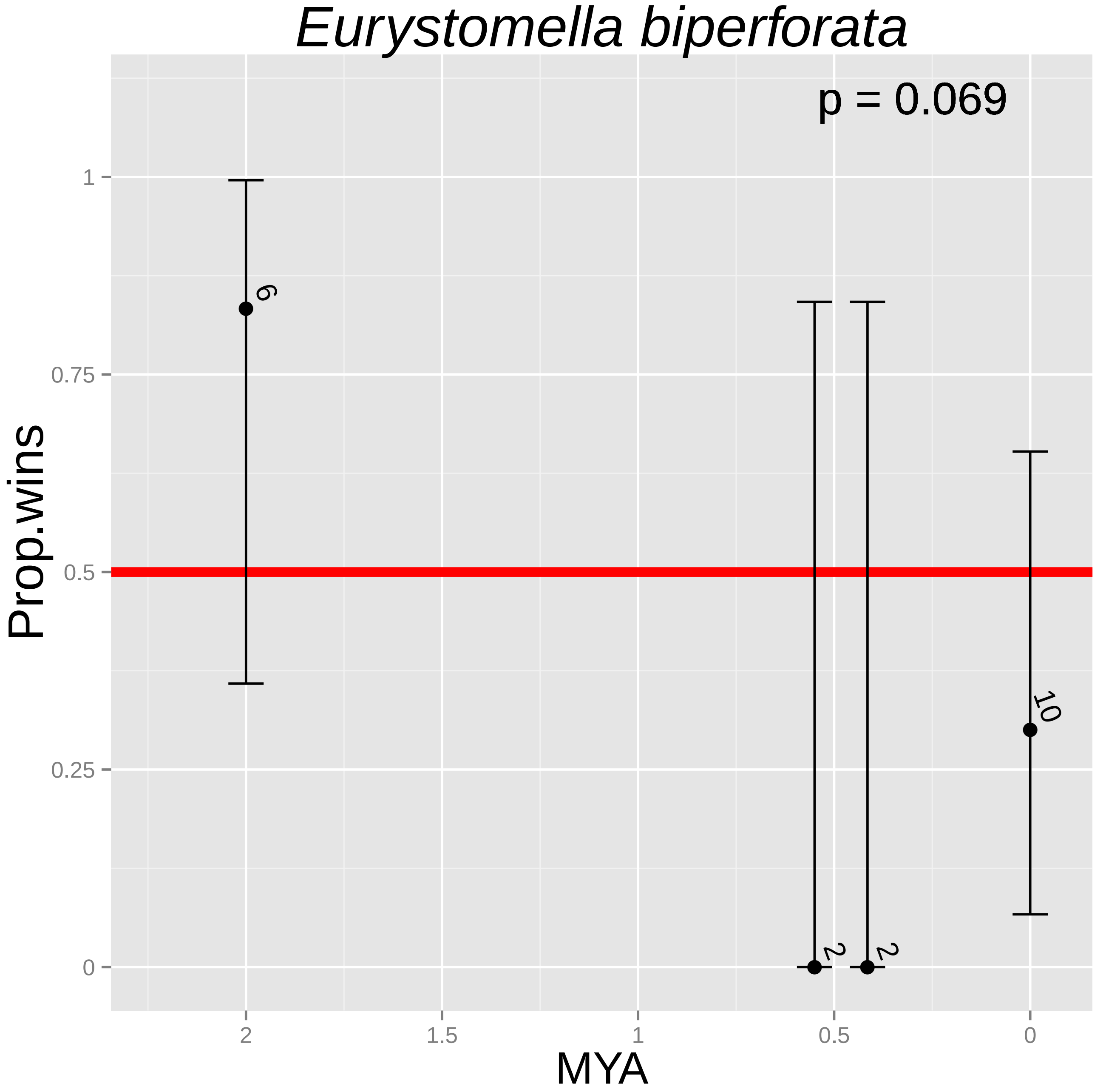

Eurystomella biperforata
p = 0.069
Prop.wins
MYA
1
0.75
0.5
0.25
0
2
1.5
1
0.5
0
6
2
2
10

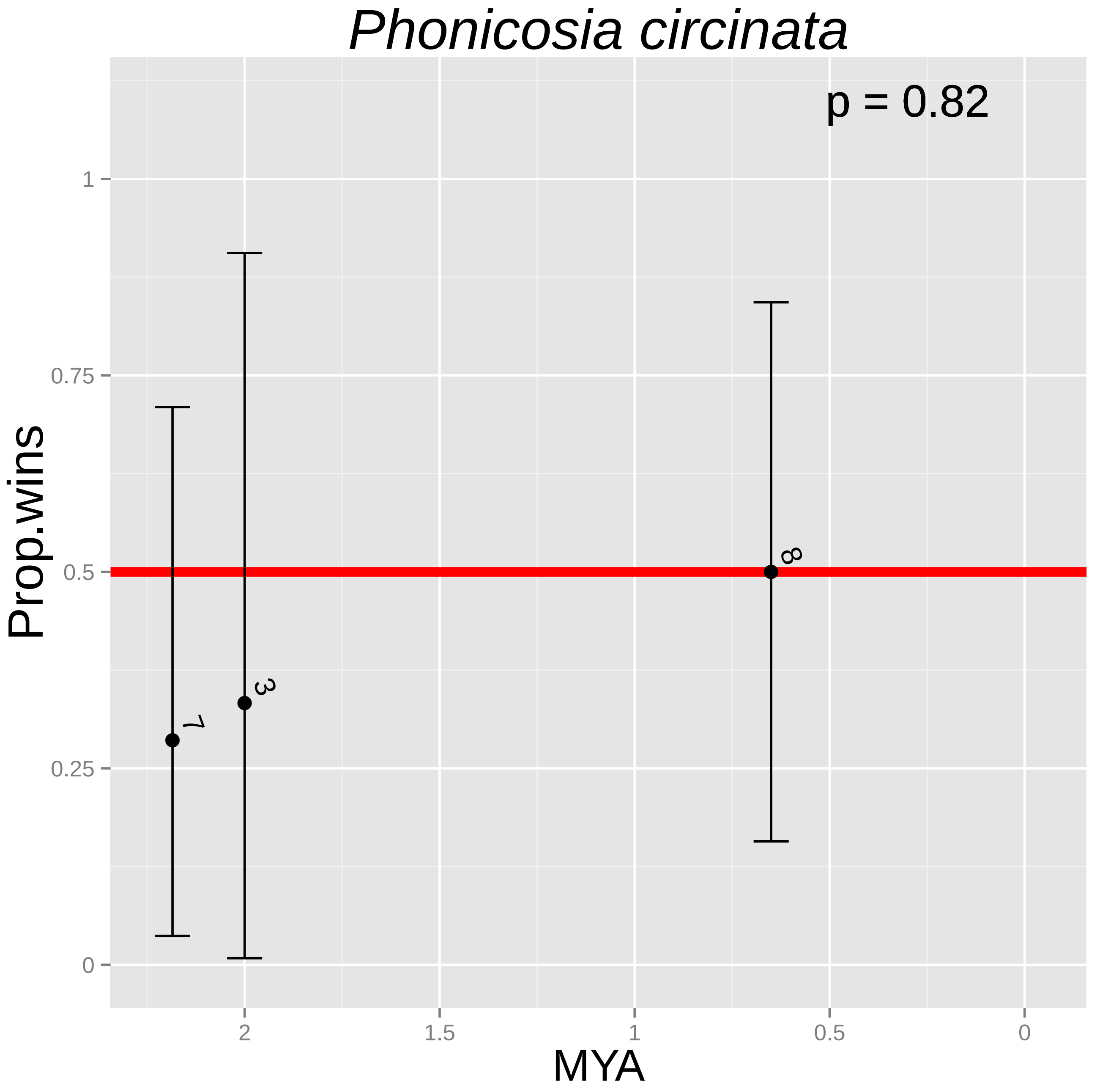

Phonicosia circinata
p = 0.82
Prop.wins
1
0.75
0.5
0.25
0
7
3
8
2
1.5
1
0.5
0
MYA

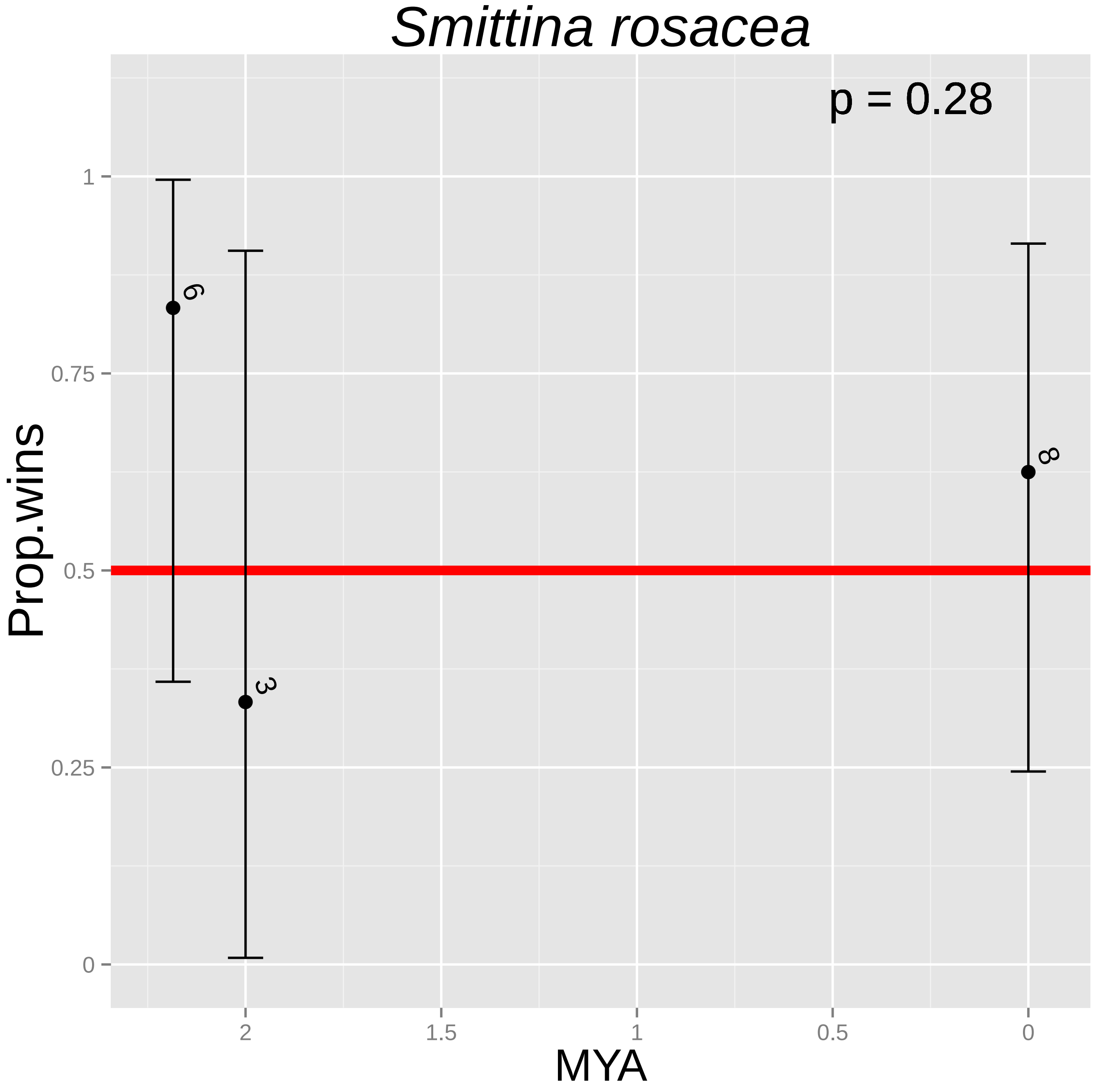

Smittina rosacea
p = 0.28
Prop.wins
MYA
1
0.75
0.5
0.25
0
2
1.5
1
0.5
0
6
3
8

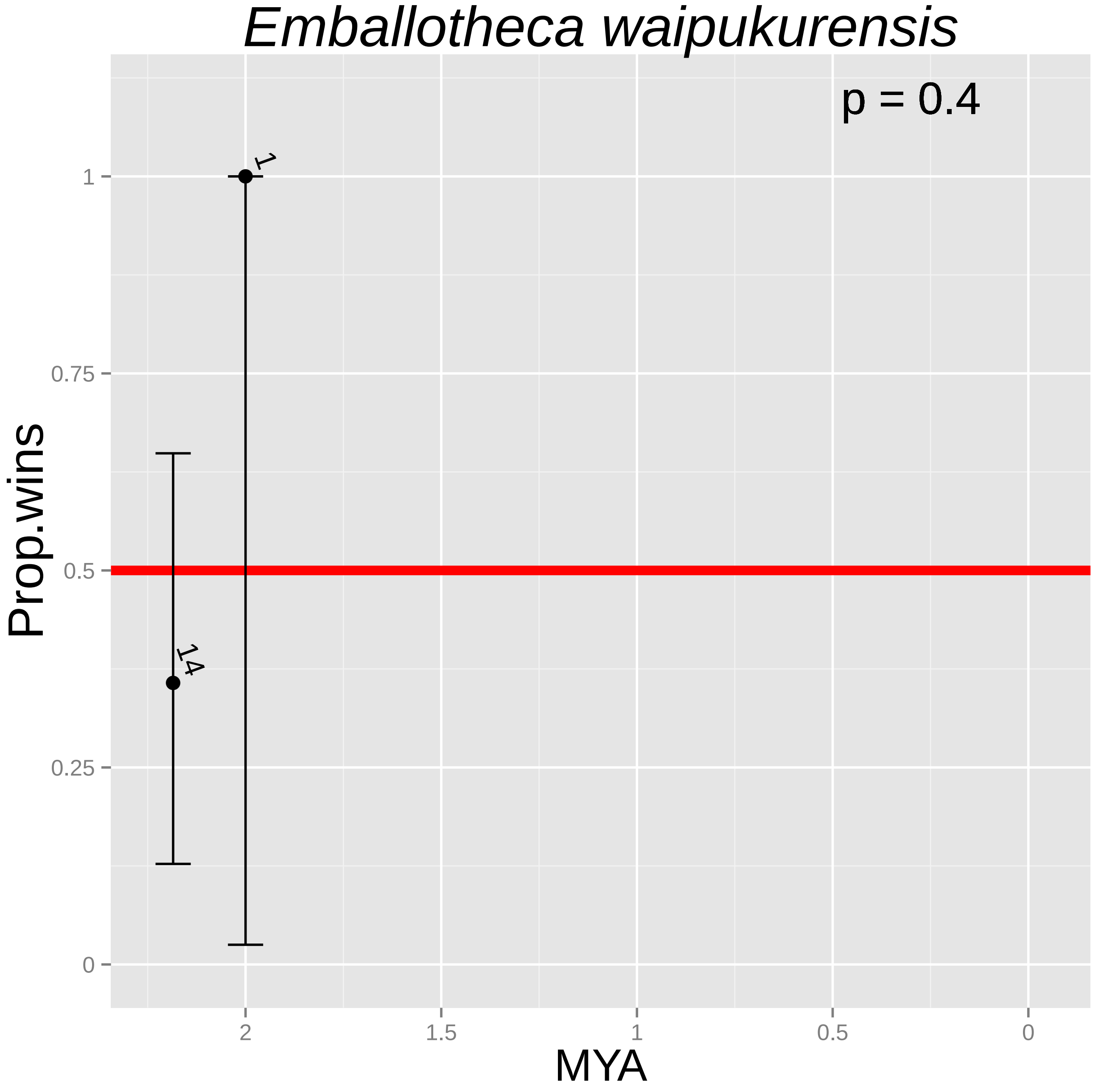

*Emballotheca waipukurensis*
p = 0.4
1
14
Prop.wins
1
0.75
0.5
0.25
0
2
1.5
1
0.5
0
MYA

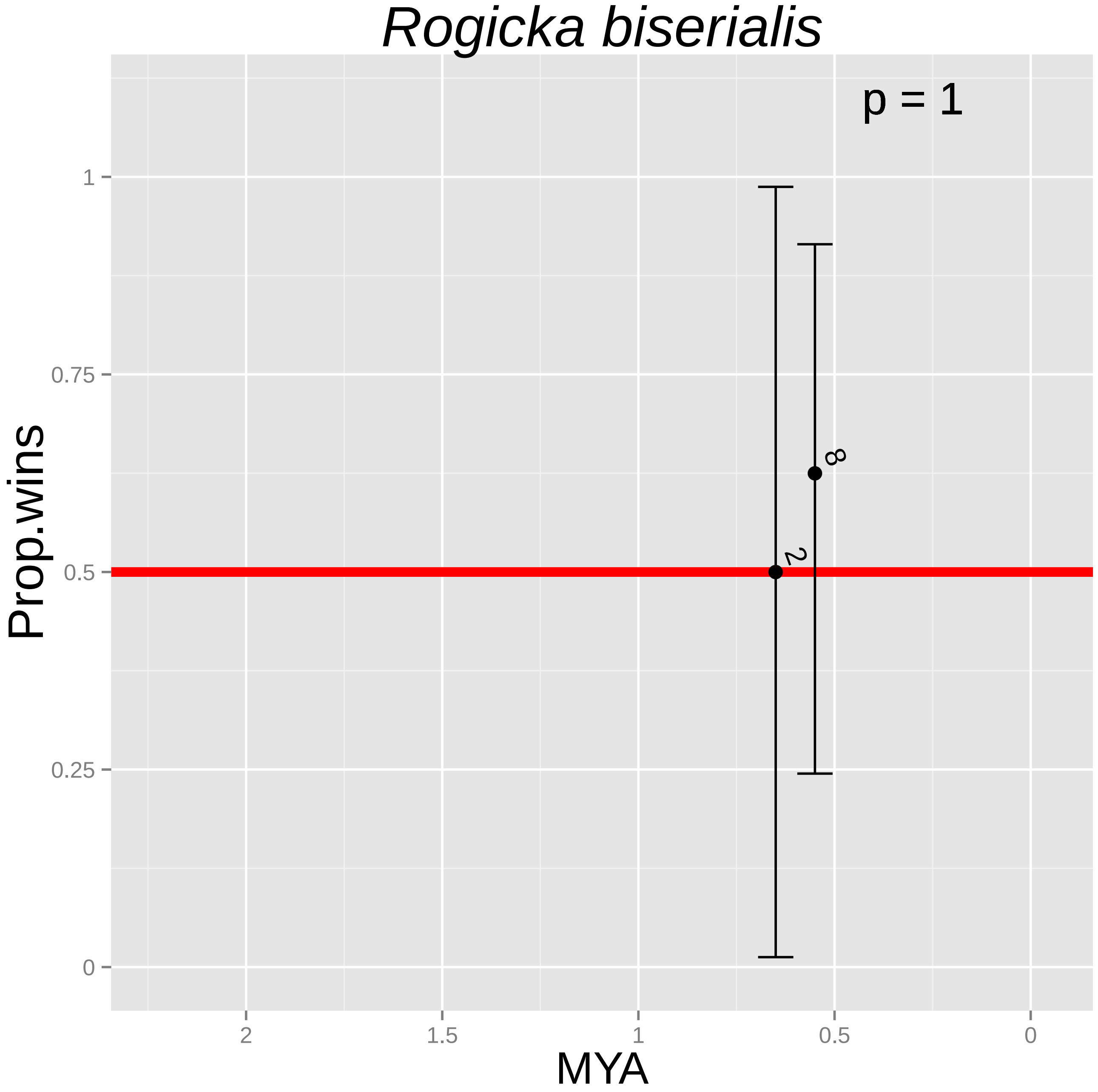
Rogicka biserialis
p = 1
Prop.wins
1
0.75
0.5
0.25
0
8
2
2
1.5
1
0.5
0
MYA

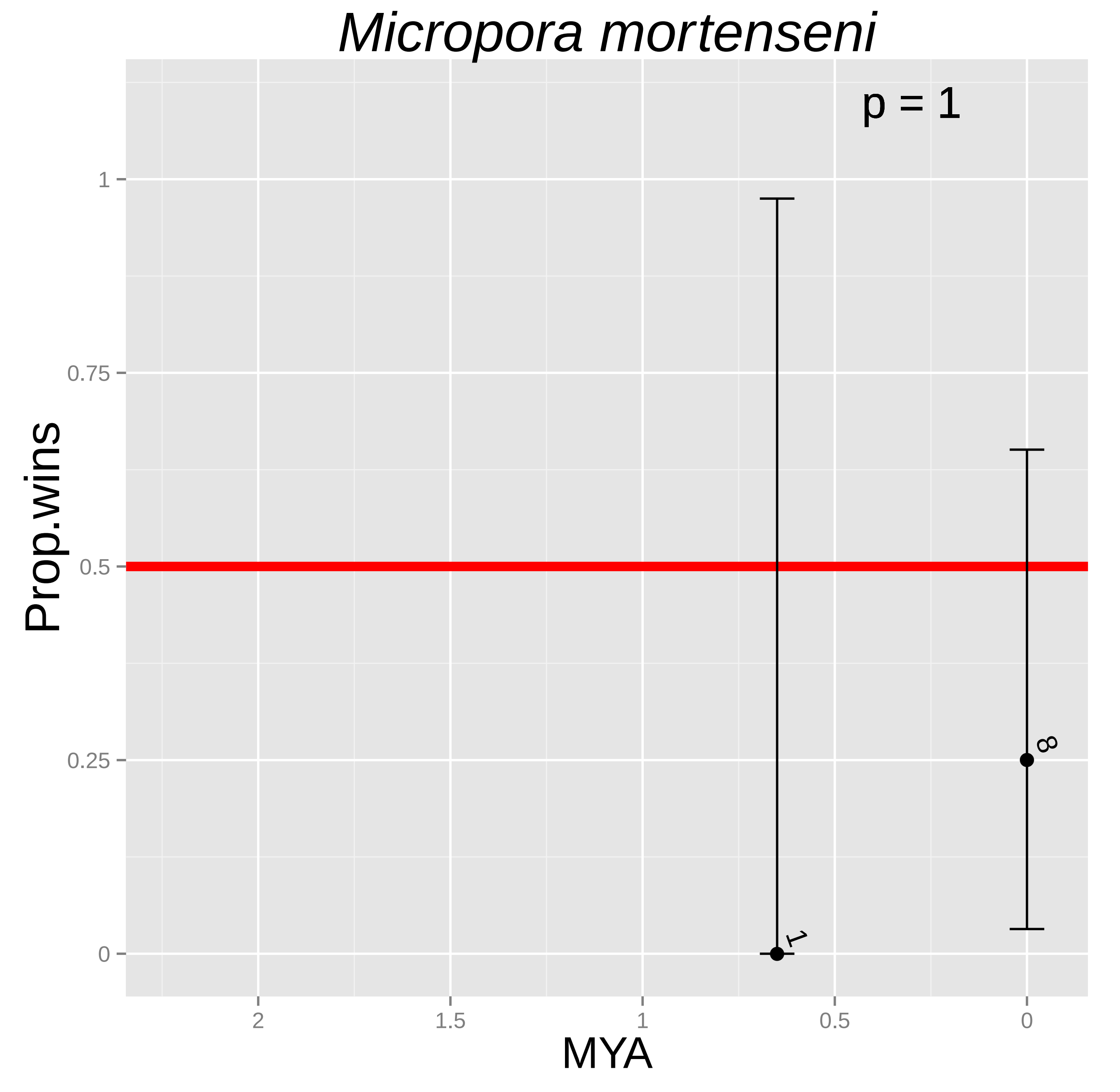
Micropora mortenseni
p = 1
Prop.wins
MYA
1
0.75
0.5
0.25
0
2
1.5
1
0.5
0
1
8

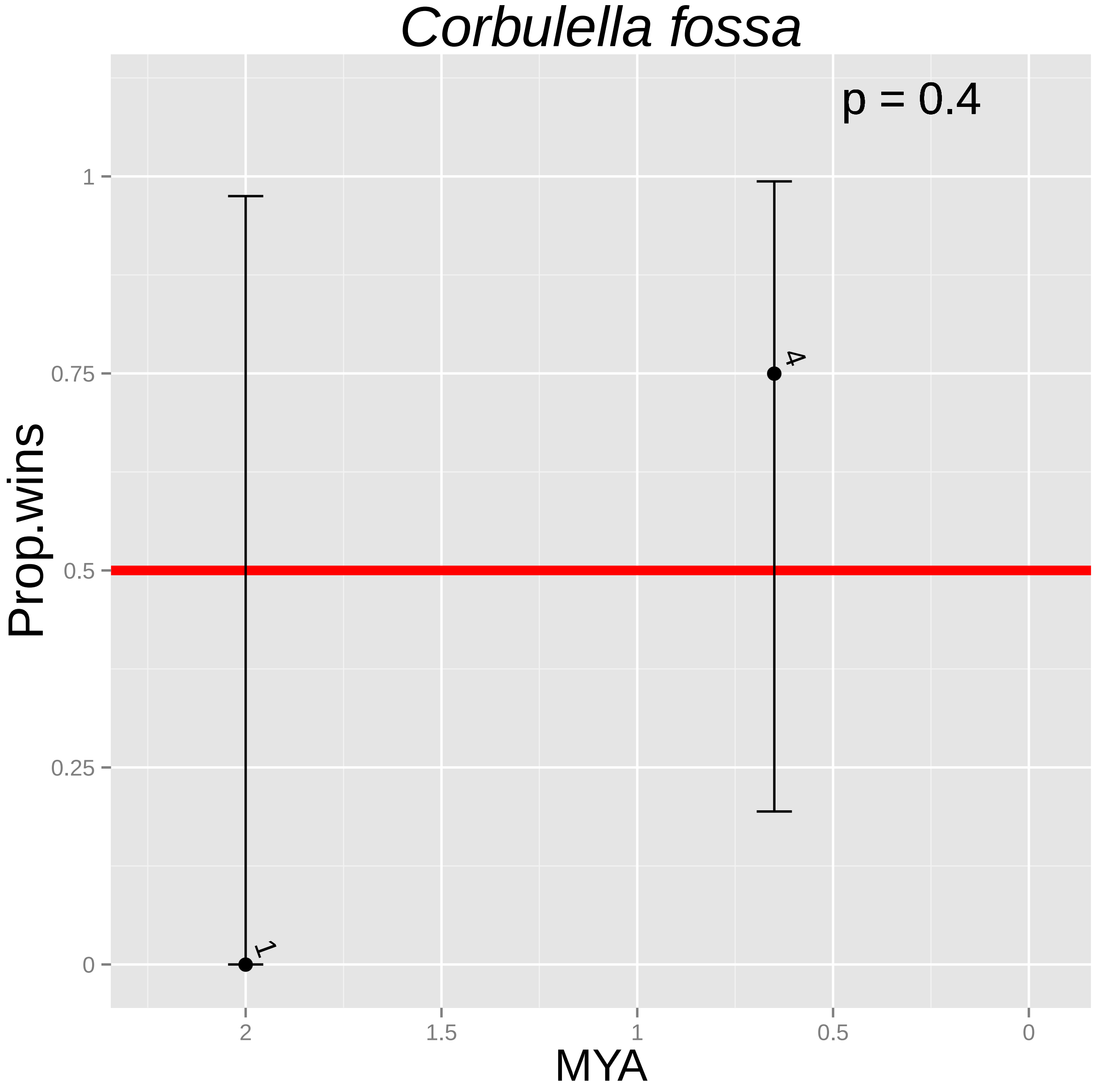

Corbulella fossa
p = 0.4
Prop.wins
MYA
1
0.75
0.5
0.25
0
2
1.5
1
0.5
0
4
1

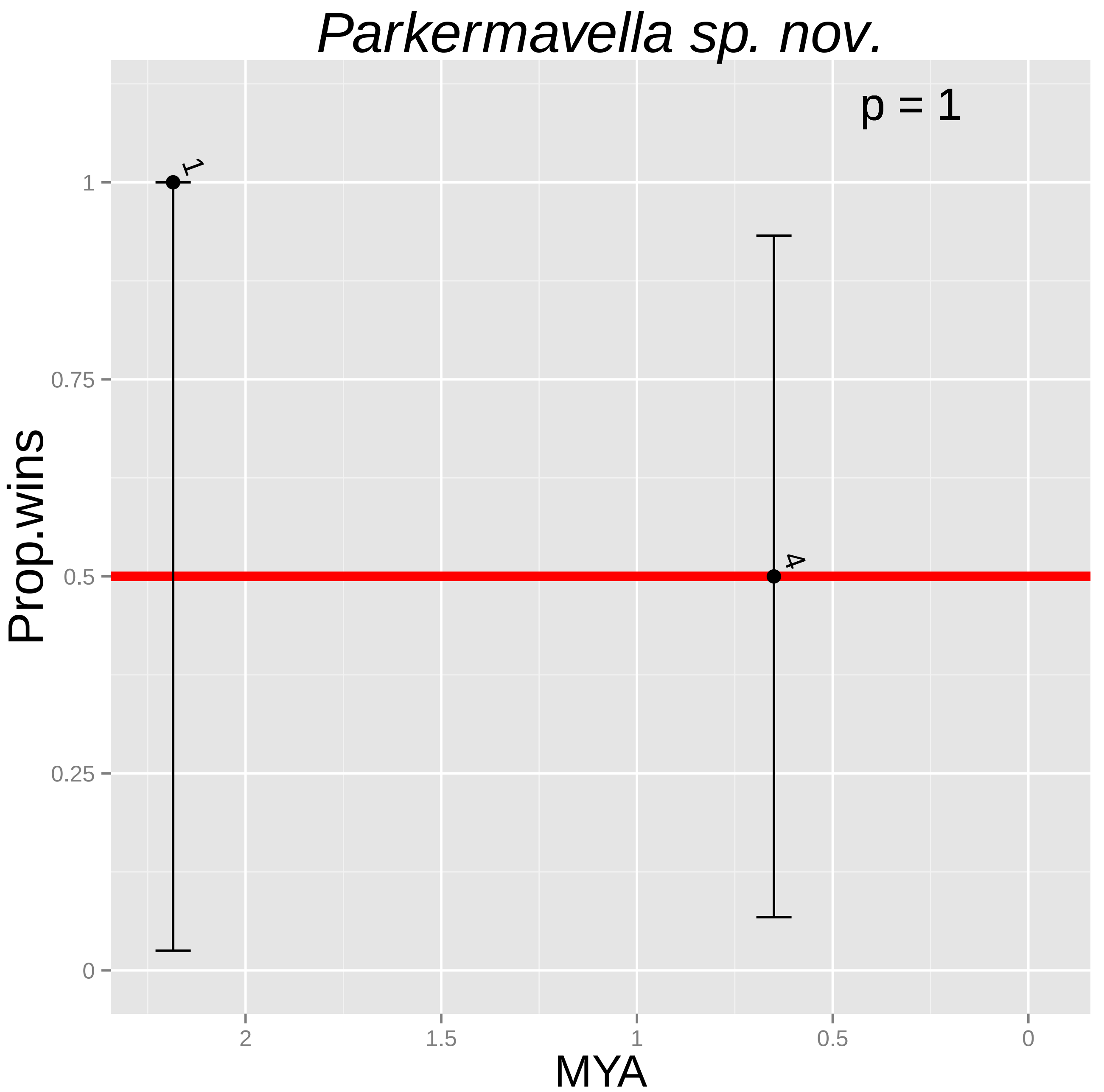

Parkermavella sp. nov.
p = 1
Prop.wins
1
0.75
0.5
0.25
0
MYA
2
1.5
1
0.5
0
1
4

**Fig. S2: Inter-generic win-proportions through time.**
Each panel plots the binomial probabilities and 95% confidence intervals of the interspecific win-proportions for genera represented by multiple species in our data. Red horizontal lines indicate the null hypothesis of 0.5 win-proportions. P-valuesstem from Fisher's Exact Test to test differences among the win proportions among binomial probabilities in each panel. Slanted numbers are the number of interspecific interactions contributing to plotted points and the associated confidence intervals. *Micropora, Celleporina* and *Osthimosia* were included here because we are certain they comprise of multiple species in our dataset, but some of which can only be distinguished using Scanning Electron Microscopy**.**

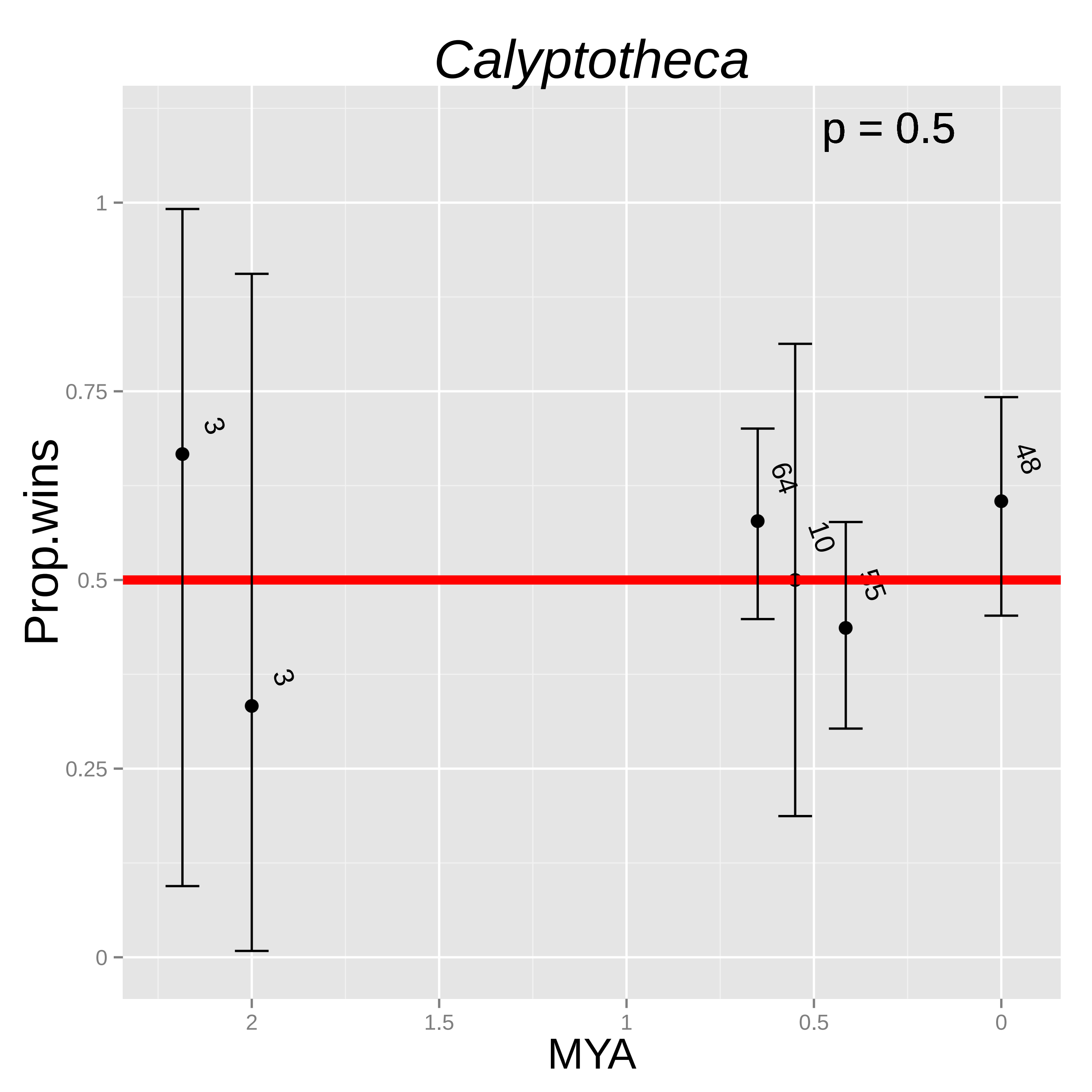

Calyptotheca
p = 0.5
Prop.wins
MYA
1
0.75
0.5
0.25
0
2
1.5
1
0.5
0
3
3
64
10
55
48

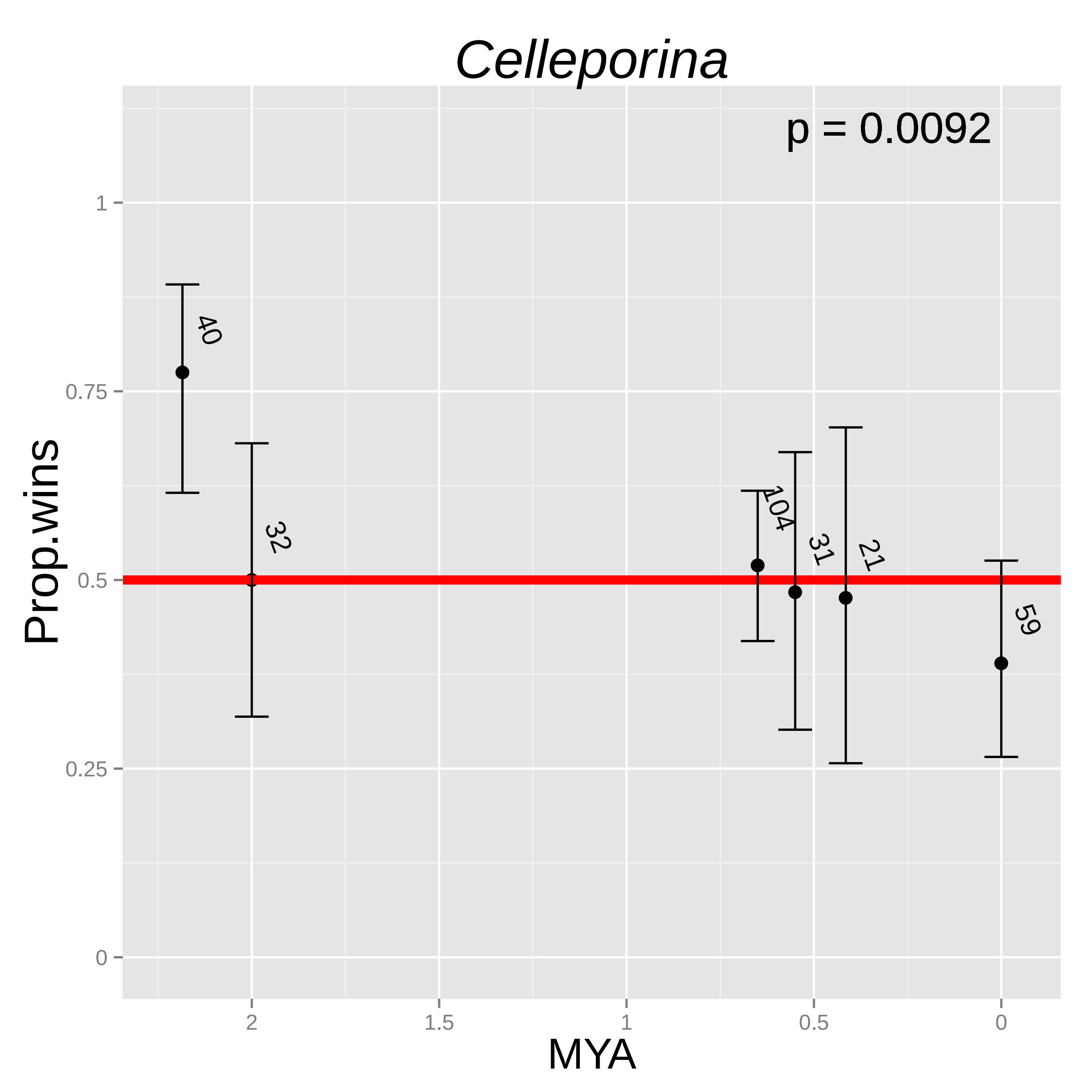

Celleporina
p = 0.0092
Prop.wins
1
0.75
0.5
0.25
0
40
32
104
31
21
59
2
1.5
1
0.5
0
MYA

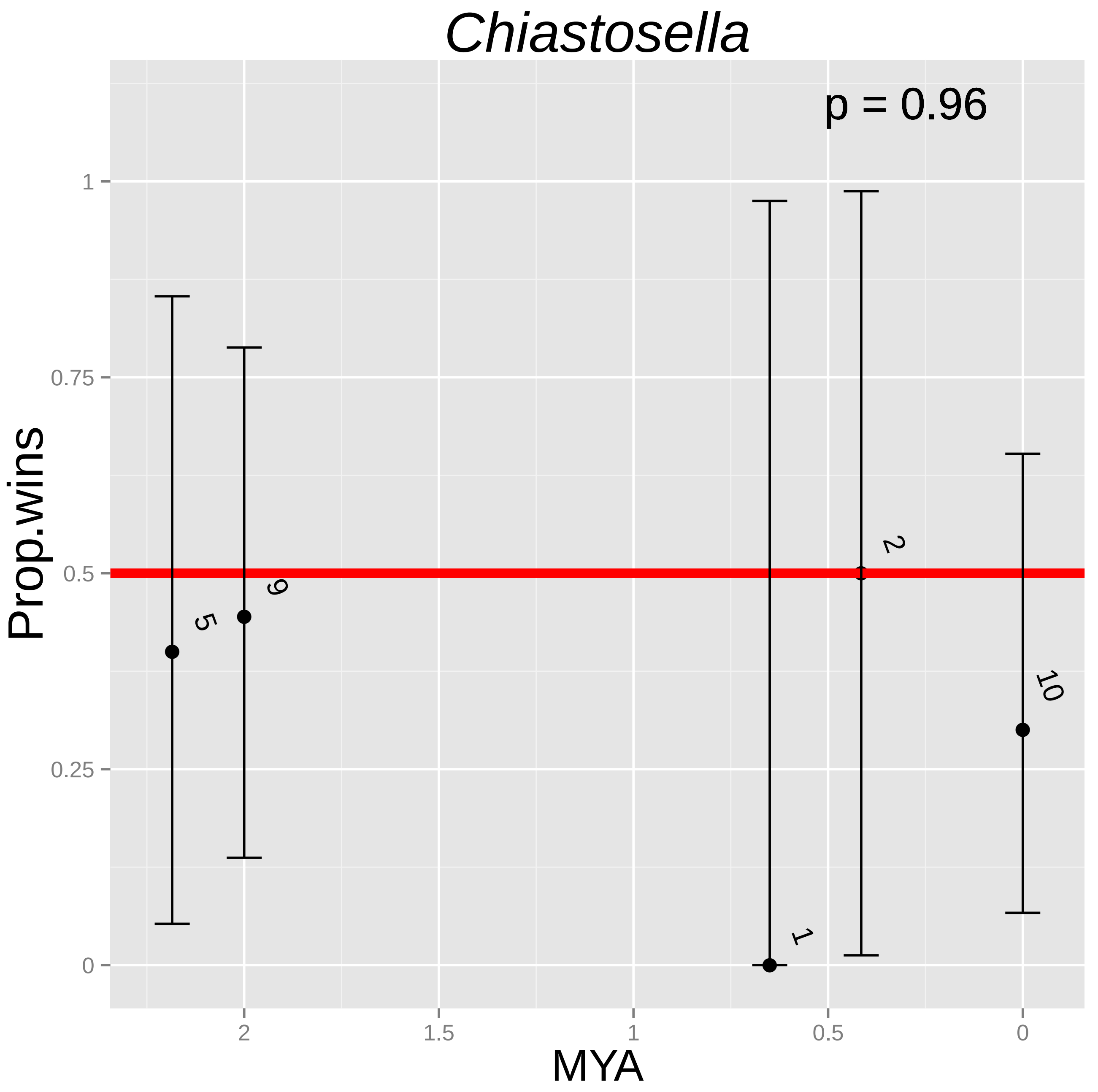

Chiastosella
p = 0.96
Prop.wins
MYA
1
0.75
0.5
0.25
0
2
1.5
1
0.5
0
5
9
1
2
10

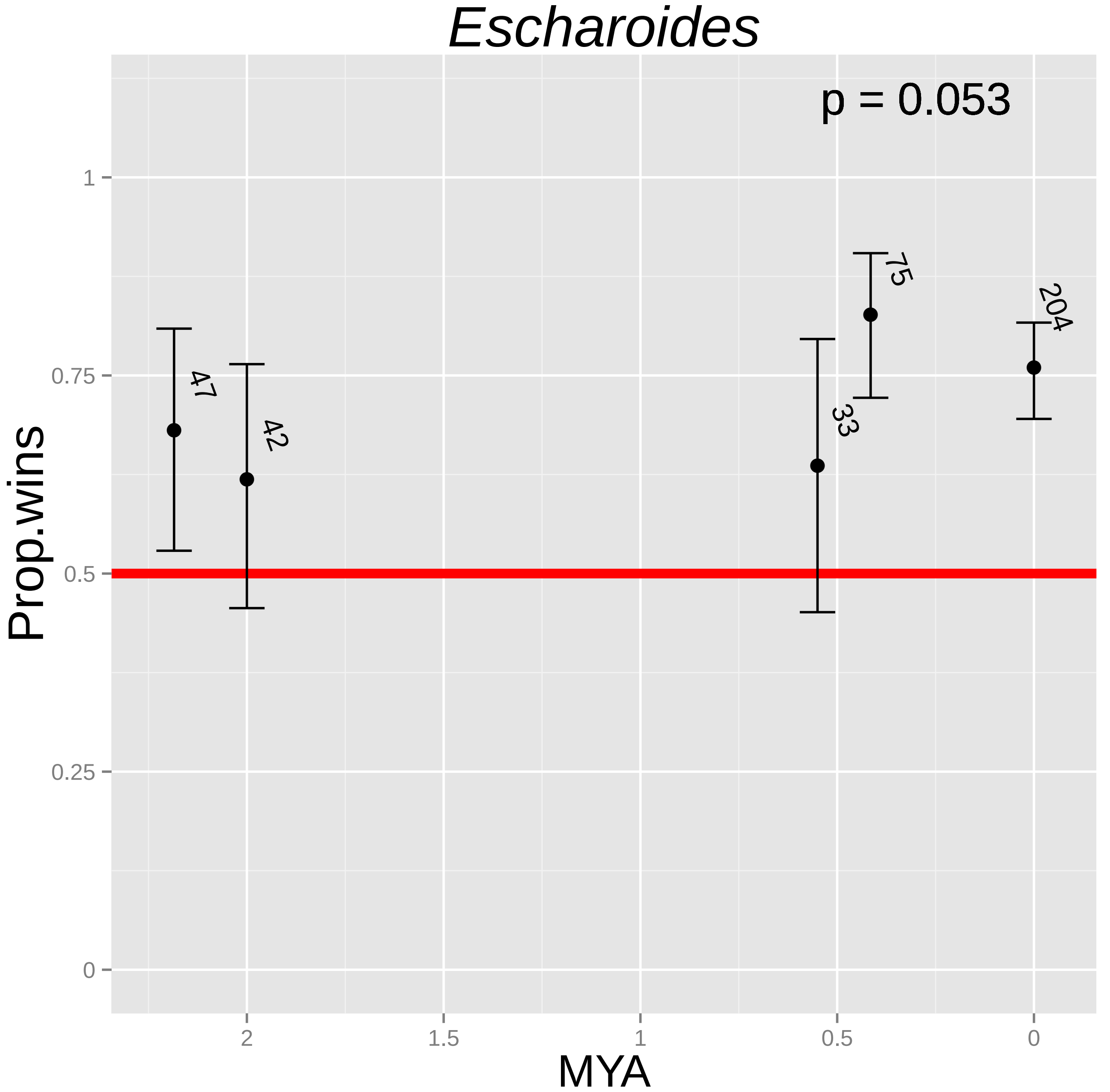

Escharoides
p = 0.053
Prop.wins
MYA
1
0.75
0.5
0.25
0
2
1.5
1
0.5
0
47
42
33
75
204

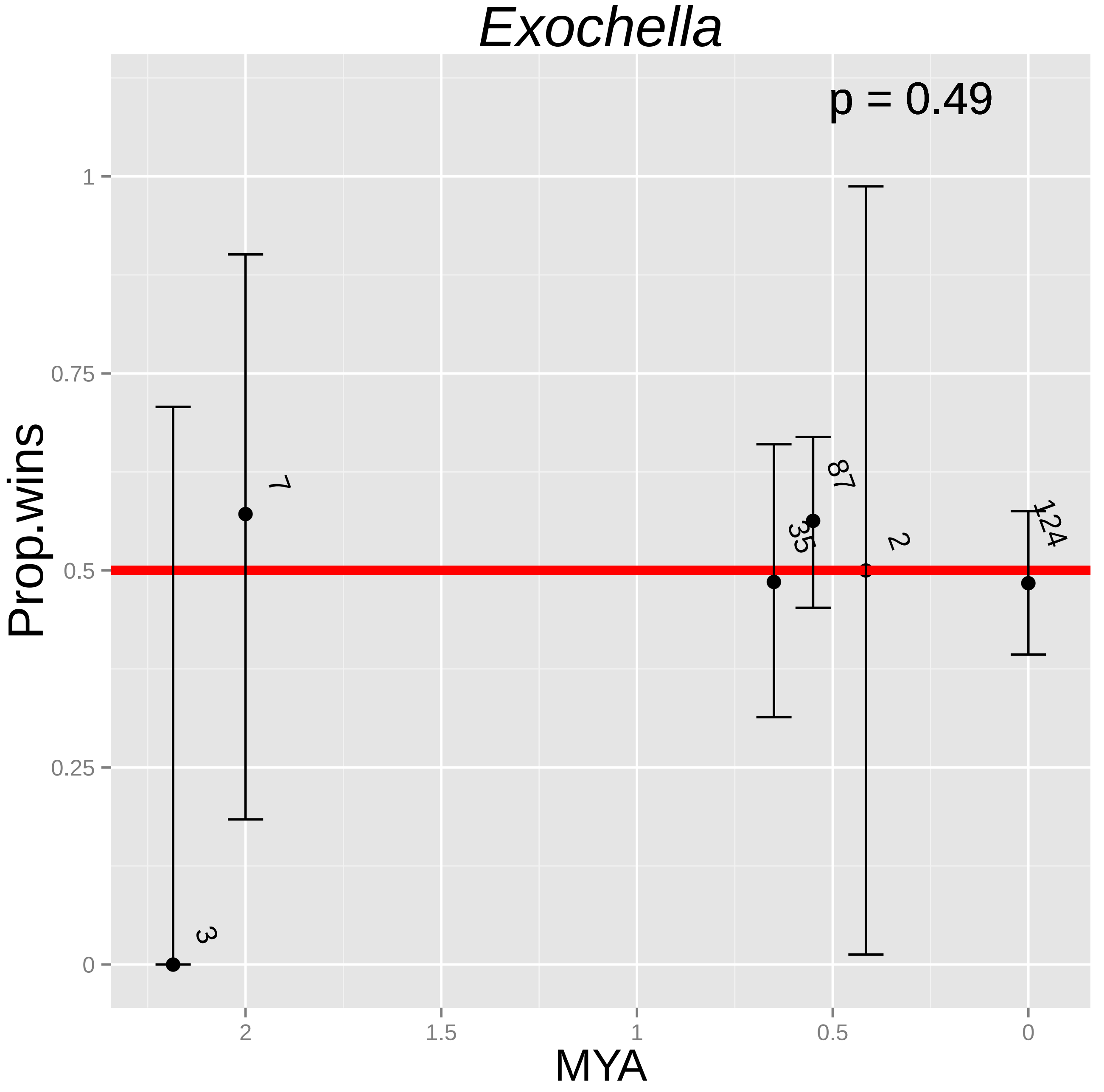

Exochella
p = 0.49
Prop.wins
MYA
1
0.75
0.5
0.25
0
2
1.5
1
0.5
0
3
7
35
87
2
124

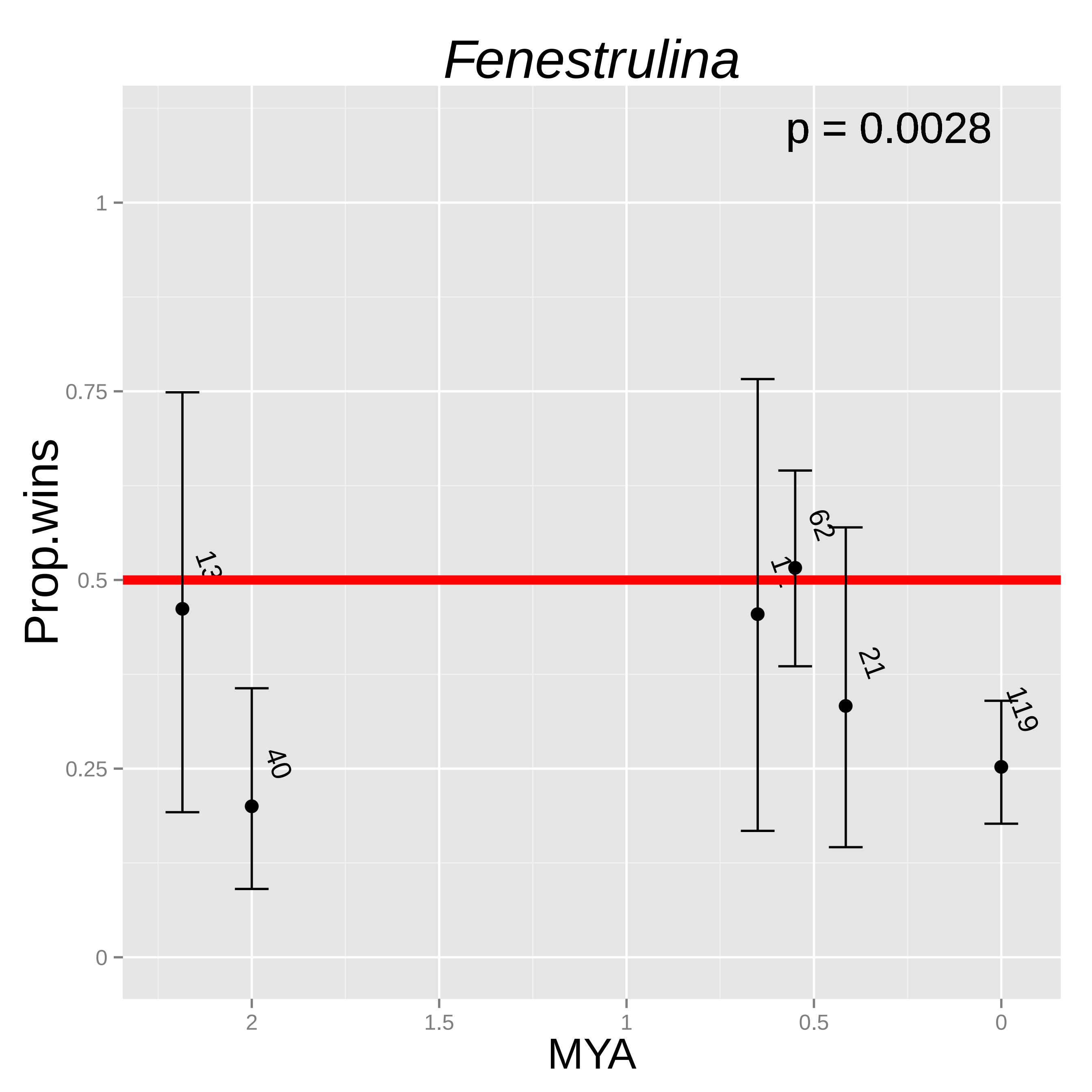

Fenestrulina
p = 0.0028
Prop.wins
MYA
1
0.75
0.5
0.25
0
2
1.5
1
0.5
0
13
40
62
21
119

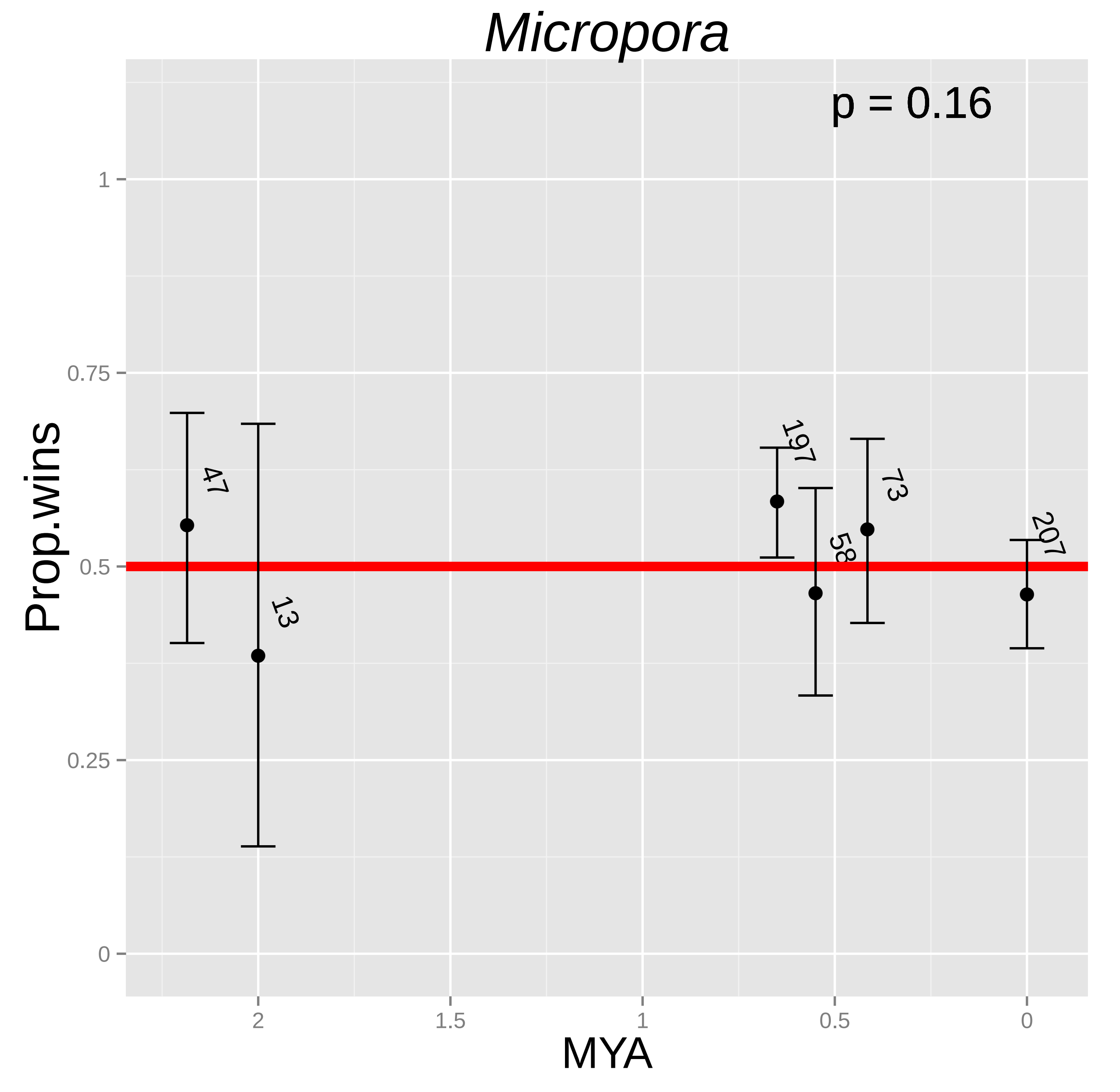

Micropora
p = 0.16
Prop.wins
MYA
1
0.75
0.5
0.25
0
2
1.5
1
0.5
0
47
13
197
58
73
207

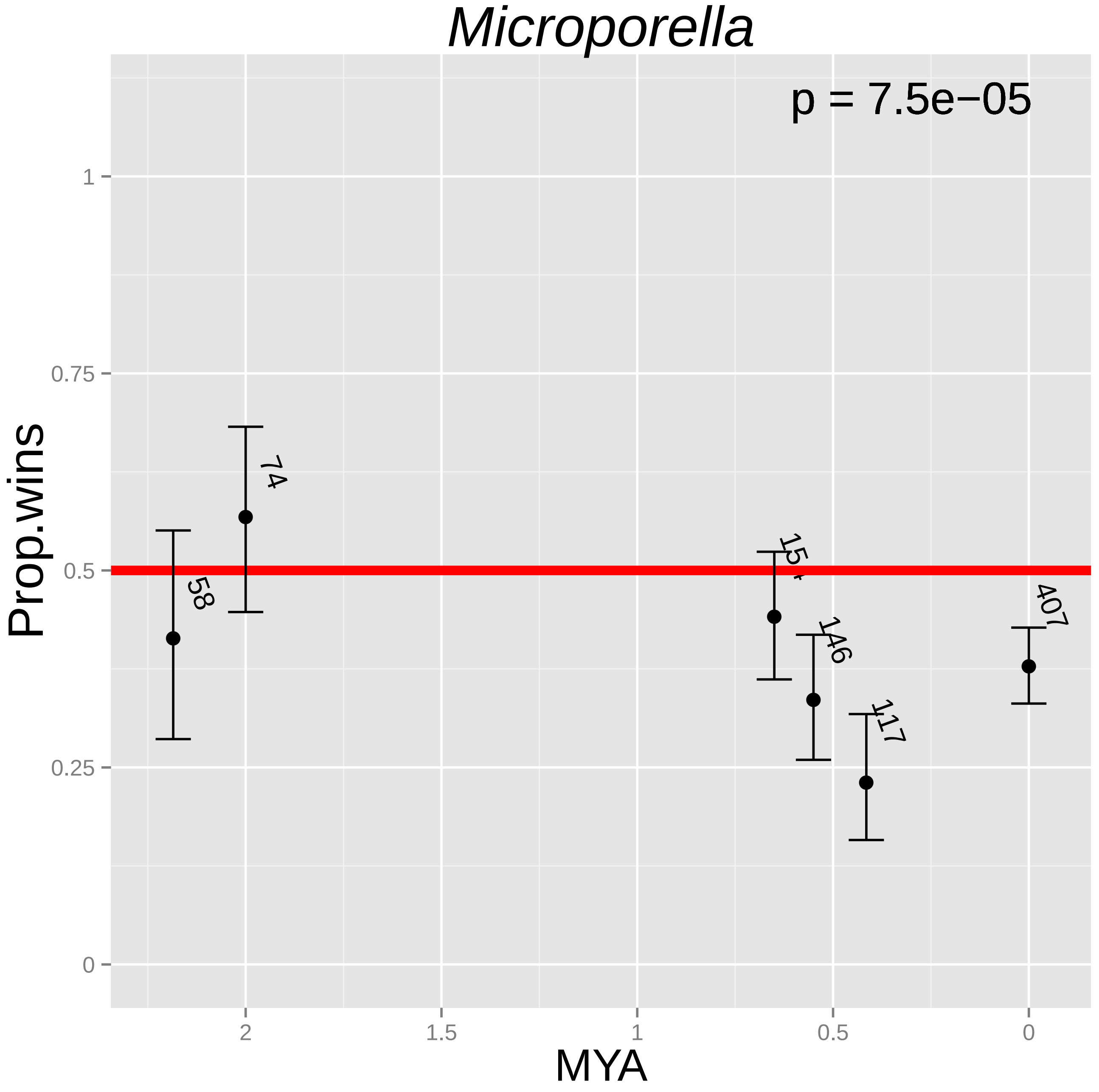
Microporella
p = 7.5e−05
Prop.wins
MYA
1
0.75
0.5
0.25
0
2
1.5
1
0.5
0
74
58
146
117
407

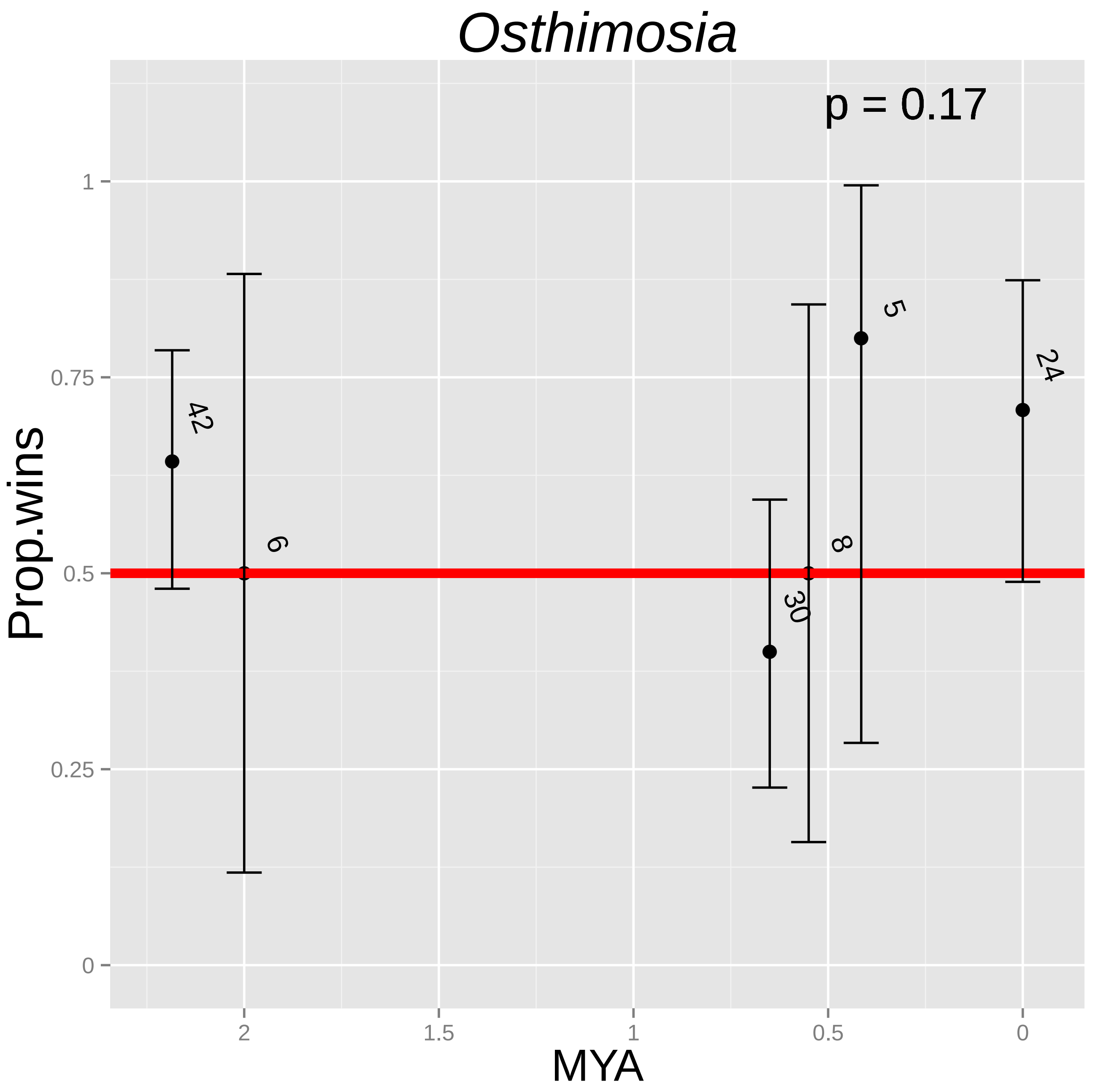

Osthimosia
p = 0.17
Prop.wins
0
0.25
0.5
0.75
1
MYA
2
1.5
1
0.5
0
42
6
30
8
5
24

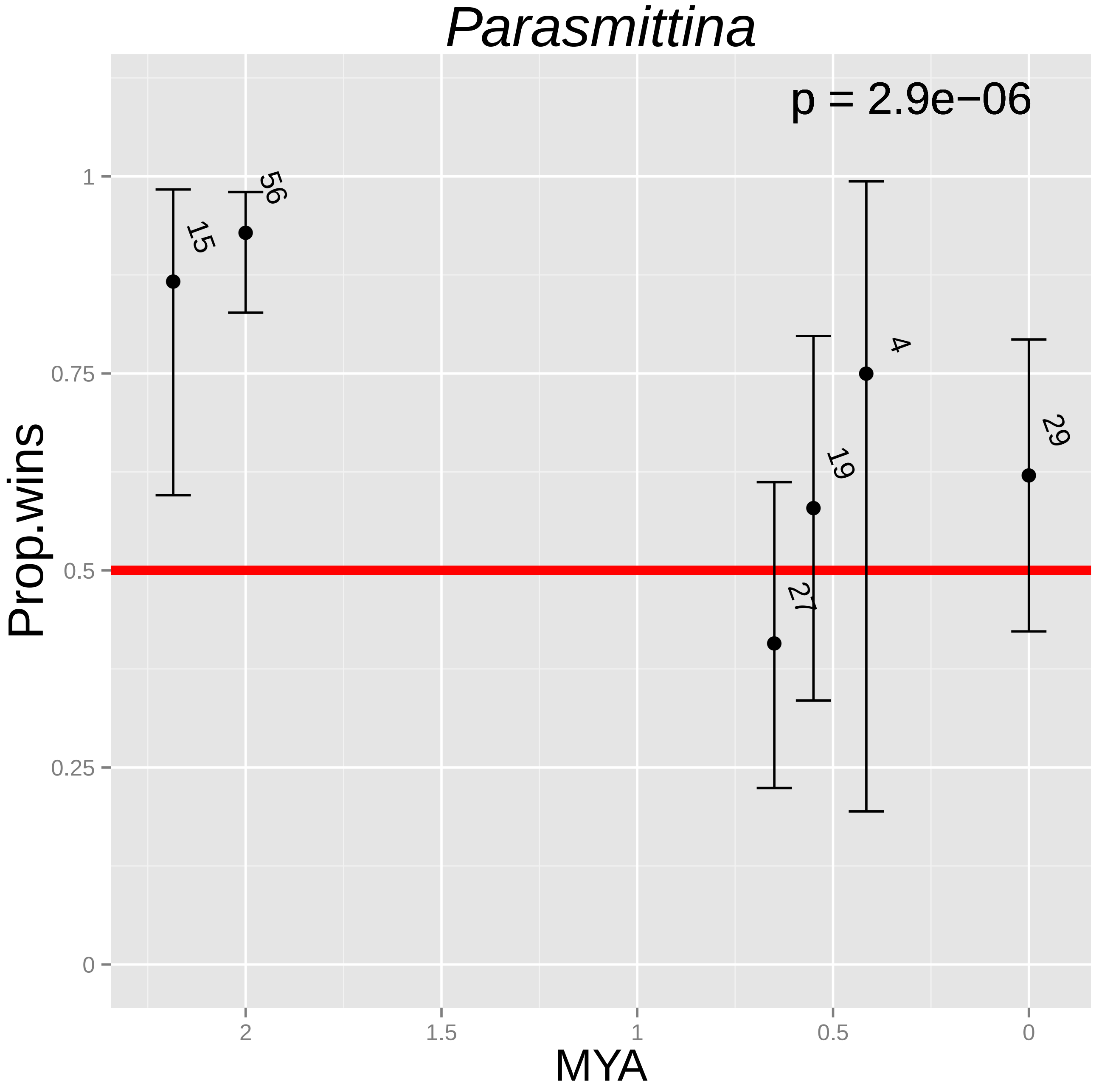

Parasmittina
p = 2.9e−06
Prop.wins
MYA
1
0.75
0.5
0.25
0
2
1.5
1
0.5
0
15
56
4
19
29
27

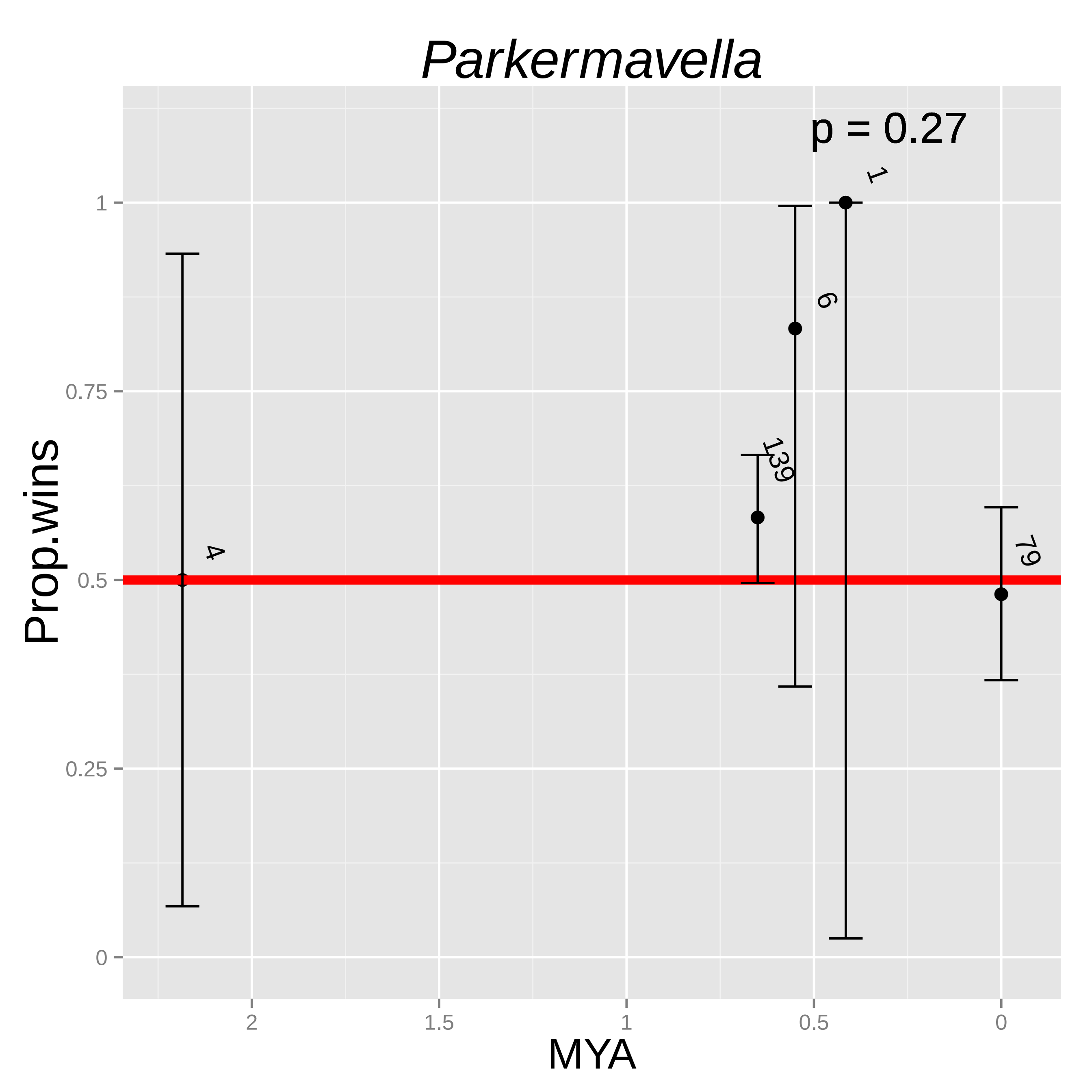

Parkermavella
p = 0.27
Prop.wins
MYA
1
0.75
0.5
0.25
0
2
1.5
1
0.5
0
4
139
6
1
79

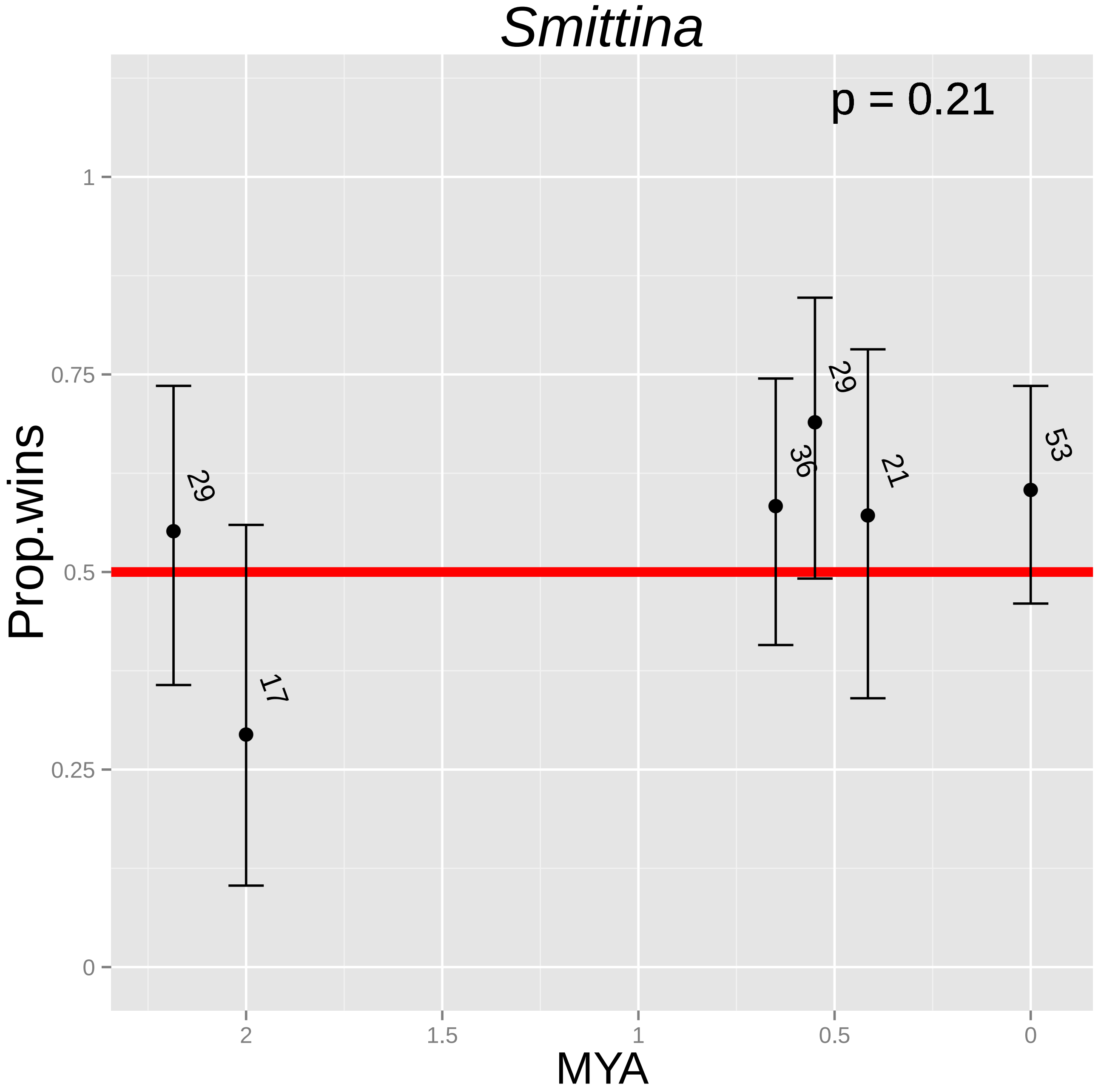
Smittina
p = 0.21
Prop.wins
MYA
1
0.75
0.5
0.25
0
2
1.5
1
0.5
0
29
17
36
29
21
53

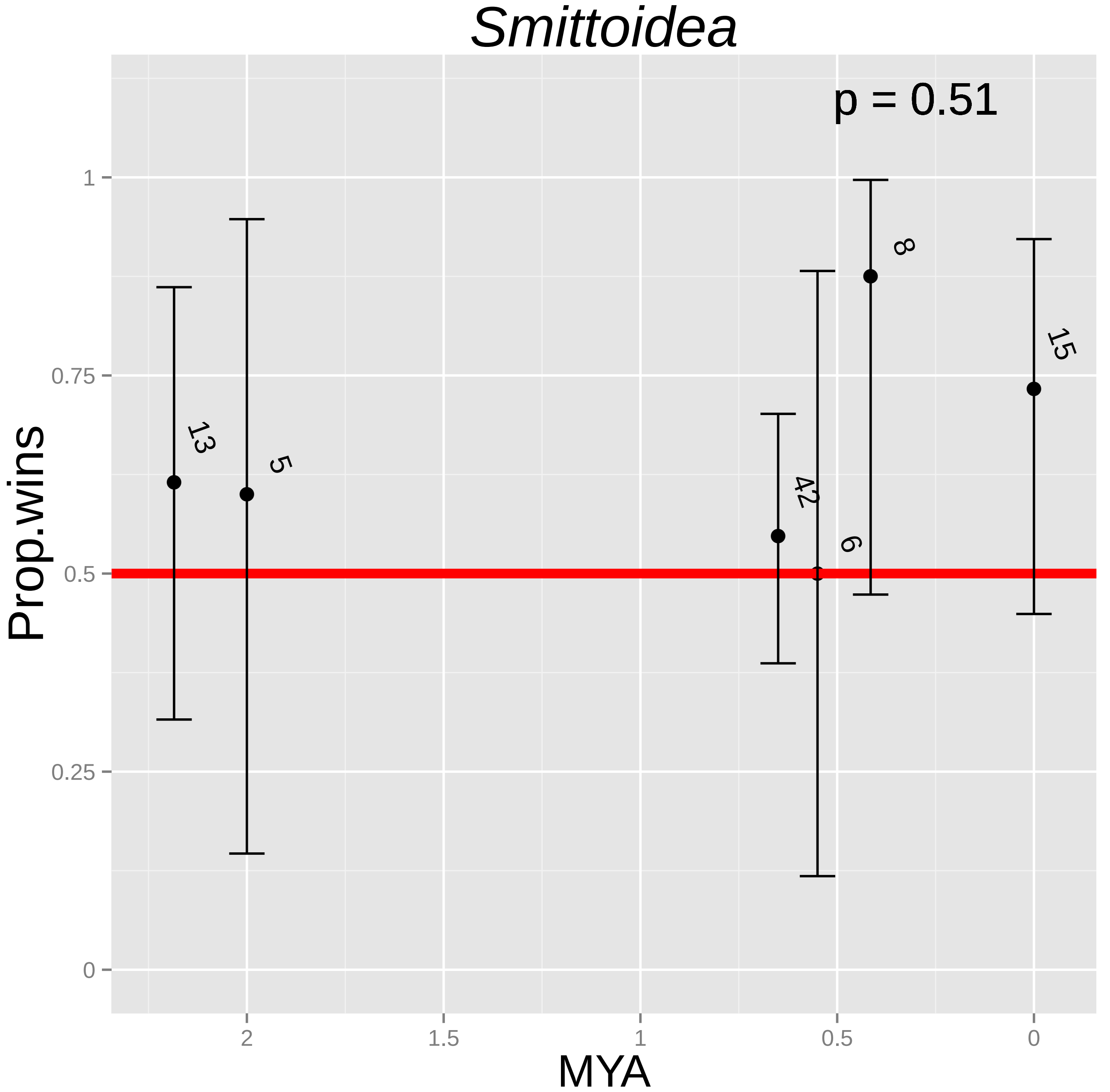

Smittoidea
p = 0.51
Prop.wins
1
0.75
0.5
0.25
0
13
5
42
6
8
15
2
1.5
1
0.5
0
MYA

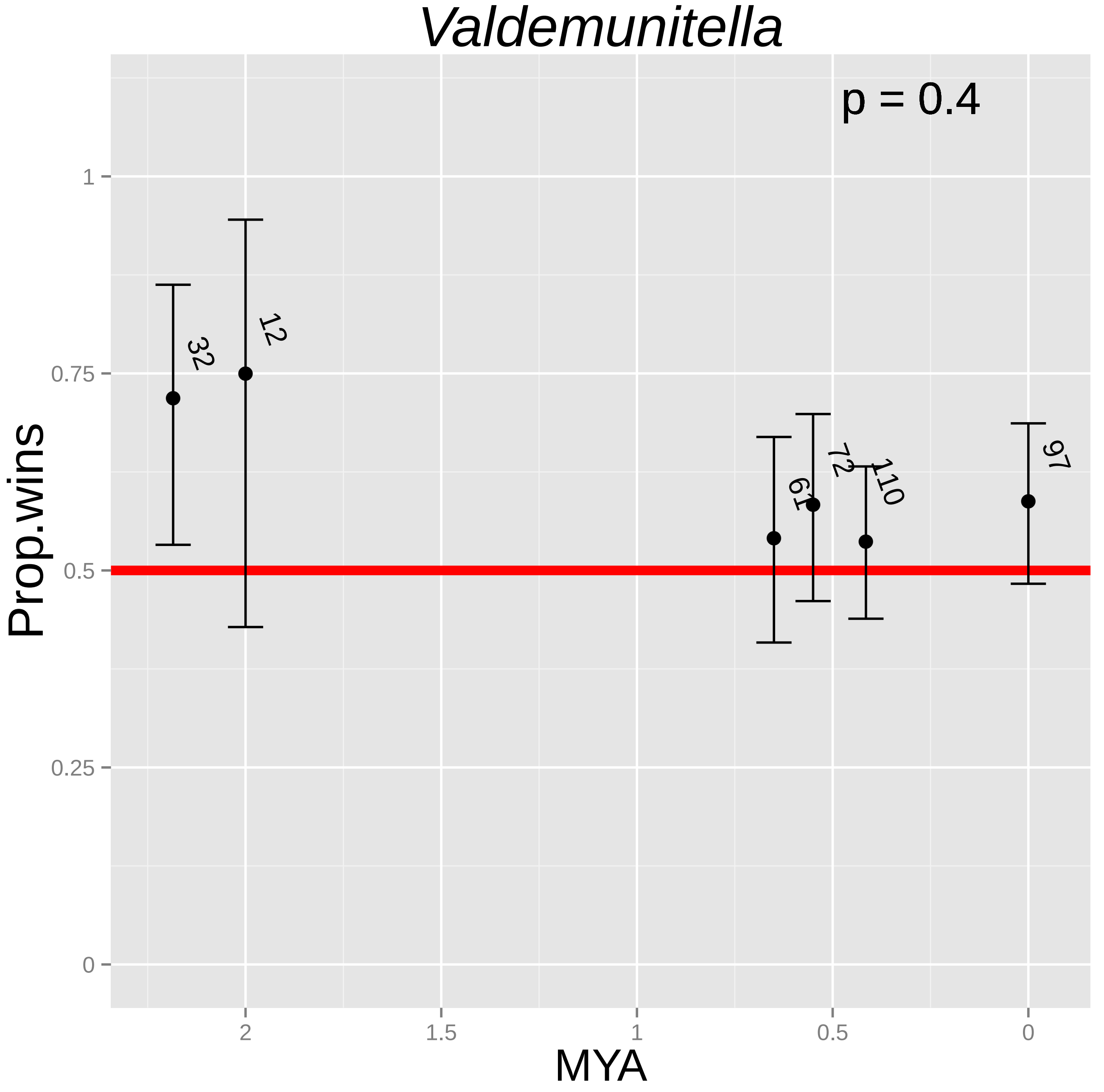
Valdemunitella
p = 0.4
1
0.75
0.5
0.25
0
Prop.wins
32
12
61
72
110
97
2
1.5
1
0.5
0
MYA

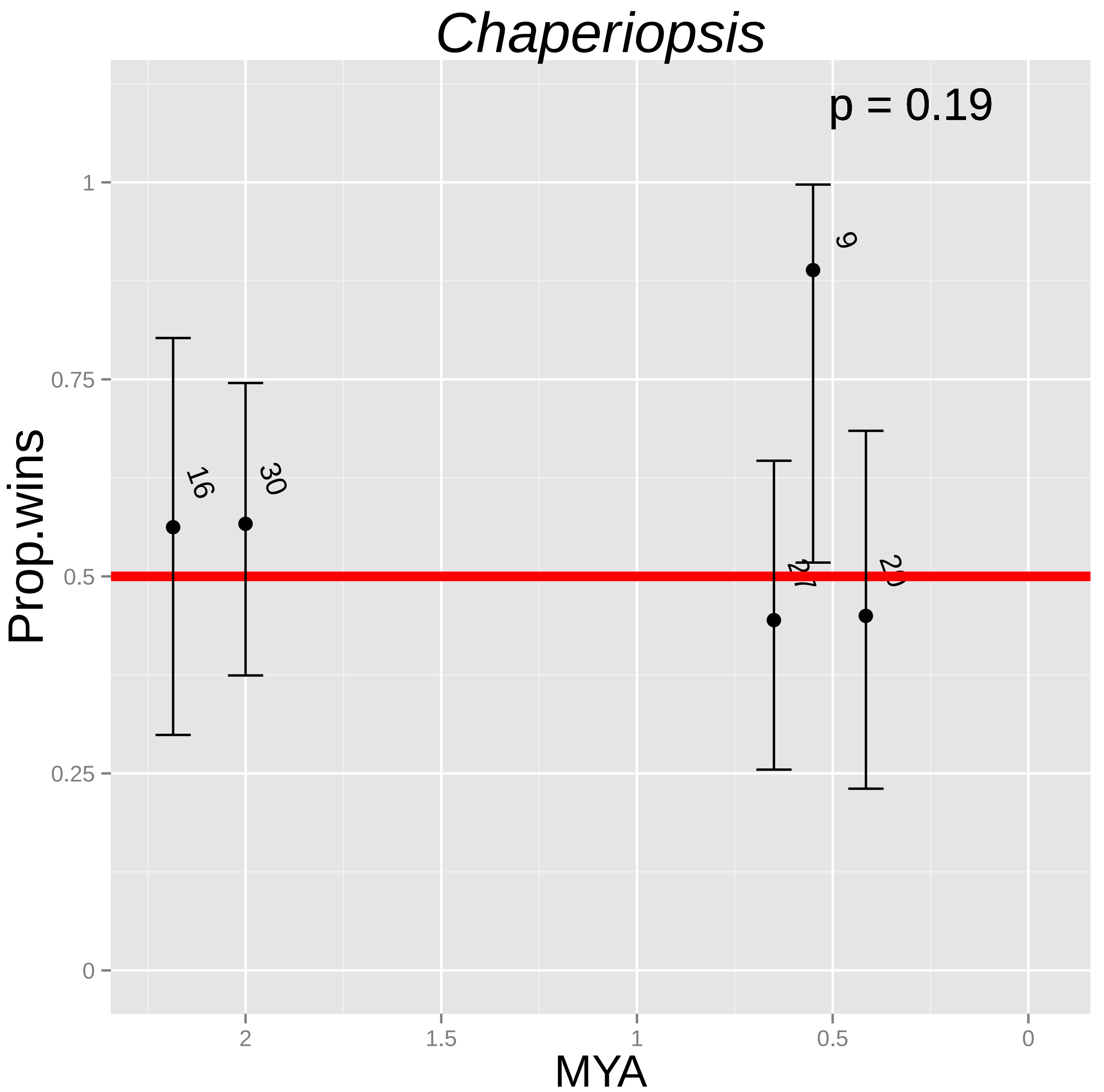
Chaperiopsis
p = 0.19
Prop.wins
MYA
1
0.75
0.5
0.25
0
2
1.5
1
0.5
0
16
30
9
27
20

**Fig. S3: Genus versus species competition dynamics: *Smittina***

As in Fig. 2, panels plot the binomial probabilities (circles) and 95% confidence intervals (lines) of interspecific win-proportions. Black lines and circles indicate win-proportions for all *Smittina* regardless of species while grey dotted lines and open circles indicate the win-proportions of the species named. *Smittina* as a genus seems to have somewhat improved in its competitive ability, although this signal seems to stem mainly from *S. torques* while *S. purpurea* has largely been a loser through the 2Ma analysed. If win-proportions are tabulated using the species means (red in panel D.), then the mean competitive ability of *Smittina* is lower on average than if they are tabulated from all *Smittina* interspecific interactions, with the exception of samples from the Lower Castlecliff Shellbed (*c.* 0.58-0.52 MYA).

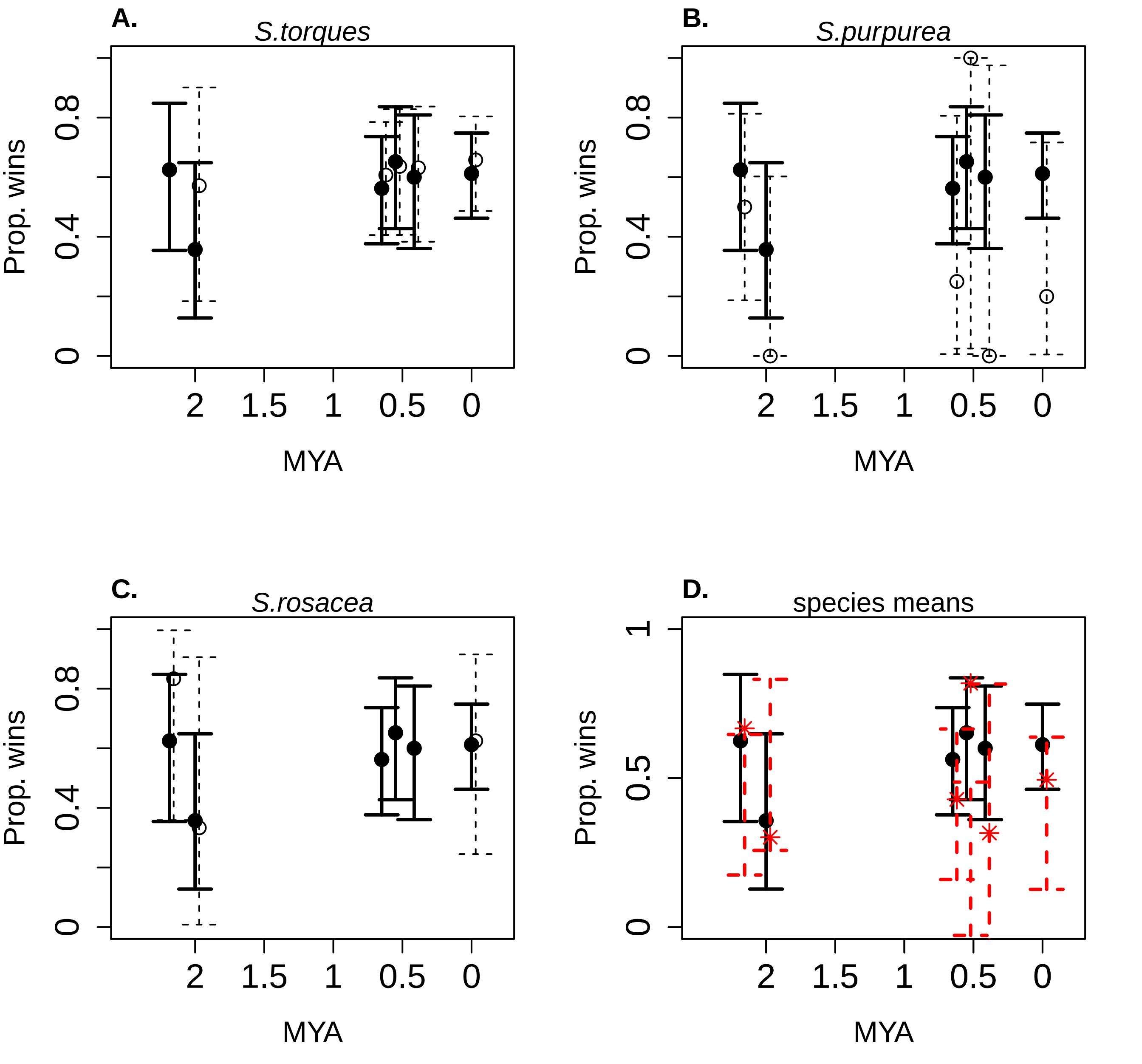

A.
S.torques
B.
S.purpurea
C.
S.rosacea
D.
species means
Prop. wins
MYA
0
0.4
0.8
0.5
1
2
1.5
0.5
